# Discovery of penicillic acid from mangrove endophytic fungi: anti-*Vibrio* screening and cell envelope disruption in *V. parahaemolyticus*

**DOI:** 10.3389/fmicb.2026.1939258

**Published:** 2026-08-31

**Authors:** Yaqi Chen, Fan An, Yuying Chen, Xiangxi Yi, Chenghai Gao, Yuman Gan, Meng Bai

**Affiliations:** 1 Guangxi Key Laboratory of Marine Drugs, Guangxi University of Chinese Medicine, Nanning, China; 2 University Engineering Research Center of High-Efficient Utilization of Marine Traditional Chinese Medicine Resources, Nanning, China; 3 Technology Innovation Center for the Development and Utilization of Marine Drugs and Bioproducts for Beibu Gulf, MNR, Nanning, China; 4 Institute of Marine Drugs, Guangxi University of Chinese Medicine, Nanning, China

**Keywords:** anti-*vibrio* activity, *Aspergillus*, cell-envelope integrity, mangrove endophytic fungi, penicillic acid, *Vibrio parahaemolyticus*

## Abstract

Mangrove endophytic fungi are recognized as prolific sources of structurally diverse and bioactive natural products. In this study, 19 cultivable endophytic fungal strains were isolated and taxonomically characterized from mangrove plant tissues collected at the Shankou Mangrove Nature Reserve (Guangxi, China). One isolate, *Aspergillus sclerotiorum* BM19, exhibited broad-spectrum inhibitory activity against multiple aquatic pathogenic *Vibrio* strains. Bioactivity-guided fractionation of its ethyl acetate crude extract led to the isolation of penicillic acid (1), whose structure was fully elucidated by 1D/2D NMR and HRESIMS. Penicillic acid showed potent anti *Vibrio* parahaemolyticus activity with a minimum inhibitory concentration (MIC) of 6.25 μg/mL and displayed no obvious cytotoxicity toward human HaCaT keratinocytes. Growth curve assays revealed a concentration-dependent antibacterial effect: 0.5 × MIC delayed logarithmic growth, whereas 1 × and 2 × MIC completely suppressed bacterial proliferation. Transmission electron microscopy (TEM) visualized cellular distortion, compromised cell envelopes, and cytoplasmic leakage at MIC, with severe envelope disruption at 2 × MIC. Confocal laser scanning microscopy (CLSM) combined with SYTO 9/PI double staining further confirmed the concentration-dependent impairment of cell envelope integrity, as evidenced by reduced green fluorescence (intact cells) and increased red fluorescence (damaged cells). Collectively, these findings indicate that penicillic acid inhibits V. parahaemolyticus and is associated with concentration-dependent impairment of cell-envelope integrity. Although penicillic acid is a known mycotoxin, its selective activity against pathogenic *vibrios* and low toxicity to human keratinocytes highlight its potential as a lead compound for aquaculture applications. Nevertheless, further systematic evaluation of *in vivo* efficacy and biosafety is required prior to translational development.

## 1 Introduction

*Vibrio* species, ubiquitous Gram-negative bacteria in marine and estuarine environments, pose a growing threat to environmental, human, and animal health (Doni et al., 2025). Among these, *Vibrio parahaemolyticus, V. vulnificus, V. alginolyticus*, and non-O1/non-O139 *V. cholerae* are clinically significant pathogens responsible for gastroenteritis, wound infections, and septicemia in humans, primarily through seafood consumption or seawater exposure (Baker-Austin et al., 2024). Driven by coastal warming and the expansion of seafood trade, the public health burden of pathogenic *vibrios* is projected to rise. Concurrently, *vibriosis* is one of the most widespread bacterial diseases in global aquaculture, causing substantial economic losses across fish, shrimp, shellfish, and mollusks (Mishra et al., 2024). A prominent example is acute hepatopancreatic necrosis disease (AHPND) caused by *V. parahaemolyticus*, which has led to a 60% decline in shrimp production and 43 billion dollars in losses to the global shrimp farming industry (Sen et al., 2026). High-density aquaculture and environmental stressors further exacerbate vibriosis outbreaks (Lai et al., 2026).

Although antibiotics remain a primary tool for disease control, their overuse has led to widespread antimicrobial resistance (AMR), drug residues, and ecological disruptions. A study of 304 *V. parahaemolyticus* isolates from imported seafood found that 9.1% were resistant to at least one antibiotic class, with highest resistance to tetracycline (14.5%) and trimethoprim-sulfamethoxazole (3.6%) (Bourdonnais et al., 2024). This resistance is mediated by antimicrobial resistance genes (ARGs), and evidence indicates *V. parahaemolyticus* can actively acquire and disseminate ARGs (Fang et al., 2023). This challenge has spurred a continued search for new anti-*Vibrio* agents from environmentally compatible and biodiverse sources (Huang et al., 2022).

Natural products have long served as a rich source of antimicrobial leads. Marine-derived fungi, in particular, have attracted considerable attention due to their chemical diversity and ecological adaptability. Among them, marine-derived *Aspergillus* species have yielded numerous antibacterial scaffolds and represent a productive source for natural product discovery (Nazir et al., 2024; Wang et al., 2024). Mangrove ecosystems, with their unique tidal, saline, and anoxic conditions, exert strong selective pressures on resident microbes, fostering specialized metabolic potential (Liu et al., 2026). Consequently, mangrove-associated endophytic fungi have emerged as prolific producers of structurally diverse bioactive metabolites, including antibacterial, antibiofilm, and antioxidant compounds. Recent investigations have extensively explored marine-derived antimicrobial agents against aquatic pathogenic *Vibrio* species. Antimicrobial peptides (AMPs) such as Larimicin78-102 from large yellow croaker exhibit broad-spectrum bactericidal and anti-biofilm activities against multiple *Vibrio* pathogens, while showing no obvious cytotoxicity and significantly improving host survival *in vivo* (Zhou et al., 2025). Similarly, the marine-sourced AMP Ajapocin, identified through sequence optimization, displays potent activity against *V. parahaemolyticus* and *V. vulnificus* with MIC values of 6–12 μM, comparable to polymyxin B, and demonstrates therapeutic efficacy in both zebrafish and mouse infection models without hepatic or renal toxicity (Wang et al., 2026). Beyond peptide-based agents, small molecule natural products have also shown promise: compounds 2 and 3 from marine-derived *Streptomyces* sp. ZZ741A exhibit specific anti-*Vibrio* activity with MIC values ranging from 8 to 128 μg/mL, with molecular docking suggesting outer membrane protein U (OmpU) as a potential target (Zhang et al., 2025). Three new α-pyrones, phomones A–C, from sponge-associated *Phoma* sp. NBUF235 selectively inhibit six aquatic *Vibrio* species with MIC values of 8–32 μg/mL, enriching the chemical diversity of anti-*Vibrio* leads from marine mesophotic zone fungi (Lai et al., 2026). Additionally, optimization of culture conditions for marine *Streptomyces* SK3 has enabled production of terpenoid and steroid bioactive metabolites that exhibit strong inhibition against *V. harveyi, V. parahaemolyticus*, and *V. vulnificus* (Khaochamnan et al., 2024). Despite these diverse advances spanning peptides, small molecules, and fermentation optimization, few research workflows combine culture-dependent strain isolation, targeted anti-*Vibrio* bioassay screening, bioactivity-guided purification of active molecules, and phenotypic characterization of membrane-disrupting effects against *V. parahaemolyticus*. Specifically, the anti-*V. parahaemolyticus* activity of penicillic acid and its corresponding cell envelope damage phenotype remain insufficiently characterized within such an integrated screening framework.

Despite these diverse advances, the anti-*Vibrio* activity of penicillic acid and its effect on bacterial cell envelope integrity have not been systematically examined at the phenotypic level. Penicillic acid, a polyketide mycotoxin frequently reported from *Aspergillus* species, is of particular interest because its electrophilic chemical nature could confer either broad non-specific cytotoxicity or selective membrane-targeting activity. Distinguishing between these possibilities is critical for evaluating its potential as a safe anti-*Vibrio* agent; however, no systematic study has yet employed phenotypic imaging to examine its effect on cell envelope integrity in *V. parahaemolyticus*. To address this gap, the present study isolated endophytic fungi from the Shankou Mangrove Nature Reserve, identified the potent strain *Aspergillus sclerotiorum* BM19, and purified penicillic acid via bioactivity-guided fractionation. In addition to evaluating its antibacterial activity, we assessed its cytotoxicity against human HaCaT keratinocytes and employed TEM and CLSM phenotypic imaging to provide phenotypic evidence of cell envelope disruption. This workflow offers a useful framework for the discovery of cell-envelope-targeting natural anti-*Vibrio* agents with acceptable biosafety profiles for aquaculture disease management.

## 2 Materials and methods

### 2.1 Experimental materials

Mature, healthy Rhus chinensis plants were collected from the Hepu Shankou Mangrove Nature Reserve in Guangxi, China (21°28′22″-21°37′00″N, 108°28′35″-108°54′26″E). Using a sterile scalpel, small portions of stems, roots, and leaves were excised from the plants, placed in sterile plastic bags, and stored at 4 °C until use.

### 2.2 Isolation of endophytic fungi

The tissue surface disinfection method was modified based on the experimental protocols of Shen et al. (2023). The roots of the mangrove plants were thoroughly rinsed with clean water, dried with filter paper, and transferred to a clean bench. The mangrove plants. tissue was then rinsed with sterile water for 30 s, immersed in 75% ethanol for 3 min, and rinsed again with sterile water. The tissue was soaked in sterile water for 30 s, then immersed in 2% sodium hypochlorite solution (roots for 3 min, stems for 5 min). Subsequently, it was rinsed with sterile water for 30 s, followed by a 30 second rinse with 75% ethanol, and then a final 30 second rinse with sterile water. Finally, the tissue was blotted dry with sterile filter paper. The stems were peeled and cut into 0.5 cm^3^ pieces. The root epidermis was gently scraped off with a blade, and the roots were cut into 1 cm pieces. The cut tissue pieces were placed on potato dextrose agar (PDA) medium containing 0.03% chloramphenicol. The PDA plates were incubated at 37 °C for 5–10 days. Isolated strains are preserved at the Institute of Marine Medicinal Research, Guangxi University of Chinese Medicine.

### 2.3 Identification and phylogenetic analysis of endophytic fungi

The identification of fungal isolates was performed by combining morphological observation with internal transcribed spacer (ITS) sequence analysis. Total DNA was extracted from all fungal strains using the TIANcombi DNA Lyse & Detection PCR Kit (TIAN Biotech, Beijing, China), strictly following the manufacturer's standard operating procedures for fungal DNA extraction. The near full length ITS sequence was amplified by polymerase chain reaction (PCR) using the ITS1 and ITS4 primers. The PCR amplification protocol consisted of an initial denaturation at 95 °C for 10 min, followed by 30 cycles of 95 °C for 30 s, 55 °C for 30 s, and 72 °C for 1 min and 30 s, with a final extension at 72 °C for 10 min, and then a hold at 4 °C. The PCR products were sent to Shengong Biotechnology Co., Ltd. for sequencing. The resulting sequences were compared using BLAST alignment against the NCBI (National Center for Biotechnology Information) database, and a phylogenetic tree was constructed using the maximum likelihood (ML) method in MEGA 11 software.

### 2.4 Screening of anti-*Vibrio* endophytic fungi

The isolated and purified endophytic fungus was inoculated into a 500 mL conical flask containing 200 mL of potato-dextrose broth (PDB) medium and cultured for 3 days at 37 °C and 180 rpm. Using a sterile Pasteur pipette, 4 mL of the culture was aspirated and inoculated in a circular pattern into a 500 mL conical flask containing 80 g of rice. The culture was left to stand at room temperature for 30 days. A crude extract was obtained by extraction with ethyl acetate. The antibacterial activity of the crude extract against *Vibrio parahaemolyticus* (ATCC 17802), *V. alginolyticus* (ATCC 17749), and *V. owensii* (ATCC 25919) was evaluated using the filter paper diffusion method. The specific method for the preliminary screening of antibacterial activity using the filter paper agar diffusion method is as follows: Sterilized LB agar medium supplemented with 2% NaCl was cooled to an appropriate temperature, inoculated with the bacterial suspension, gently mixed, and poured into 90 mm sterile Petri dishes. Allow it to solidify for 20 min before use. Use forceps to place the sterilized filter paper disk flat in different areas of the same Petri dish containing the pathogenic bacteria. Dissolve the test compounds separately in dimethyl sulfoxide (DMSO) to a concentration of 5 mg/mL. Using a 10 μL pipette, aspirate 5 μL from the compound solution, the positive control solution, and DMSO as the negative control respectively, and dispense them onto the center of each filter paper. Conduct three parallel experiments. After the liquid has completely evaporated, invert all petri dishes and place them in plastic bags. Incubate in a microbiological incubator at 28 °C for 6–8 h until the petri dishes are uniformly covered with pathogenic bacteria, then record the diameter of the inhibition zone.

### 2.5 Isolation and purification of compounds

The fungus was cultured on PDA medium at 37 °C for 3 days. The mycelium was then inoculated into 500 mL conical flasks containing 200 mL of PDB medium and cultured on a rotary shaker at 180 rpm at 37 °C for 3 days to serve as a seed culture. Eight milliliters of the seed culture were inoculated into 1,000 mL conical flasks (*n* = 100), each containing 80 g of rice and 120 mL of water (0.5% sea salt), and the flasks were left to ferment for 30 days for large scale production. The entire culture was extracted twice overnight with an equal volume of ethyl acetate. The collected solvent was concentrated under reduced pressure at 45 °C using a rotary vacuum evaporator to obtain a crude extract.

**Figure 1:**
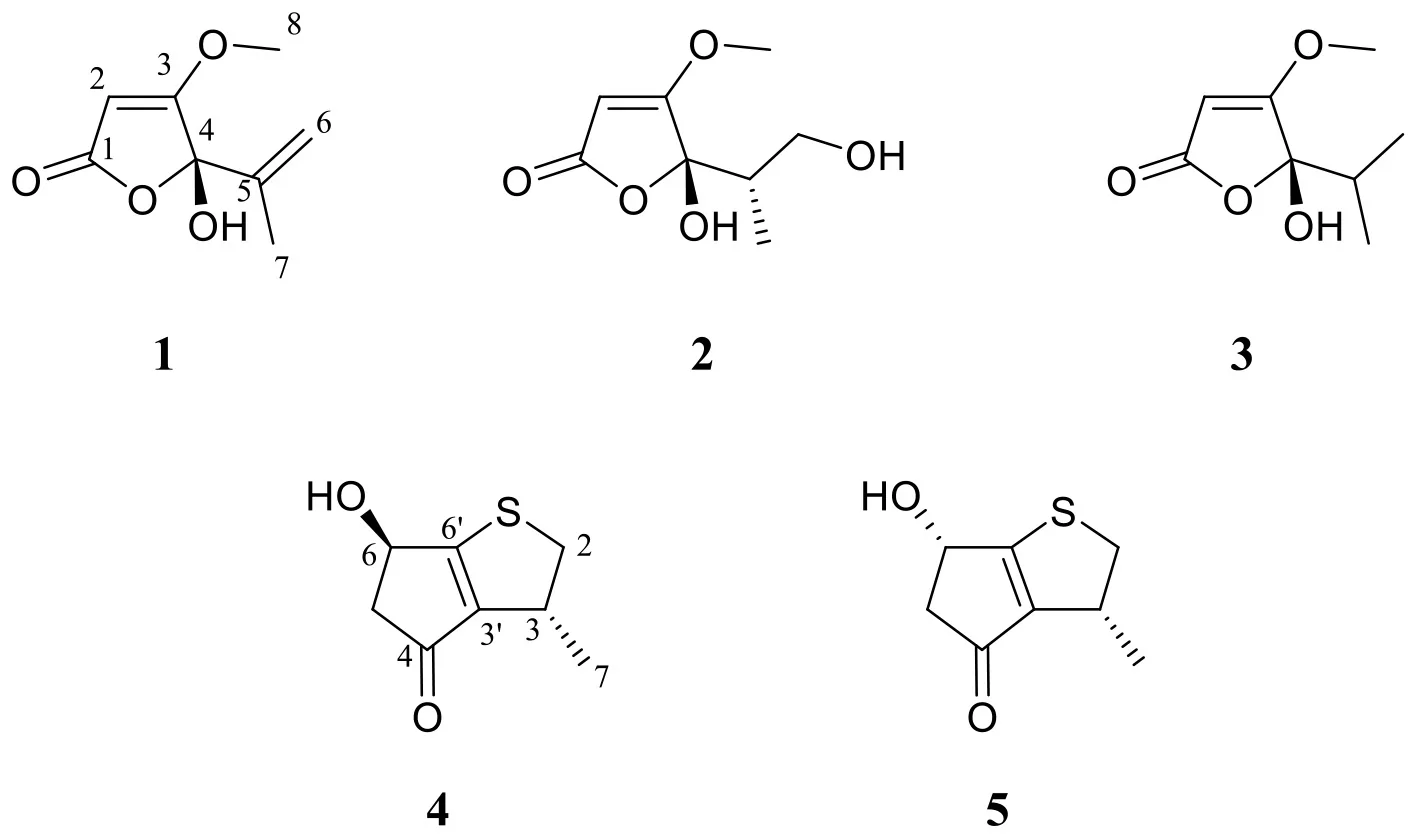
The structures of compounds 1-5.

The crude extract was separated by silica gel column chromatography using gradient elution: first with PET/EtOAc (100:0 to 0:100, v/v), followed by EtOAc/MeOH (3:1 to 0:100, v/v). Target molecules were tracked by TLC, and compounds from each fraction were analyzed and combined. Identical fractions were concentrated to yield 11 fractions, Fr.1-Fr.11. Following a bioactivity guided purification strategy, Fr.6 was separated by medium pressure reverse phase column chromatography using a methanol/water elution system with methanol fractions of 30%, 50%, 70%, 80%, and 100%, at a flow rate of 15 mL/min. After pooling based on TLC profiles, subfractions Fr.6-1 through Fr.6-16 were obtained. Fr.6-3 was subjected to preparative HPLC under isocratic elution conditions with 28% chromatographic grade methanol at a flow rate of 2 mL/min, yielding compound 1 (286.5 mg, *t*_R_ = 21.9 min), 2 (7.0 mg, *t*_R_ = 7.3 min), 3 (9.0 mg, *t*_R_ = 35.8 min), 4 (3.8 mg, *t*_R_ = 15.7 min), and 5 (6.9 mg, *t*_R_ = 17.9 min) (Figure 1).

### 2.6 Determination of Minimum Inhibitory Concentration (MIC)

The MIC method was used to evaluate the antimicrobial activity of 1. The test compound and the positive control drug (norfloxacin) were dissolved separately in DMSO to prepare 1 mg/mL stock solutions. In a 96 well microtiter plate, 190 μL of the diluted bacterial suspension was added to each well in the first row, while 100 μL of the diluted bacterial suspension was added to each well in the remaining rows. Subsequently, 10 μL of test sample solution or positive control solution was supplemented into each well of the first row, bringing the total volume of each well in the first row to 200 μL. After thorough mixing with a multichannel pipette, 100 μL of the uniform mixture was transferred from the first row to the second row for serial two-fold dilution down to the final row. After serial dilution, 100 μL bacterial suspension was supplemented into the last row to make up a total well volume of 200 μL across all wells, generating final compound concentrations of 25, 12.5, 6.25, 3.12, 1.56, 0.78, 0.39, and 0.19 μg/mL. The 96 well plate was incubated at 37 °C for 16 hours, and observations were made every 4 hours. The appearance of sufficient bacterial growth in the negative control well was used as the cutoff point to record and determine the MIC value.

### 2.7 Growth curve

To further clarify the effect of the compound on *Vibrio* growth, *Vibrio parahaemolyticus* was used as the indicator strain to determine the growth curves of 1 at concentrations of 0.5 × MIC, 1.0 × MIC, and 2.0 × MIC. A bacterial suspension in the logarithmic growth phase was adjusted to an OD_600_ of approximately 0.05, and different concentrations of the compound were added, with LB medium containing 1% DMSO serving as the blank control. The cultures were incubated at 28 °C, the OD_600_ value was measured every 0.5 h, and monitoring was continued for 12 h. The growth curve was plotted with time on the x axis and absorbance on the y axis.

### 2.8 Transmission Electron Microscopy (TEM) observation of compound effects on pathogenic bacterial morphology

To investigate the morphological changes of *Vibrio* sp. following treatment with compound **1**, samples were prepared as follows: Briefly, an adjusted bacterial suspension (1 × 10^8^ CFU/mL) was incubated with the test compound at concentrations of 0 (control), 1 × MIC, and 2 × MIC. The cultures were shaken at 180 rpm and incubated at 37 °C for 2 h. After incubation, the samples were centrifuged at 5,000 r/min for 15 min at 4 °C. The bacterial pellets were collected and washed twice with pre-chilled phosphate-buffered saline (PBS, pH 7.4). The cells were then fixed overnight at 4 °C in a 2.5% (v/v) glutaraldehyde solution. Following fixation, the cells were washed twice with PBS by centrifugation. A drop of the bacterial suspension was placed onto a copper grid, stained with 2% phosphotungstic acid (PTA) for 30 s, and then allowed to air dry at room temperature. The structural changes of the bacterial cells were observed using transmission electron microscopy (TEM).

### 2.9 SYTO 9/PI dual fluorescence staining

The effect of **1** on the cell membrane integrity of Vibrio parahaemolyticus was evaluated using SYTO 9/PI staining. Bacterial suspensions at the logarithmic growth phase were mixed with **1** at final concentrations of 0, 1 × MIC, and 2 × MIC, respectively. The mixtures were incubated at 37 °C with shaking at 180 r/min for 2 h. After incubation, the cultures were centrifuged at 5,000 r/min for 5 min, and the cell pellets were washed by resuspension in an equal volume of sterile PBS. A working solution containing SYTO 9 and PI was prepared at final concentrations of 2.5 μM and 30 μM, respectively. Subsequently, 5 μL of the staining solution was added to 5 μL of the bacterial suspension, gently vortexed, and incubated at 37 °C in the dark for 15 min. After staining, 5 μL of the stained bacterial suspension was placed onto a glass slide, covered with a coverslip, and observed immediately using a laser scanning confocal microscope.

### 2.10 Cytotoxicity assay

HaCaT cells in the logarithmic growth phase were harvested, rinsed twice with 1 × PBS, and digested by adding 300 μL of 0.25% trypsin solution. The culture dish was shaken in a crosswise manner and incubated in the cell incubator for trypsinization. Once most cells detached from the bottom of the dish, complete medium was added to terminate digestion. The bottom of the dish was gently triturated with a pipette, and the cell suspension was collected into a centrifuge tube followed by centrifugation at 1,000 rpm for 3 min. After discarding the supernatant, the cell pellet was resuspended in 1 mL of complete medium. HaCaT cells were seeded into 96-well plates at a density of 2.5 × 10^4^ cells per well. The cell suspension was fully mixed and incubated overnight at 37 °C in a humidified incubator with 5% CO_2_. Drug treatment was performed after complete cell attachment. Three groups were set up, including blank group (medium only), normal control group (solvent-treated cells), and treatment groups with serially diluted compound concentrations, with three replicate wells for each group. The stock solution was serially diluted in culture medium to achieve a final concentration of 40 μM in the first well, followed by two-fold serial dilutions to obtain the desired concentration gradient. The plates were continuously incubated at 37 °C with 5% CO_2_ for 72 h. After incubation, the supernatant in each well was aspirated, and 100 μL of freshly prepared 5 mg/mL MTT solution was added to each well under dark conditions. The plates were further incubated at 37 °C with 5% CO_2_ for 4–6 h. Subsequently, the supernatant was carefully removed, and 100 μL of DMSO was added to each well to fully dissolve formazan crystals by shaking. The absorbance value (OD value) of each well was measured at 490 nm using a microplate reader. The cell viability was calculated using the following formula:

Cell viability (%) = (OD_treatment_-OD_blank_)/(OD_control_-OD_blank_) × 100%

### 2.11 Data analysis

All experiments were performed in triplicate, and results are expressed as mean ± SD. CLSM images were quantified using ImageJ, while TEM were used qualitatively. Statistical analysis was performed using GraphPad Prism 10, with *P* < 0.05 considered statistically significant.

## 3 Results

### 3.1 Isolation and identification of endophytic fungi from mangrove plants

**Figure 2:**
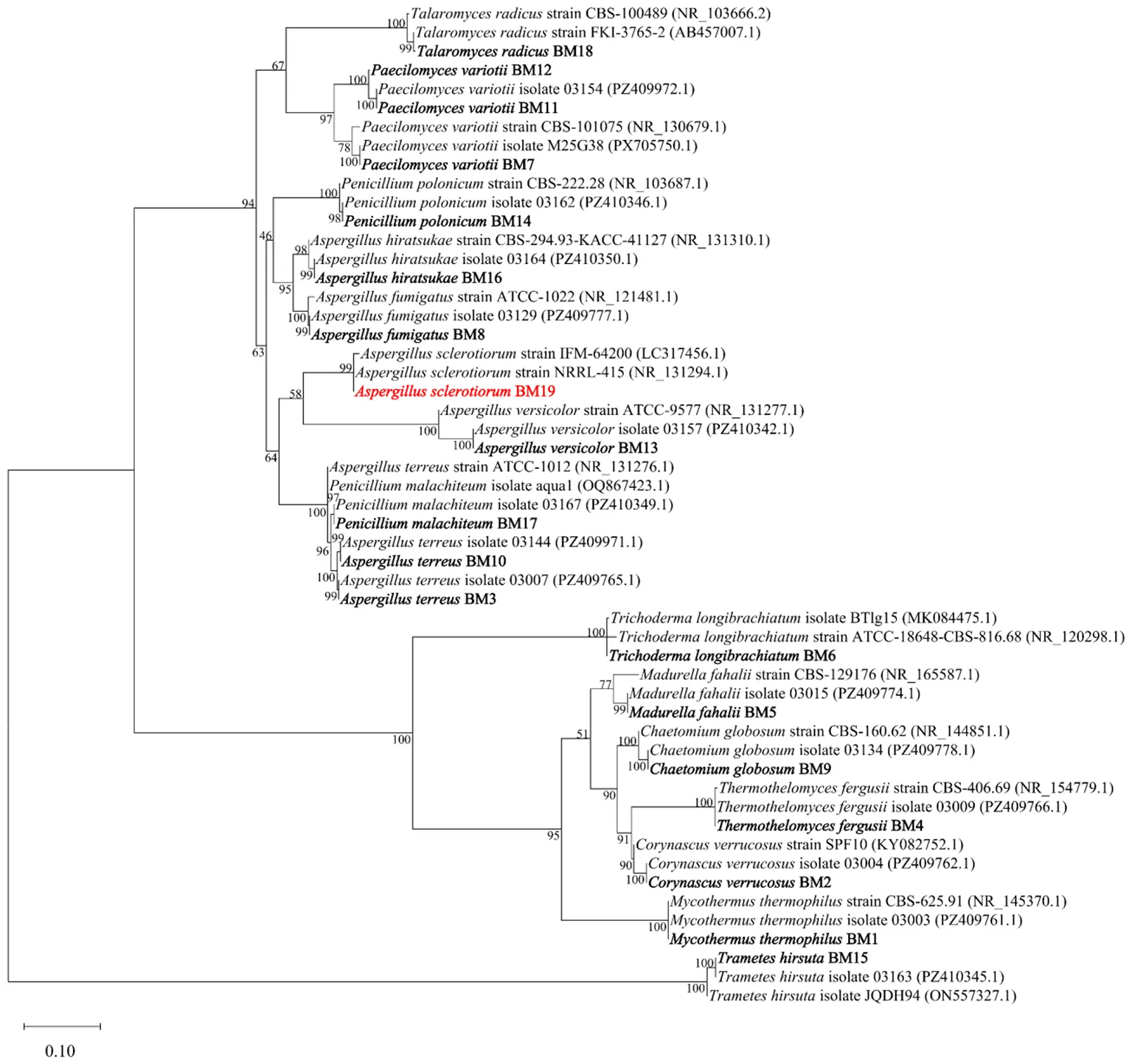
Phylogenetic tree analysis of 19 marine fungal strains.

A total of 19 culturable fungal strains were successfully isolated from mangrove root tissues. All isolates were cultured on potato dextrose agar (PDA) at 37 °C for 5 days to document their macro- and microscopic morphological traits (Supplementary Figures 41, 42). Species identification was achieved through a combination of morphological observation and ITS rDNA sequencing using the universal primer pair ITS1/ITS4. A phylogenetic tree constructed from the ITS sequences clearly resolved the taxonomic affiliations and evolutionary relationships of all strains (Figure 2).

The phylogenetic topology revealed that the 19 isolates span 12 genera across multiple taxonomic lineages, including *Aspergillus, Penicillium, Paecilomyces, Talaromyces, Trichoderma, Mycothermus, Madurella, Chaetomium, Corynascus, Thermothelemyces, Trametes*, and *Hamigera*. Among these, *Aspergillus* was the most frequently recovered genus, comprising six strains: *A. terreus* (BM3, BM10), *A. fumigatus* (BM8), *A. hiratsukae* (BM16), *A. versicolor* (BM13), and *A. sclerotiorum* (BM19), accounting for 31.6% (6/19) of the total isolates. The second most prevalent genus was *Paecilomyces*, represented by three conspecific strains of *P. variotii* (BM7, BM11, BM12), contributing 15.8% (3/19). The genus Penicillium ranked third, with two isolates, *P. malachiteum* BM17, and *P. polonicum* BM14, occupying 10.5% (2/19). Collectively, these three genera—all belonging to the order *Eurotiales*—accounted for 57.9% (11/19) of all isolates, confirming *Eurotiales* as the dominant fungal order in this mangrove root microhabitat.

The predominance of *Aspergillus* and related *Eurotiales* genera in mangrove roots can be attributed to their strong tolerance to the unique mangrove microenvironment, which is characterized by periodic tidal flooding, high salinity, hypoxia, and fluctuating nutrient availability. Fungi from these genera possess robust metabolic plasticity, enabling them to colonize plant root tissues, decompose recalcitrant lignocellulosic substrates, and compete effectively for limited nutrients. Meanwhile, the recovery of rare genera such as *Trametes, Madurella*, and thermophilic species highlights the high fungal biodiversity harbored by mangrove root systems. Overall, this ITS-based phylogenetic analysis systematically clarifies the taxonomic composition and dominant taxa of culturable endophytic fungi from mangrove roots, and confirms that mangrove root tissues represent a rich reservoir of diverse fungal resources with significant potential for bioactive natural product discovery.

### 3.2 *Aspergillus sclerotiorum* BM19 shows greater potential for producing anti-vibrio active compounds

To evaluate the anti-*Vibrio* potential of the 19 isolated mangrove endophytes, their crude extracts were screened against three pathogenic Vibrio species (*V. parahaemolyticus, V. alginolyticus*, and *V. owensii*) using the paper disk diffusion method, with norfloxacin serving as the positive control (Table 1 and Supplementary Figure 43). The results revealed a marked strain-dependent variation in antimicrobial activity. Notably, *Aspergillus sclerotiorum* BM19 exhibited the most potent and broad-spectrum activity, showing inhibition zones preliminary qualitative screening than the positive control against both V. *parahaemolyticus* and *V. owensii*. Additionally, *Talaromyces* sp. BM18, *Paecilomyces variotii* (BM7 and BM12), and *Aspergillus versicolor* BM13 displayed moderate-to-strong inhibition against all three tested pathogens. In contrast, most of the remaining strains exerted either weak or negligible effects, with strains such as BM4 and BM5 demonstrating no activity against any target. These results underscore a significant strain-specific divergence in the biosynthesis of anti-*Vibrio* secondary metabolites, identifying BM19 as the most promising candidate for the development of novel antimicrobial agents in aquaculture.

**Table 1:** Antibacterial activity of fungal crude extracts against pathogenic *Vibrio* strains^a^.

Fungal strain	*V. parahaemolyticus*	*V. alginolyticus*	*V. harveyi*
BM1	/	±	/
BM2	/	±	/
BM3	±	±	/
BM4	/	/	/
BM5	/	/	/
BM6	/	±	/
BM7	+	+	++
BM8	±	±	/
BM9	±	±	/
BM10	±	/	/
BM11	±	±	/
BM12	+	+	+++
BM13	+	++	++
BM14	+	+	/
BM15	±	±	/
BM16	±	/	/
BM17	/	±	/
BM18	++	++	+++
BM19	++++	+	++++

^a^ /, no inhibition zone observed; ±, weak or negligible inhibition; +, visible but moderate inhibition zone; ++, inhibition zone smaller than positive control; +++, inhibition zone equal to positive control; ++++, inhibition zone larger than positive control; +++++, inhibition zone markedly larger than positive control (Positive control: Norfloxacin).

### 3.3 Purification and characterization of bioactive compounds via Anti *Vibrio* activity guided isolation

Preliminary antimicrobial activity testing of the crude extract indicated that *Aspergillus sclerotiorum* BM19 possesses significant bioactive potential. To further characterize its bioactive compounds, an ethyl acetate extract of *Aspergillus sclerotiorum* BM19 was prepared and subjected to bioactivity guided isolation using spectroscopic methods. A total of 214.4 g of crude extract was obtained through large scale fermentation on rice solid medium. Using a bioactivity guided fractionation strategy, the bioactive component compound **1** (286.5 mg) was successfully isolated.

Compound **1** is a white solid, readily soluble in methanol. Its molecular formula was determined to be C_8_H_10_O_4_. HRESIMS: *m*/*z* 171.0632 [M + H]^+^ (calcd for 171.0657) (Supplementary Figure 8). ^1^H NMR (400 MHz, DMSO-*d*_6_) δ_H_: 7.87 (1H, br s, 4-OH), 5.41 (1H, s, H-2), 5.30 (1H, s, H-6a), 5.11 (1H, s, H-6b), 3.88 (3H, s, H-8), 1.65 (3H, s, H-7); ^1^^3^C NMR (100 MHz, DMSO-*d*_6_) δ_C_: 179.4 (C, C-1), 170.0 (C, C-3), 140.1 (C, C-5), 115.3 (CH, C-6), 102.4 (C, C-4), 89.3 (CH, C-2), 59.9 (CH3, C-8), 17.1 (CH3, C-7). Based on comparison with the literature (Phainuphong et al., 2017), compound **1** was identified as penicillic acid.

Compound **2** is a white solid, readily soluble in methanol. Its molecular formula was determined to be C_8_H_12_O_5_. HRESIMS: *m*/*z* 189.0751 [M + H]^+^ (calcd for 189.0763) (Supplementary Figure 16). ^1^H NMR (400 MHz, DMSO-*d*_6_) δ_H_: 7.52 (1H, s, 4-OH), 5.32 (1H, s H-2), 4.53 (1H, t, *J* = 5.3 Hz, 6-OH), 3.85 (1H, s, H-7), 3.38 (1H, dt, *J* = 10.5, 4.6 Hz, H-6), 3.14 (1H, m, H-6), 2.01 (1H, m, H-5), 0.86 (3H, d, *J* = 6.9 Hz, H-8); ^13^C NMR (100 MHz, DMSO-*d*_6_) δ_C_: 179.7 (C, C-3), 170.0 (C, C-1), 104.4 (C, C-4), 89.3 (CH, C-2), 61.2 (CH_2_, C-6), 59.7 (CH_3_, C-7), 41.5 (CH, C-5), 11.2 (CH_3_, C-8). Based on comparison with the literature (Phainuphong et al., 2017), compound **2** was identified as 6-dihydro-6-hydroxy penicillic acid.

Compound **3** is a white solid, readily soluble in methanol. Its molecular formula was determined to be C_8_H_12_O_4_. HRESIMS: *m*/*z* 173.0803 [M + H]^+^ (calcd for 173.0814) (Supplementary Figure 24). ^1^H NMR (400 MHz, DMSO-*d*_6_) δ_H_: 7.44 (1H, s, 4-OH), 5.33 (1H, s, H-2), 3.85 (3H, s, H-8), 2.00 (1H, d, *J* = 6.8 Hz, H-5), 1.05 (3H, d, *J* = 6.8 Hz, H-7), 0.86 (3H, d, *J* = 6.8 Hz, H-6); ^13^C NMR (100 MHz, DMSO-*d*_6_) δ_C_: 179.8(C, C-1), 170.1 (C, C-3), 105.1 (C, C-4), 33.1 (CH, C-5), 89.2 (CH, C-2), 59.9 (CH_3_, C-8), 16.0 (CH_3_, C-7), 15.3 (CH_3_, C-6). Based on comparison with the literature (Phainuphong et al., 2017), compound **3** was identified as 5, 6-dihydropenicillic acid.

Compound **4** is a white solid, readily soluble in methanol. Its molecular formula was determined to be C_8_H_11_O_2_S. HRESIMS: *m*/*z* 171.0455 [M + H]^+^ (calcd for 171.0480) (Supplementary Figure 32). ^1^H NMR (400 MHz, DMSO-*d*_6_) δ_H_: 5.73 (1H, s, H-OH), 4.78 (1H, m, H-6), 3.97 (1H, dd, *J* = 11.5, 9.2 Hz, H-2a), 3.32 (1H, d, *J* = 6.3 Hz, H-2b), 3.17 (1H, m, H-3), 2.94 (1H, dd, *J* = 17.6, 6.1 Hz, H-5a), 2.42 (1H, dd, *J* = 17.6, 2.1 Hz, H-5b), 1.15 (3H, d, *J* = 6.8 Hz, H-7); ^13^C NMR (100 MHz, DMSO-*d*_6_) δ_C_: 194.4 (C, C-4), 184.1 (C, C-6′), 146.0 (C, C-3′), 65.1 (C, C-6), 50.7 (C, C-5), 47.5 (C, C-2), 34.8 (C, C-3), 17.7 (C, C-7). Based on comparison with the literature (Pan et al., 2021), compound **4** was identified as aspergerthinol A.

Compound **5** is a white solid, readily soluble in methanol. Its molecular formula was determined to be C_8_H_11_O_2_S. HRESIMS: *m*/*z* 171.0470 [M + H]^+^ (calcd for 171.0480) (Supplementary Figure 40). ^1^H NMR (400 MHz, DMSO-*d*_6_) δ_H_: 5.74 (1H, s, H-OH), 4.81 (1H, s, H-6), 3.97 (1H, dd, *J* = 11.5, 9.0 Hz, H-2a), 3.31 (1H, d, *J* = 5.5 Hz, H-2b), 3.21 (1H, m, H-3), 2.97 (1H, dd, *J* = 17.6, 6.1 Hz, H-5a), 2.41 (1H, dd, *J* = 17.6, 2.1 Hz, H-5b), 1.10 (3H, d, *J* = 6.8 Hz, H-7); ^13^C NMR (100 MHz, DMSO-*d*_6_) δ_C_: 194.4 (C, C-4), 184.2 (C, C-6′), 146.1 (C, C-3′), 65.2 (C, C-6), 50.7 (C, C-5), 47.5 (C, C-2), 34.6 (C, C-3), 17.5 (C, C-7). Based on comparison with the literature (Pan et al., 2021), compound **5** was identified as aspergerthinol B.

Full sets of 1D and 2D NMR spectra corresponding to each purified metabolite are deposited in the Supplementary Figures 1–40, enabling comprehensive structural verification of all isolated compounds.

### 3.4 MIC and growth curve

In this study, the antibacterial activity of penicillic acid was further evaluated using the broth microdilution method to determine the minimum inhibitory concentration (MIC). Penicillic acid exhibited significant inhibitory effects against all three *Vibrio* strains. Among the tested pathogens, *Vibrio parahaemolyticus* showed the highest susceptibility, with an MIC of 6.25 μg/mL, followed by *V. owensii* with an MIC of 12.5 μg/mL, and *V. alginolyticus* with an MIC of 25 μg/mL. Based on its superior sensitivity, *V. parahaemolyticus* was selected as the indicator strain for further investigation. The study investigated changes in bacterial growth over a 12 h period at different concentration gradients: 0.5 × MIC, 1 × MIC, and 2 × MIC, while simultaneously monitoring OD600 values and plotting growth curves (Supplementary Figure 44).

The results showed that the blank control group (CK) exhibited the fastest growth rate, rapidly entering the logarithmic growth phase after 2 h, with the bacterial concentration continuing to rise and reaching a peak within 12 h. Treatment at 0.5 × MIC significantly inhibited the logarithmic growth of *Vibrio*, with the final bacterial concentration substantially lower than that of the control group. In the high concentration treatment groups (1 × MIC and 2 × MIC), the growth of *Vibrio parahaemolyticus* was nearly completely arrested throughout the entire process, with no significant increase in OD_600_. Penicillic acid inhibited the growth of V. parahaemolyticus in a concentration-dependent manner, with no detectable increase in OD_600_ at 1 × and 2 × MIC during the 12 h observation period.

### 3.5 Effect of compound 1 on cell morphology and membrane integrity of *Vibrio parahaemolyticus*

**Figure 3:**
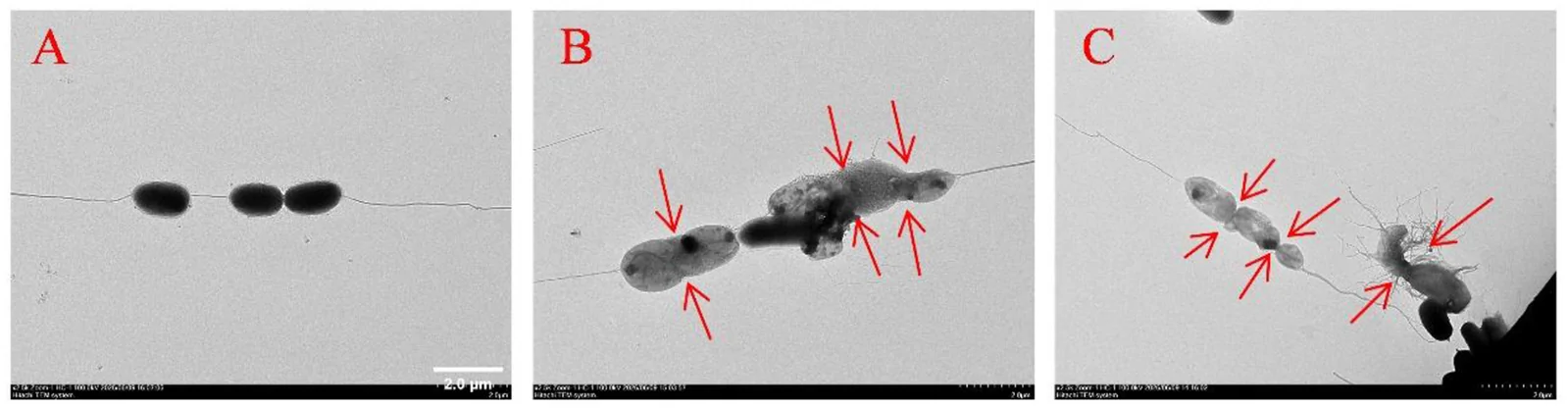
TEM images of *V. parahaemolyticus* treated with varying concentrations of the compound **1 (A)** Control (0 μg/mL); **(B)** MIC; **(C)** 2 × MIC. The scale bar of 2 μm is consistent for all images.

Transmission electron microscopy (TEM) was used to assess morphological changes in bacterial cells following compound treatment. Untreated control cells (0 μg/mL) displayed a typical short rod-shaped morphology, characterized by a smooth, continuous cell surface and a well-defined cell outline (Figure 3A). In contrast, cells treated with the compound at the MIC concentration exhibited obvious structural damage, including cell deformation, disruption of the cell membrane margin, and partial leakage of intracellular contents (Figure 3B). The damage was markedly more severe at 2 × MIC, with pronounced cell deformation, complete cell membrane lysis, and extensive leakage of cytoplasmic components (Figure 3C).

**Figure 4:**
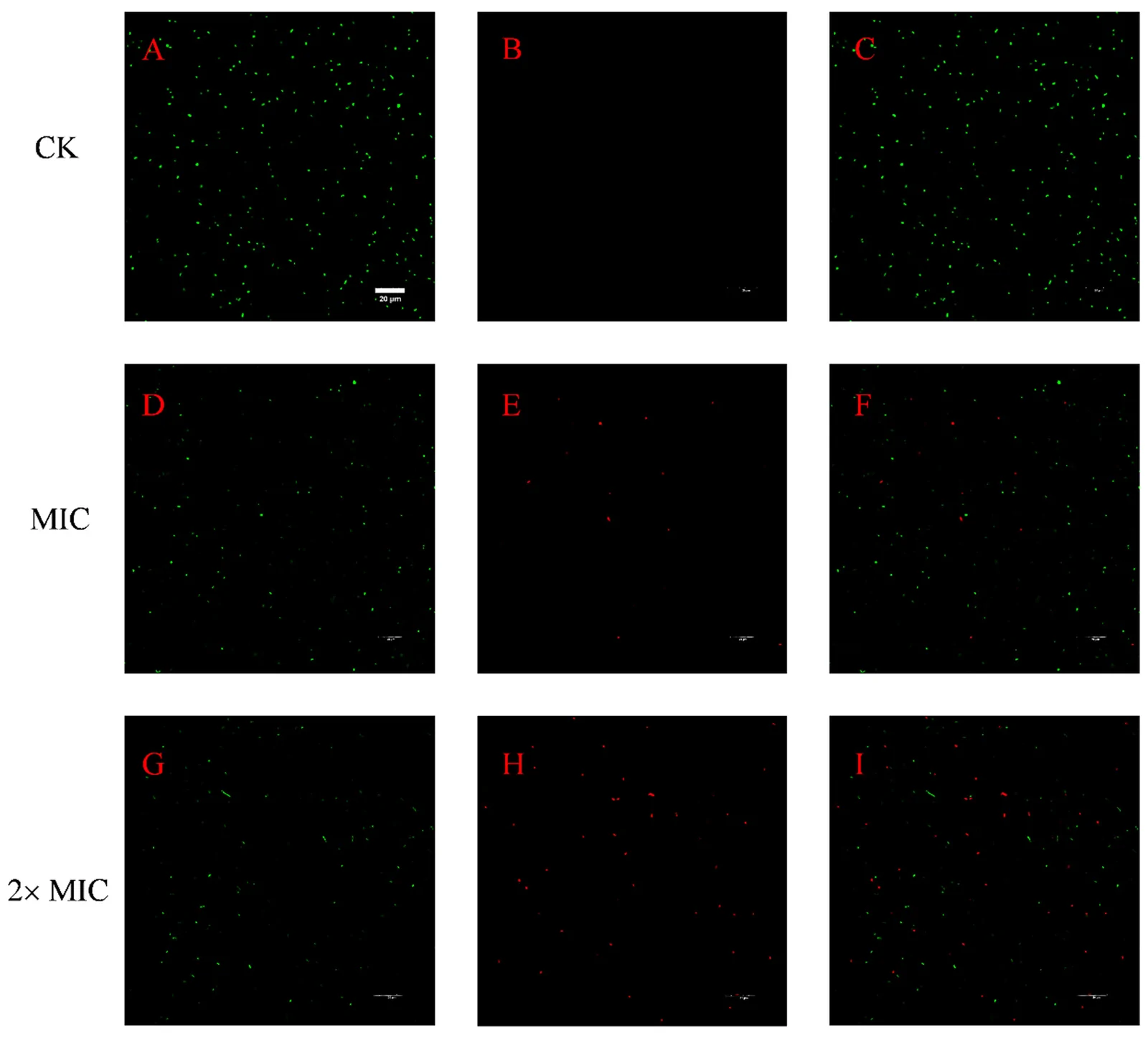
Detection of cell membrane integrity of *Vibrio parahaemolyticus* after treatment with different concentrations of the compound using SYTO 9/PI dual staining **(A, D, G)**: SYTO 9 staining (live bacteria, green fluorescence). **(B, E, H)**: PI staining (dead bacteria, red fluorescence). **(C, F, I)**: merged images of SYTO 9 and PI staining. CK: control group (0 μg/mL). MIC: minimum inhibitory concentration treatment group. 2 × MIC: 2 × minimum inhibitory concentration treatment group. The scale bar of 20 μm is consistent for all images.

**Figure 5:**
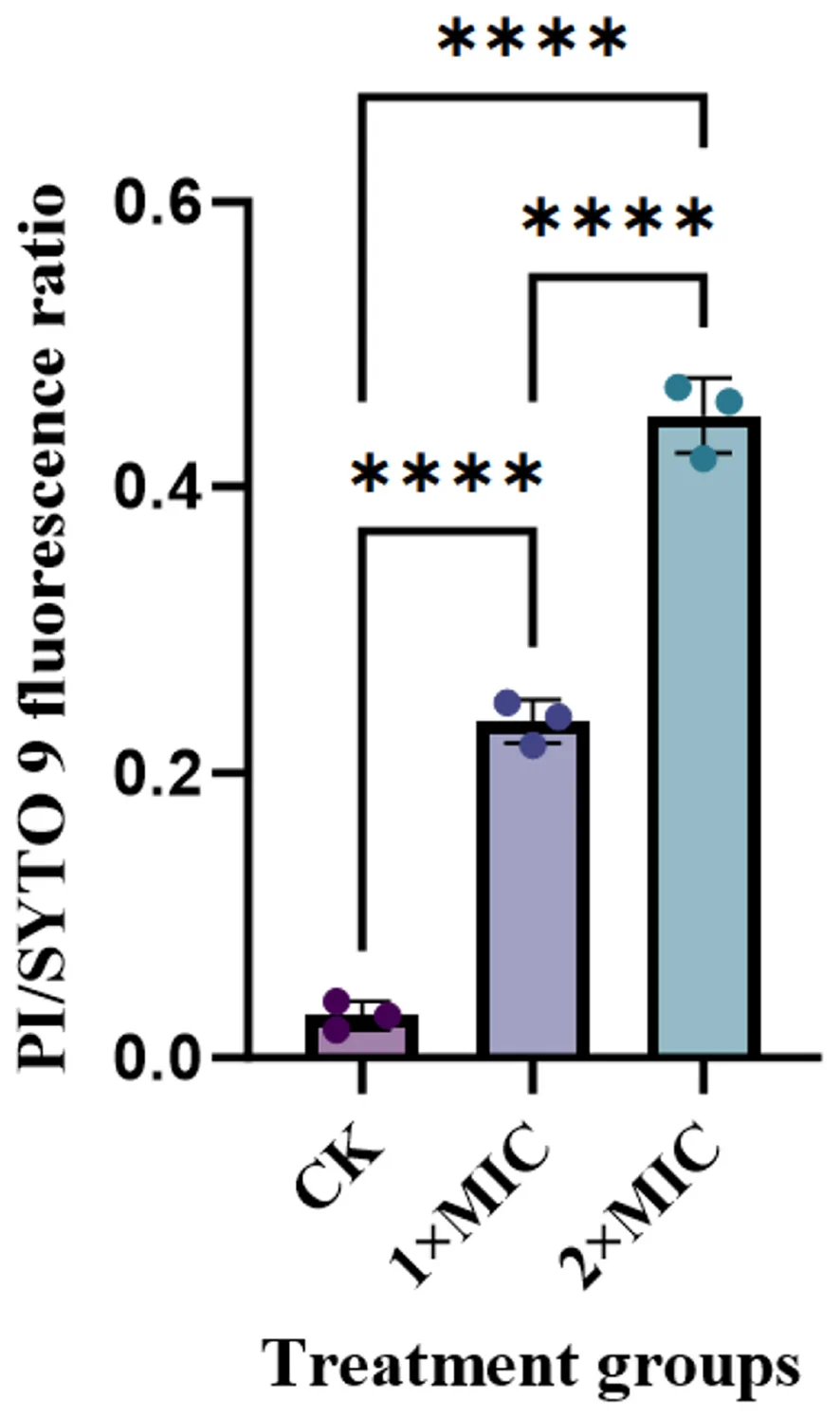
Quantification of the percentage of envelope-damaged Vibrio parahaemolyticus cells induced by penicillic acid via SYTO 9/PI double staining. The vertical axis value represents the proportion of PI-positive damaged bacterial cells. **** denotes extremely significant difference at *P* < 0.0001.

The effect of the compound on cell membrane integrity was further examined using confocal laser scanning microscopy following PI/SYTO 9 staining. Intact cells stained green, while those with damaged membranes-stained red. In the untreated control group, the majority of cells showed bright green fluorescence, with almost no detectable red signal (Figures 4A-C). Following MIC treatment, green fluorescence intensity was markedly reduced, accompanied by a clear increase in red fluorescence (Figures 4D-F). This trend intensified at 2 × MIC, where green fluorescence was further diminished and red fluorescence intensity exceeded that observed at MIC (Figures 4G-I). Quantitative analysis of penicillic acid-induced cell envelope damage in *Vibrio parahaemolyticus* was carried out using SYTO 9/PI double-stained CLSM images, and the PI/SYTO 9fluorescence ratio was used as the evaluation index. The average proportion of PI-positive damaged cells in the blank control group (CK) was only 0.03 ± 0.01. After treatment with penicillic acid at 1 × MIC, the percentage of damaged cells significantly increased to 0.24 ± 0.02, and the proportion increased further to 45.0 ± 3.0% at 2 × MIC (*p* < 0.001) (Figure 5). In summary, the percentage of PI-positive damaged cells increased in an obvious dose-dependent manner with the elevation of penicillic acid concentration, which confirms that penicillic acid exerts antibacterial activity by concentration-dependently destroying the cell envelope integrity of *V. parahaemolyticus*.

### 3.6 HaCaT cytotoxicity test

**Figure 6:**
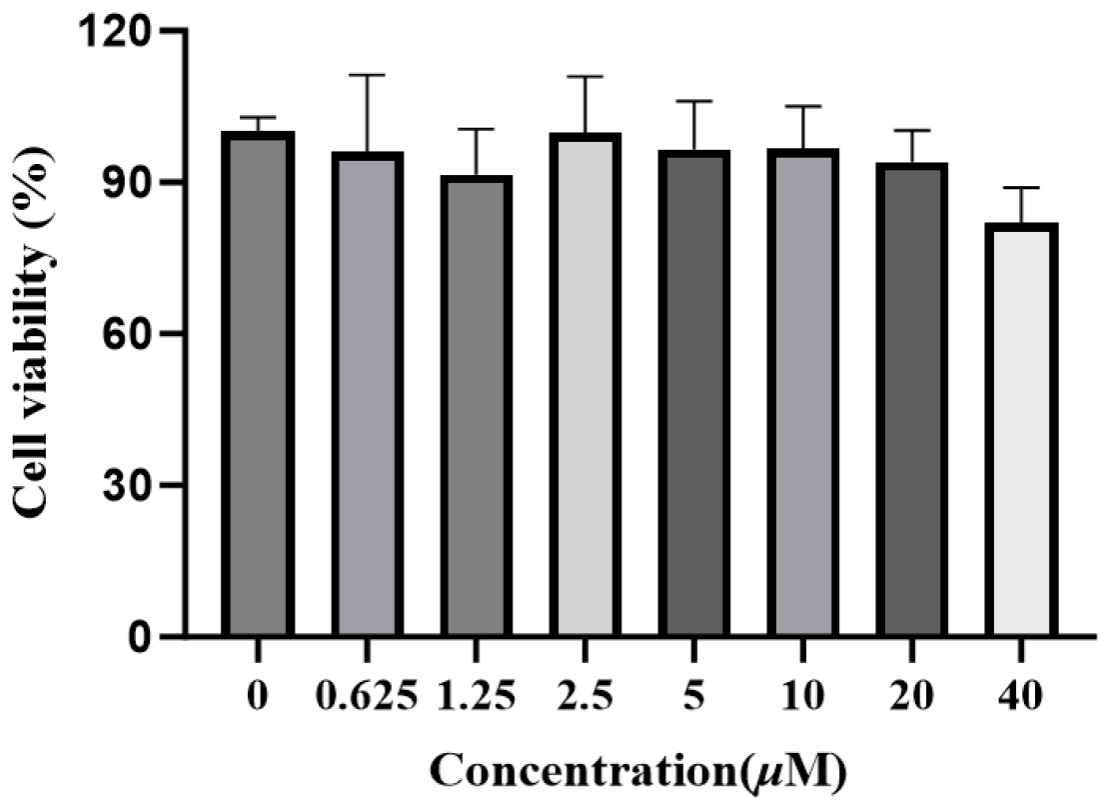
Cytotoxicity of the target compound against HaCaT keratinocytes detected by MTT assay.

The MTT assay was performed to evaluate the cytotoxicity of the target compound against human immortalized keratinocyte HaCaT cells. After continuous treatment with serially diluted compound concentrations ranging from 0.625 to 40 μM for 72 h, no obvious inhibitory effect on cell proliferation was observed in any treatment group. Even at the maximum tested concentration of 40 μM, the viability of HaCaT cells remained above 80% (Figure 6), with no statistically significant difference compared with the solvent control group (0 μM). Collectively, these results indicated that the compound exhibited very weak toxicity toward normal skin cells within the experimental concentration range and possessed favorable cellular safety. Therefore, the interference from cytotoxicity can be essentially excluded, providing a theoretical basis for subsequent *in vitro* antibacterial mechanistic studies.

## 4 Discussion and conclusions

Mangrove endophytic fungi are regarded as valuable reservoirs of bioactive secondary metabolites (Wang et al., 2022; Liu et al., 2026). In this study, a total of 19 culturable endophytic fungal strains were isolated from mangrove tissues, among which *Aspergillus* and *Penicillium* were the dominant genera, accounting for 42.1% of all isolates. Antibacterial screening revealed that strain BM19 (tentatively identified as *Aspergillus sclerotiorum*) exhibited prominent broad-spectrum inhibitory activity against three aquatic pathogenic *vibrios*, including *V. parahaemolyticus, V. alginolyticus*, and *V. owensii*. Its crude extract showed superior antibacterial potency compared with other isolates, which is consistent with the consensus that *Aspergillus* fungi serve as important sources of anti-*Vibrio* metabolites (Huang et al., 2022).

Penicillic acid (**1**, 286.5 mg) was obtained from the ethyl acetate extract of strain BM19 via bioactivity-guided isolation. MIC determination verified that penicillic acid exerted potent anti-*Vibrio* activity, with MIC values of 6.25, 12.5, and 25 μg/mL against *V. parahaemolyticus, V. owensii*, and *V. alginolyticus*, respectively. Comparative analysis with previously reported marine-derived anti-*Vibrio* metabolites demonstrated that penicillic acid displayed stronger activity than versiconol B from sponge-associated *Aspergillus* sp. F40 (MIC = 12.0 μg/mL against *V*. *parahaemolyticus*) (Tian et al., 2018) and comparable efficacy to enidentonin C from deep-sea-derived *Aspergillus versicolor* SD-330 (MIC = 4.0–8.0 μg/mL) (Li et al., 2019). In contrast to compounds from marine *Streptomyces* sp. ZZ741A (MIC = 8–128 μg/mL) (Zhang et al., 2025), penicillic acid exhibited advantages in both inhibitory potency and antibacterial spectrum. It also showed equivalent activity to phomones A–C from sponge symbiotic *Phoma* sp. NBUF235 (MIC = 8–32 μg/mL) (Lai et al., 2026); however, the antibacterial mechanism of phomones has not yet been characterized, whereas the present study provides morphological evidence of membrane disruption for penicillic acid. As a small-molecule compound, penicillic acid offers advantages in chemical stability, scalability, and cost-effectiveness compared with antimicrobial peptides such as Larimicin78–102 and Ajapocin (Zhang et al., 2025; Wang et al., 2026). its inherent mycotoxin properties necessitate rigorous biosafety evaluation prior to any practical application in aquaculture. Growth curve analysis further confirmed a dose-dependent inhibitory pattern: 0.5 × MIC delayed the logarithmic growth phase of *V. parahaemolyticus*, while 1 × MIC and 2 × MIC completely suppressed bacterial proliferation.

Transmission electron microscopy (TEM) and SYTO 9/PI double-staining confocal laser scanning microscopy (CLSM) revealed dose-dependent bacterial morphological damage and impaired membrane integrity induced by penicillic acid. Treatment at the MIC level caused cellular deformation, blurred membrane boundaries, and partial cytoplasmic leakage, while 2 × MIC treatment resulted in severe membrane rupture and massive efflux of intracellular contents. Quantitative CLSM analysis further confirmed a concentration-dependent trend, with gradually attenuated green fluorescence and enhanced red fluorescence as the compound dosage increased. This unique cell-envelope destructive phenotype distinguishes penicillic acid from previously reported anti-*Vibrio* candidates. For example, nocardamine and dehydroxynocardamine derived from marine streptomycetes are speculated to target the outer membrane protein OmpU (Zhang et al., 2025), while the antibacterial mechanism of phomones remains unreported (Lai et al., 2026). Although the antimicrobial peptide Ajapocin also induces membrane depolarization (Wang et al., 2026), the phenotypic alterations differ substantially from the extensive structural disruption observed in penicillic acid-treated cells. Collectively, these findings indicate that penicillic acid treatment is closely associated with cell-envelope damage and increased membrane permeability in *V. parahaemolyticus*.

The bacterial cell envelope is a crucial and druggable target for antimicrobial agents (Epand et al., 2016). Unlike conventional antibiotics that interfere with intracellular biosynthetic pathways, membrane-targeting compounds generally act rapidly with a lower risk of antimicrobial resistance development (Hurdle et al., 2011). In the present study, obvious cell-envelope damage was observed within 2 h of penicillic acid exposure, which aligns with the rapid-acting characteristics of membrane-interfering antimicrobials. Previous studies have reported that penicillic acid exhibits only weak antibacterial activity against terrestrial pathogens, including *Staphylococcus aureus, MRSA*, and *Escherichia coli*, with MIC values ranging from 128 to 200 μg/mL (Phainuphong et al., 2017; Rukachaisirikul et al., 2019). In comparison, its remarkably low MIC value (6.25 μg/mL) against aquatic *vibrios* indicates a preferential inhibitory tendency toward specific Gram-negative aquatic pathogens, which deserves further exploration in future studies.

Although TEM and CLSM clearly visualized membrane-damaging phenotypes, morphological evidence alone cannot distinguish direct compound–membrane interactions from secondary membrane injury triggered by intracellular electrophilic or oxidative stress. Given that penicillic acid is an electrophilic mycotoxin, such secondary stress-induced effects constitute a reasonable alternative mechanistic explanation. The supplemented MTT assay using human HaCaT keratinocytes showed that penicillic acid exerted negligible eukaryotic cytotoxicity within the effective antibacterial concentration range (0.625–40 μM), with cell viability remaining above 80% at the maximum tested concentration. These results suggest that non-specific electrophilic toxicity is unlikely to be the major cause of the observed membrane damage. However, definitive validation of a primary membrane-targeting mechanism requires further biophysical evidence, including liposome leakage assays, zeta potential detection, and DiSC3(5)-based membrane depolarization analysis.

In conclusion, this study isolated penicillic acid from the mangrove endophytic fungus *Aspergillus sclerotiorum* BM19 and verified its broad-spectrum, dose-dependent bacteriostatic activity against multiple pathogenic *Vibrio* species, with morphological evidence demonstrating its cell-envelope-disrupting effects. Penicillic acid exhibits competitive anti-*Vibrio* potency relative to most reported marine-derived small-molecule antimicrobials, and its small-molecule nature confers superior chemical stability and scalable production potential. Future research will focus on biophysical verification of direct compound–membrane interactions, systematic elucidation of the underlying molecular mechanisms, and comprehensive evaluation of in vivo efficacy and biosafety.

## Data Availability

The datasets presented in this study can be found in online repositories. The names of the repository/repositories and accession number(s) can be found in the article/Supplementary material.
