# Supplementary material for: Discovery of penicillic acid from mangrove endophytic fungi: anti-Vibrio screening and cell envelope disruption in V. parahaemolyticus
Source: Front Microbiol. 2026 Aug 31;17:1939258. doi: 10.3389/fmicb.2026.1939258 (PMC13548119; doi:10.3389/fmicb.2026.1939258)
Supplement: Supplementary file 1 [file Data_Sheet_1.pdf]

Supporting information

Diversity, chemical composition and anti-Vibrio activity of endophytic fungi from the mangrove plants

Yaqi Chen[1,2,†], Fan An[1,2,†], Yuying Chen[1,2], Ziyi Huang[1,2], Caixia Hou[1,2], Xiangxi Yi[1,2,3,4], Chenghai Gao[1,2,3,4, *], Yuman Gan[1,2,3,4, *], and Meng Bai[1,2,3,4,*1]

[1] Guangxi Key Laboratory of Marine Drugs, Guangxi University of Chinese Medicine, Nanning, 530200, P.R. China;

[2] University Engineering Research Center of High-efficient Utilization of Marine Traditional Chinese Medicine Resources, Nanning, 530200, P.R. China;

[3] Technology Innovation Center for the Development and Utilization of Marine Drugs and Bioproducts for Beibu Gulf, MNR, Nanning, 530200, P.R. China;

[4] Institute of Marine Drugs, Guangxi University of Chinese Medicine, Nanning, 530200, P.R. China;

† These authors contributed equally to this work.

[1] Correspondence: gaochh@gxtcmu.edu.cn (C.-H. Gao); gan_ym2018@163.com (Y.-M. Gan); XXBai2014@163.com (M. Bai).

List of contents

Figure S1. ^{1}H NMR (400 MHz) spectrum of **1** in DMSO-d_6
Figure S2. ^{13}C NMR (100 MHz) spectrum of **1** in DMSO-d_6
Figure S3. 135º-DEPT spectrum of **1** in DMSO-d_6
Figure S4. HSQC spectrum of **1** in DMSO-d_6
Figure S5. ^{1}H-^{1}H COSY spectrum of **1** in DMSO-d_6
Figure S6. HMBC spectrum of **1** in DMSO-d_6
Figure S7. NOESY spectrum of **1** in DMSO-d_6
Figure S8. HR-ESI-MS spectrum of **1**

Figure S9. ^{1}H NMR (400 MHz) spectrum of **2** in DMSO-d_6
Figure S10. ^{13}C NMR (100 MHz) spectrum of **2** in DMSO-d_6
Figure S11. 135º-DEPT spectrum of **2** in DMSO-d_6
Figure S12. HSQC spectrum of **2** in DMSO-d_6
Figure S13. ^{1}H-^{1}H COSY spectrum of **2** in DMSO-d_6
Figure S14. HMBC spectrum of **2** in DMSO-d_6
Figure S15. NOESY spectrum of **2** in DMSO-d_6
Figure S16. HR-ESI-MS spectrum of **2**

Figure S17. ^{1}H NMR (400 MHz) spectrum of **3** in DMSO-d_6
Figure S18. ^{13}C NMR (100 MHz) spectrum of **3** in DMSO-d_6
Figure S19. 135º-DEPT spectrum of **3** in DMSO-d_6
Figure S20. HSQC spectrum of **3** in DMSO-d_6
Figure S21. ^{1}H-^{1}H COSY spectrum of **3** in DMSO-d_6
Figure S22. HMBC spectrum of **3** in DMSO-d_6
Figure S23. NOESY spectrum of **3** in DMSO-d_6
Figure S24. HR-ESI-MS spectrum of **3**

Figure S25. ^{1}H NMR (400 MHz) spectrum of **4** in DMSO-d_6
Figure S26. ^{13}C NMR (100 MHz) spectrum of **4** in DMSO-d_6
Figure S27. 135º-DEPT spectrum of **4** in DMSO-d_6
Figure S28. HSQC spectrum of **4** in DMSO-d_6
Figure S29. ^{1}H-^{1}H COSY spectrum of **4** in DMSO-d_6
Figure S30. HMBC spectrum of **4** in DMSO-d_6
Figure S31. NOESY spectrum of **4** in DMSO-d_6
Figure S32. HR-ESI-MS spectrum of **4**

Figure S33. ^{1}H NMR (400 MHz) spectrum of **5** in DMSO-d_6
Figure S34. ^{13}C NMR (100 MHz) spectrum of **5** in DMSO-d_6
Figure S35. 135º-DEPT spectrum of **5** in DMSO-d_6
Figure S36. HSQC spectrum of **5** in DMSO-d_6

Figure S37. ^1H-^1H COSY spectrum of **5** in DMSO-d_6
Figure S38. HSBC spectrum of **5** in DMSO-d_6
Figure S39. NOESY spectrum of **5** in DMSO-d_6
Figure S40. HR-ESI-MS spectrum of **5**
Figure S41. Colony morphologies of fungal strains BM1 to BM19 cultured on PDA medium.
Figure S42. Microscopic micrographs of fungal strains BM1–BM19.
Figure S43. Inhibitory activities of crude extracts of BM1–BM9 against *Vibrio*.
Figure S44. Growth curves of *V. parahaemolyticus* treated by compounds **1**.
ITS sequences of strains BM1-19

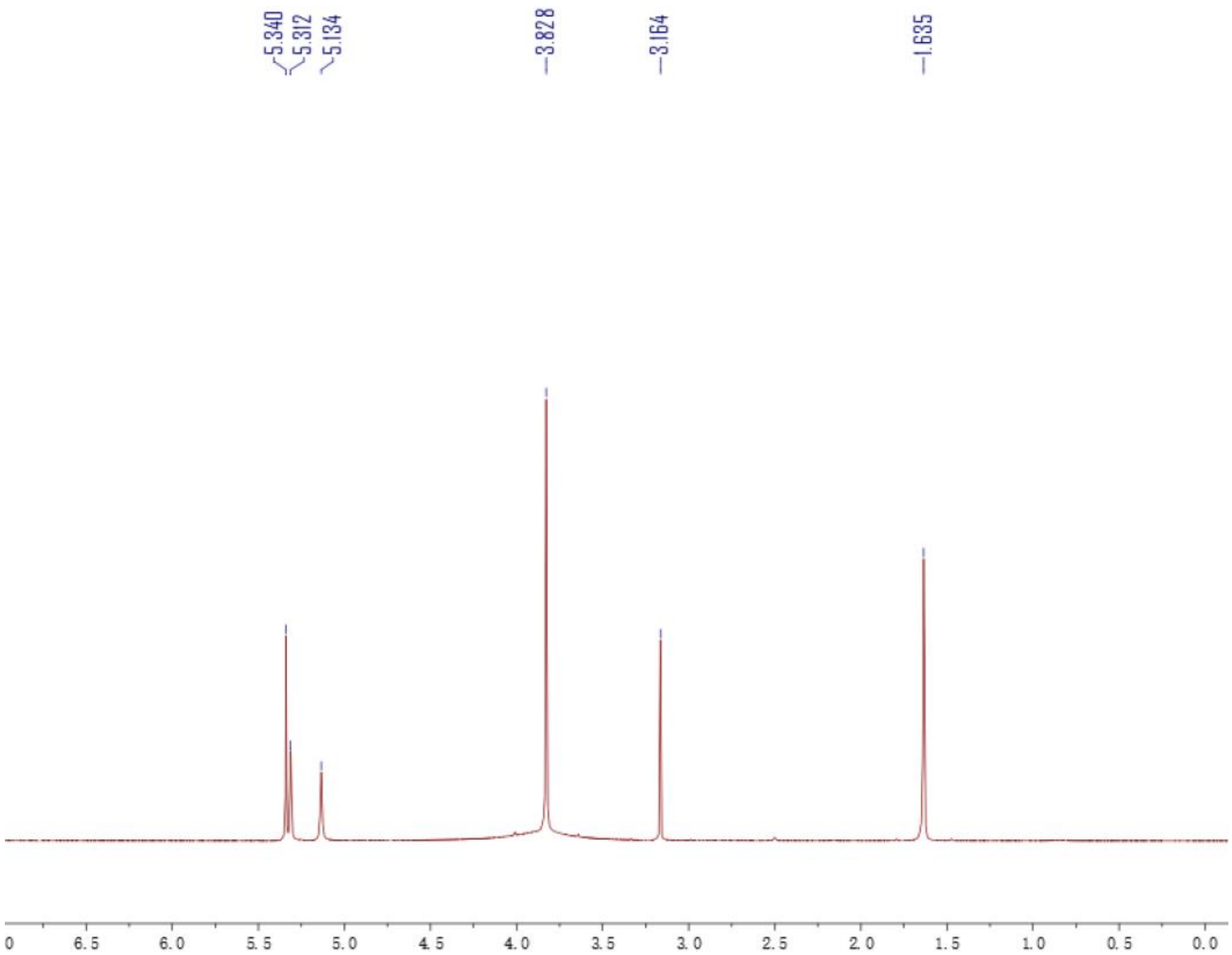

Figure S1. ^{1}H NMR (400 MHz) spectrum of **1** in DMSO-d_6

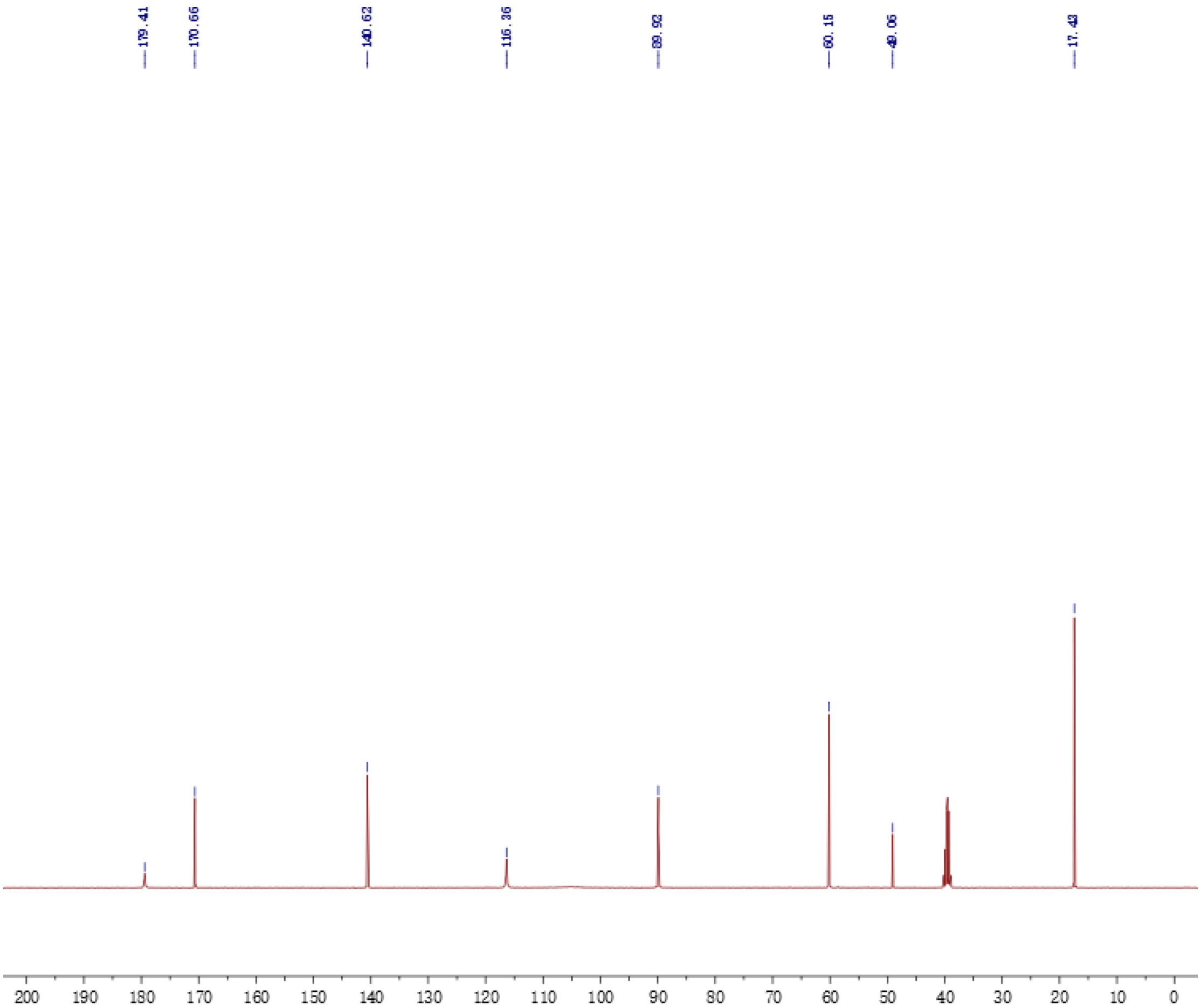

Figure S2. ^{13}C NMR (100 MHz) spectrum of **1** in DMSO-d_6

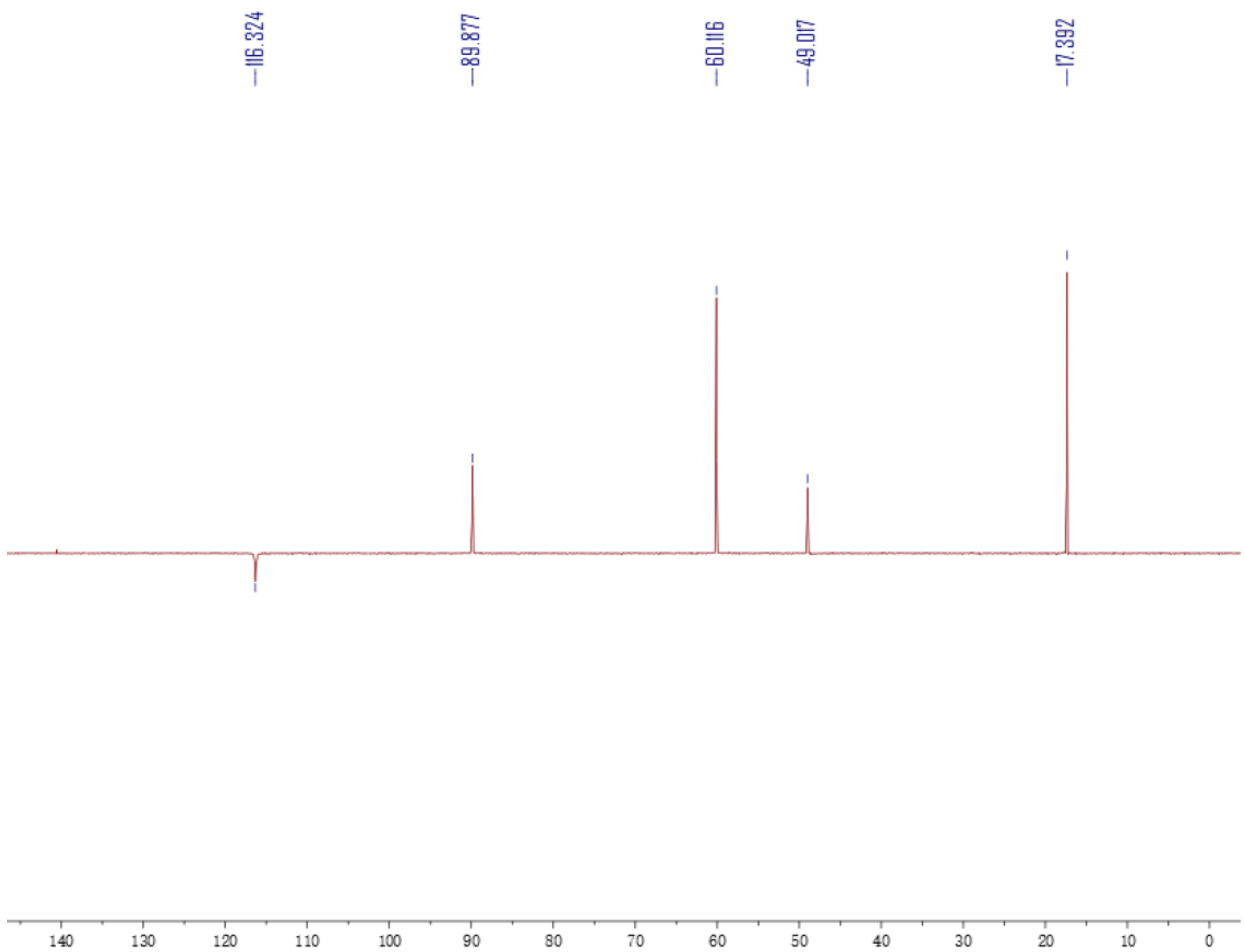

Figure S3. 135°-DEPT spectrum of **1** in DMSO-d_6

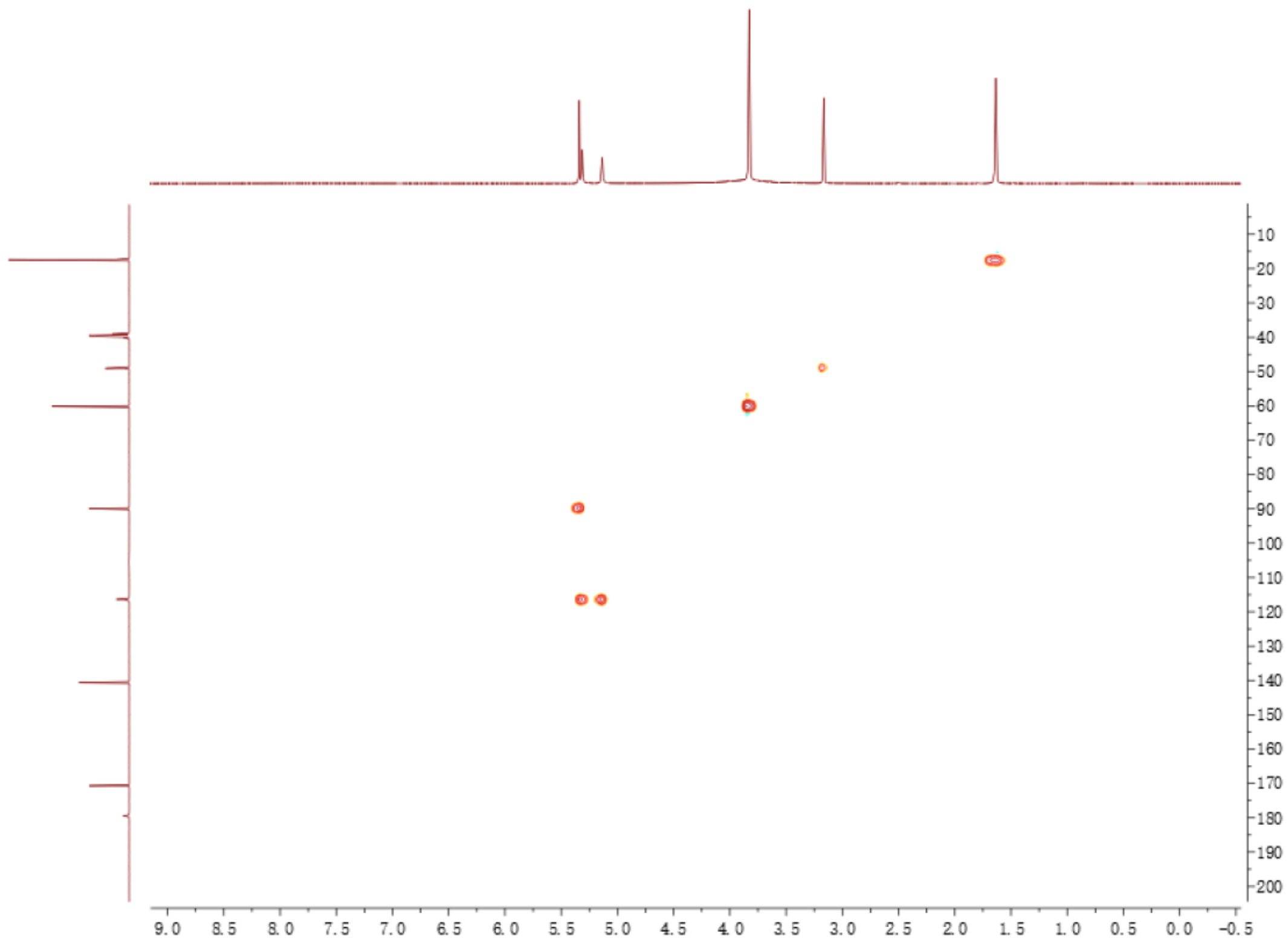

Figure S4. HSQC spectrum of **1** in DMSO-d_6

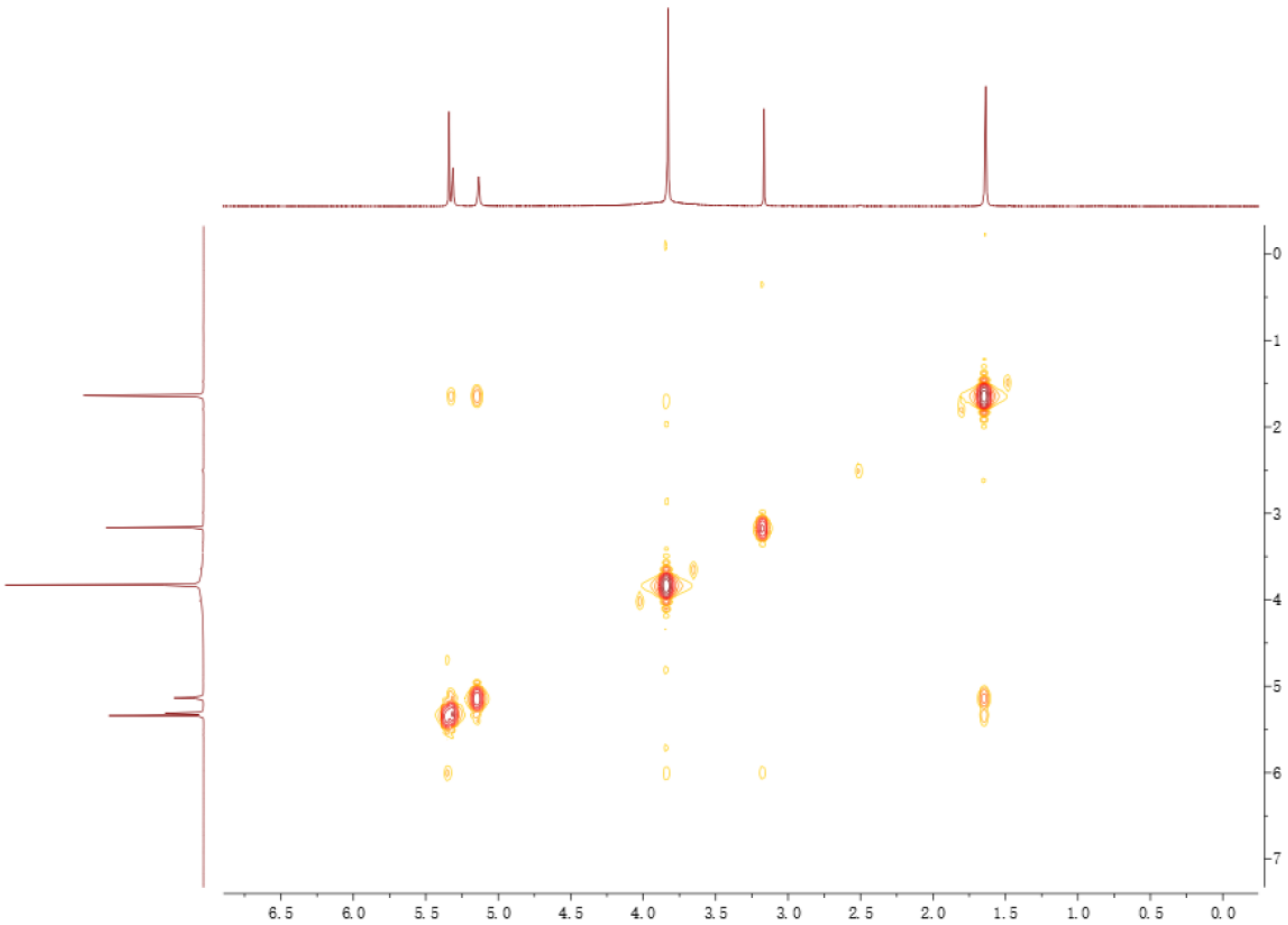

Figure S5. ^{1}H-^{1}H COSY spectrum of **1** in DMSO-d_6

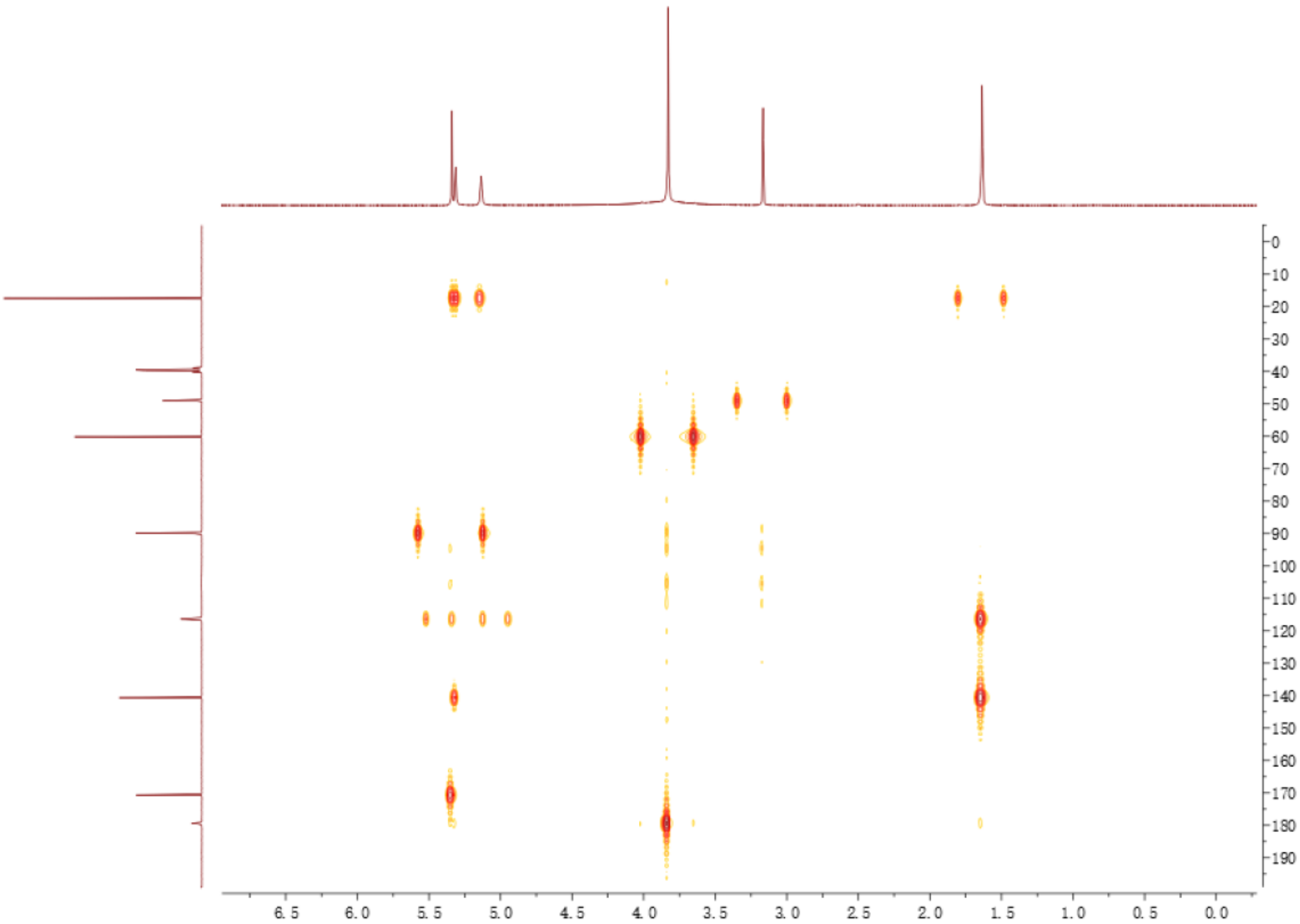

Figure S6. HMBC spectrum of **1** in DMSO-d_6

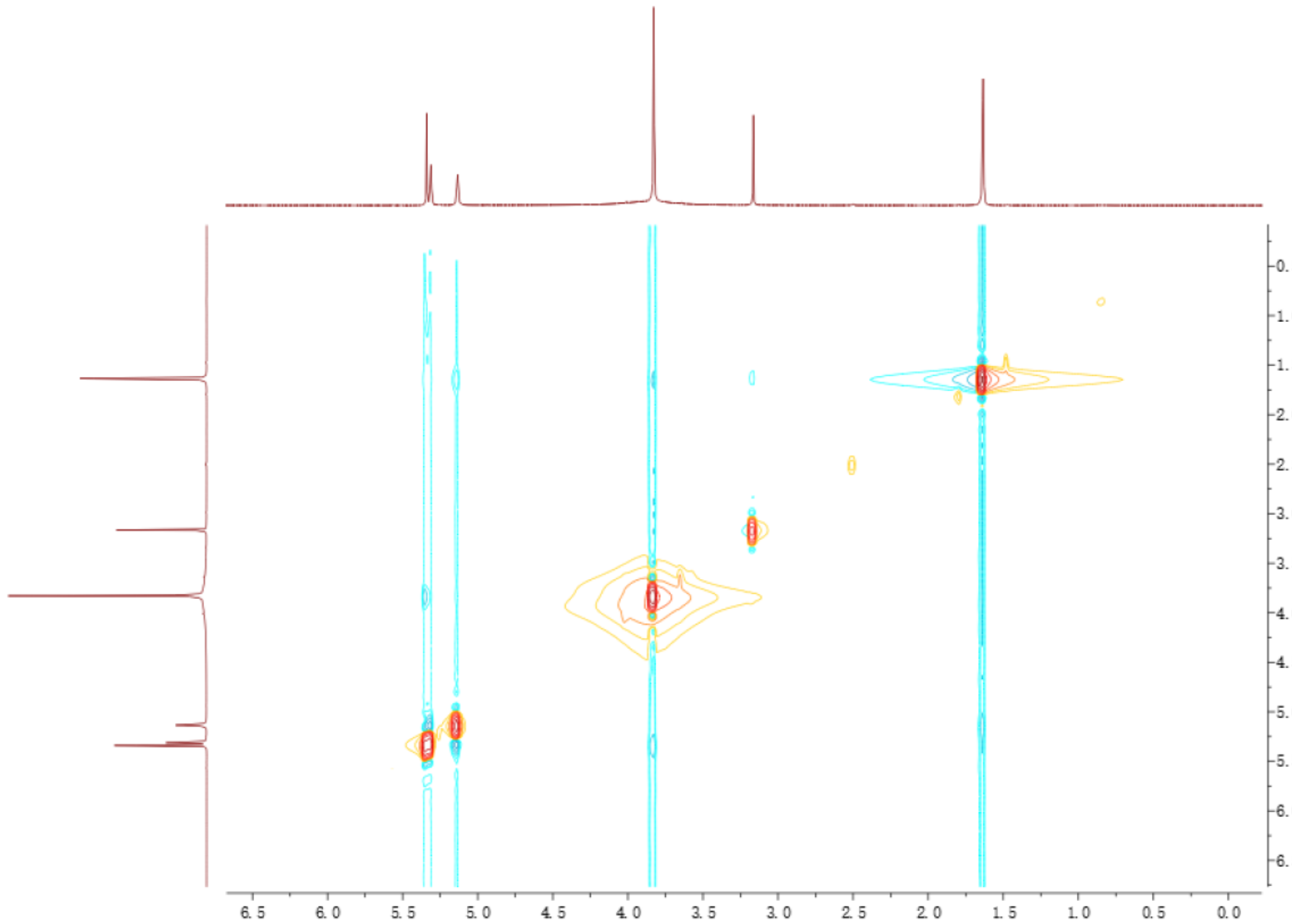

Figure S7. NOESY spectrum of **1** in DMSO-d_6

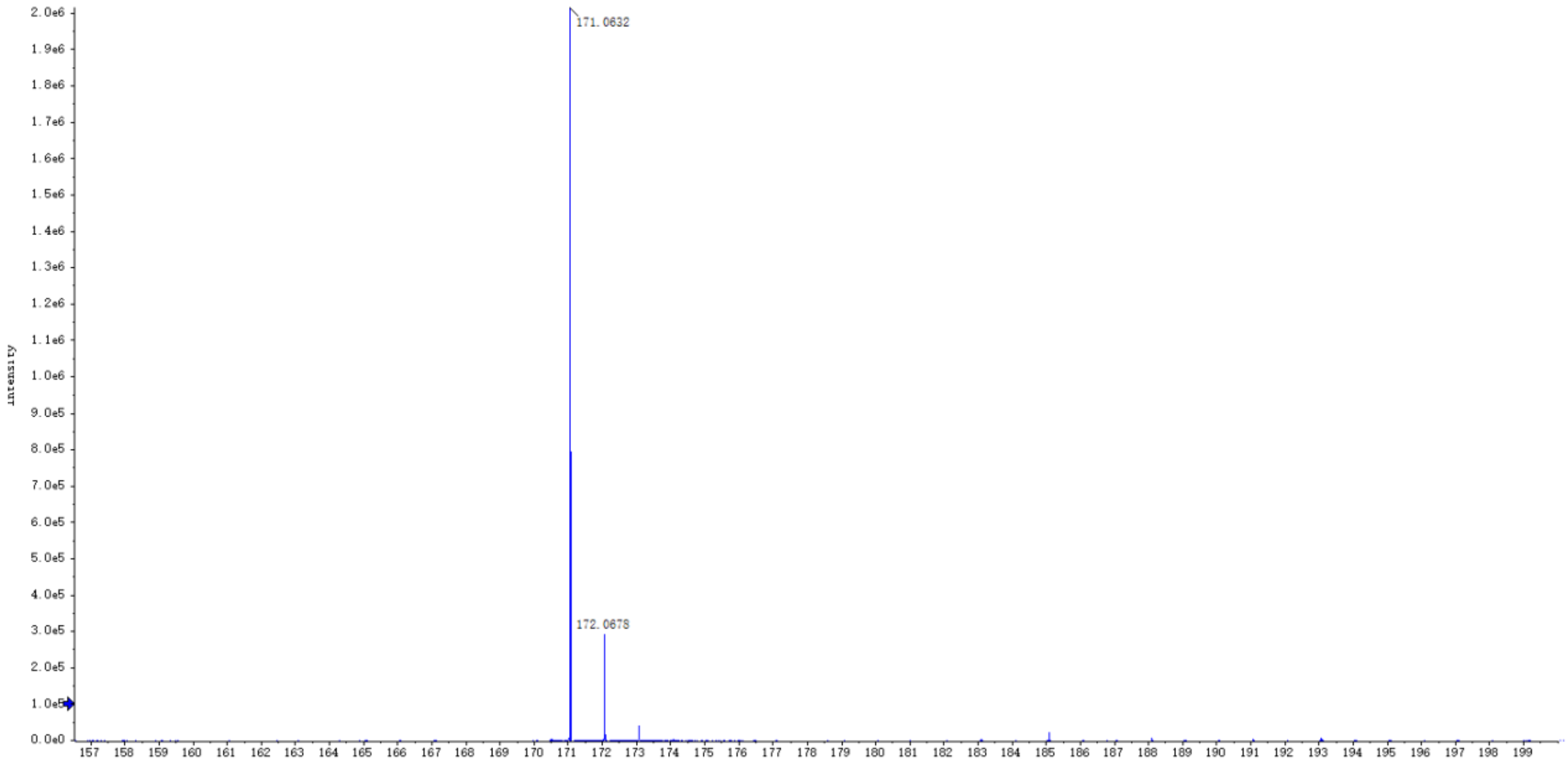

Figure S8. HR-ESI-MS spectrum of **1**

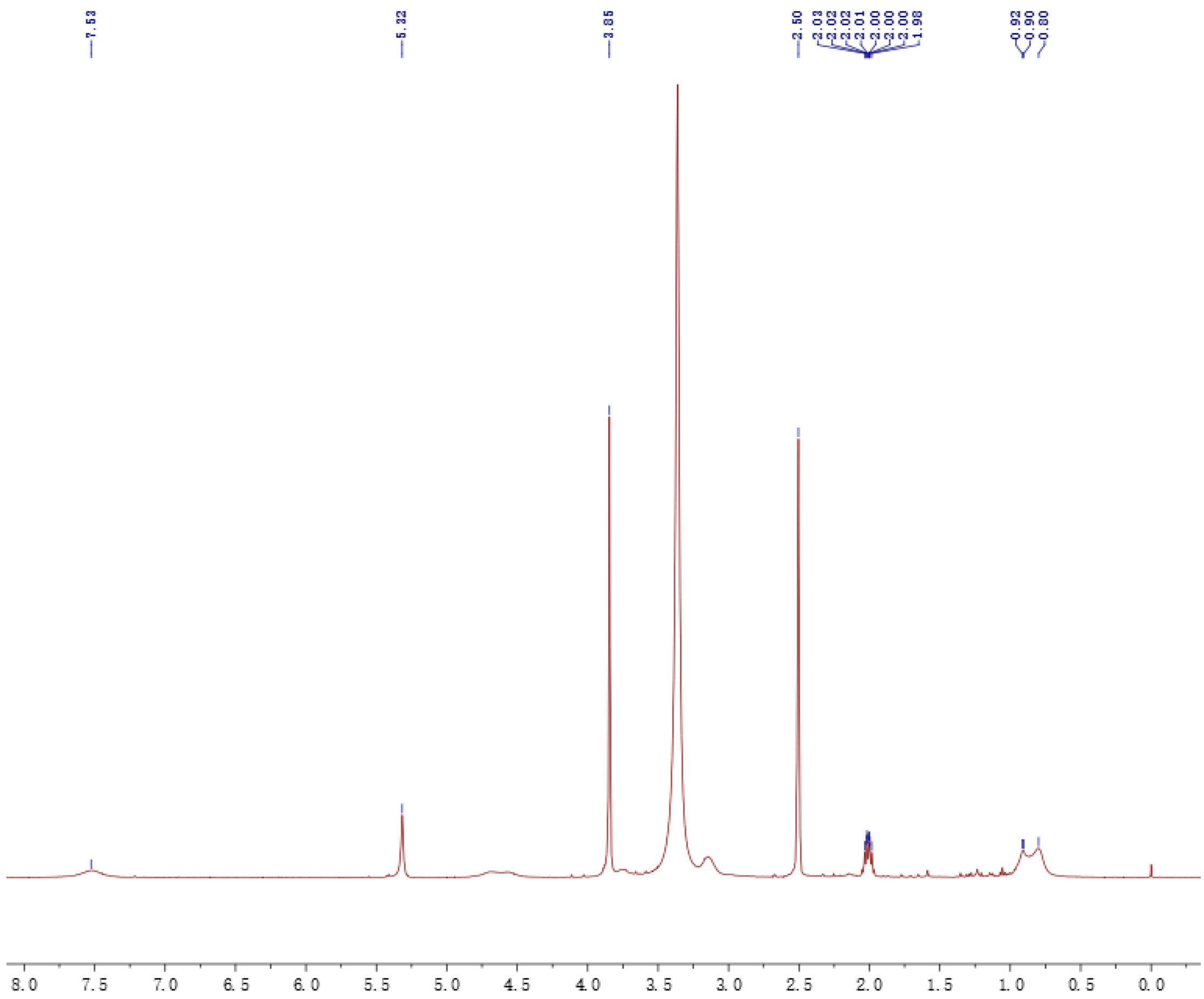

Figure S9. ^{1}H NMR (400 MHz) spectrum of **2** in DMSO-d_6

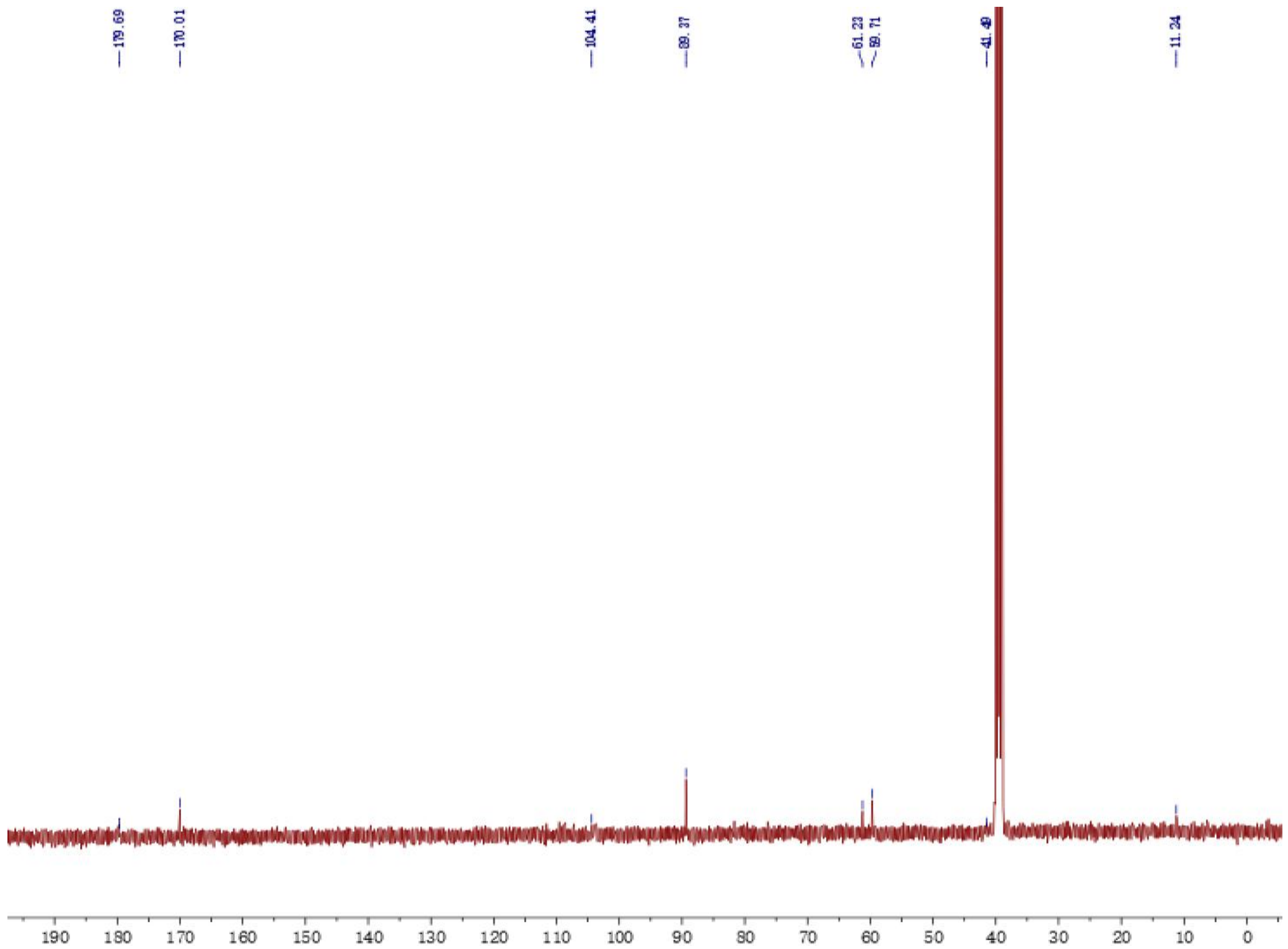

Figure S10. ^{13}C NMR (100 MHz) spectrum of **2** in DMSO-d_6

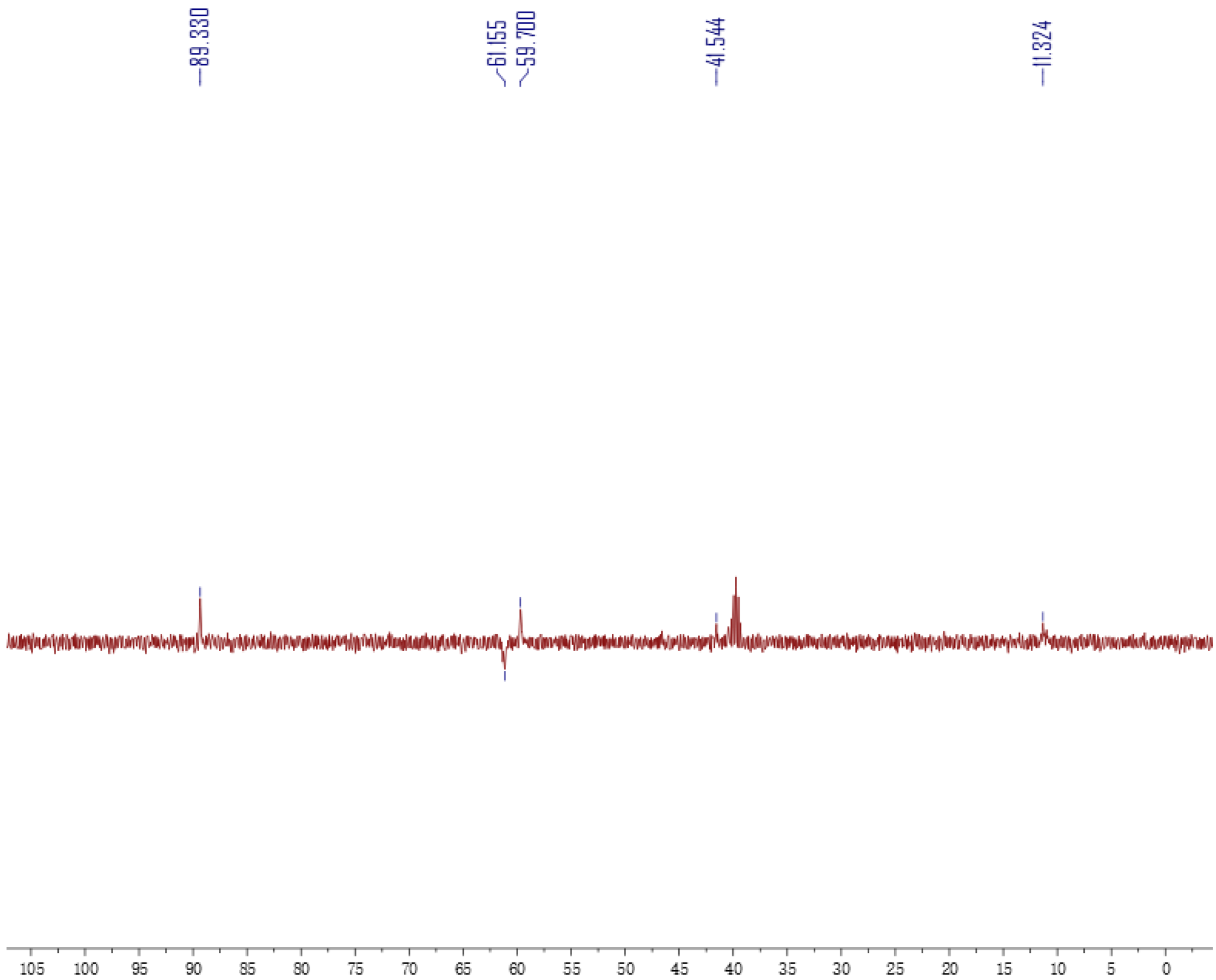

Figure S11. 135°-DEPT spectrum of **2** in DMSO-d_6

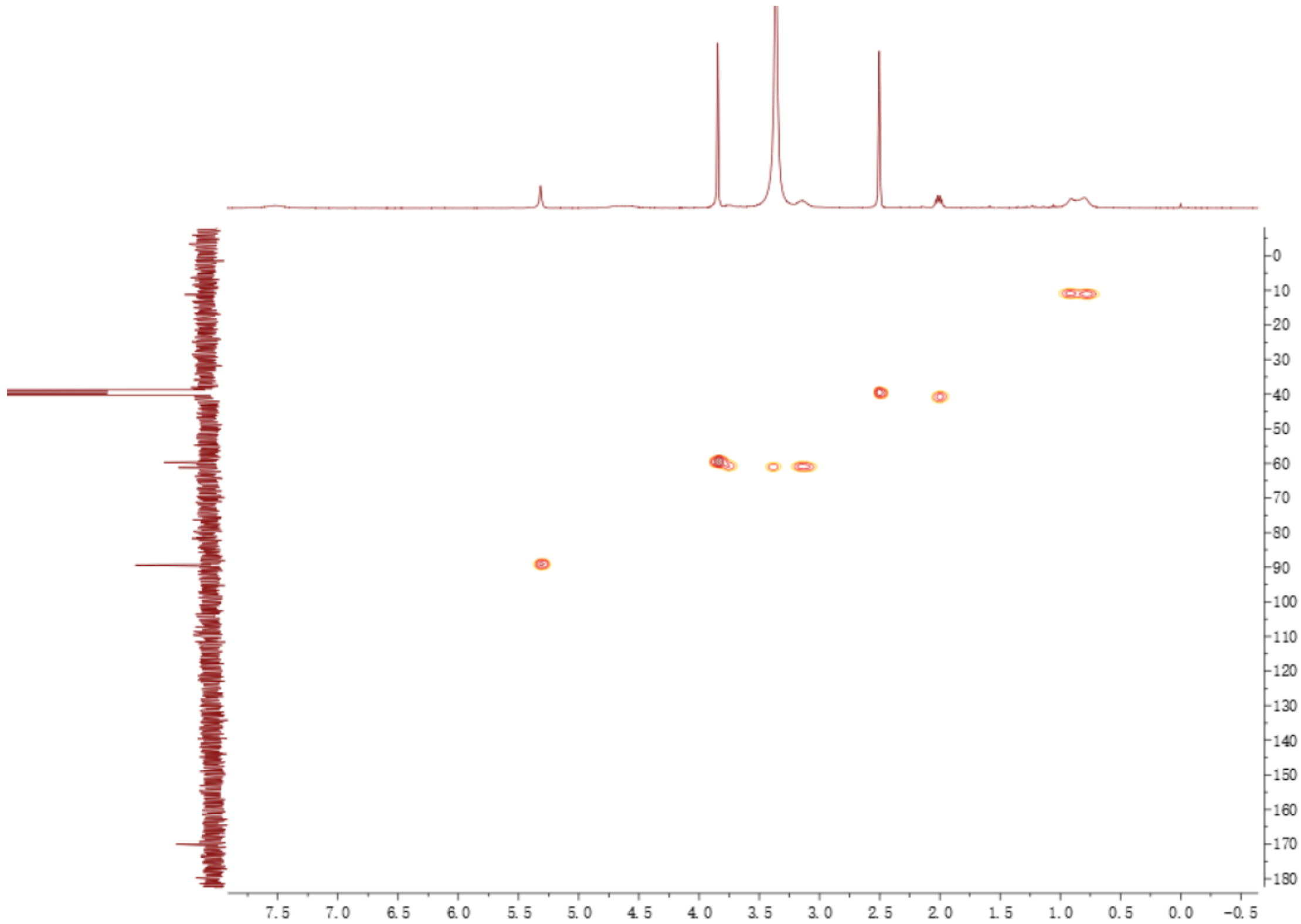

Figure S12. HSQC spectrum of **2** in DMSO-d_6

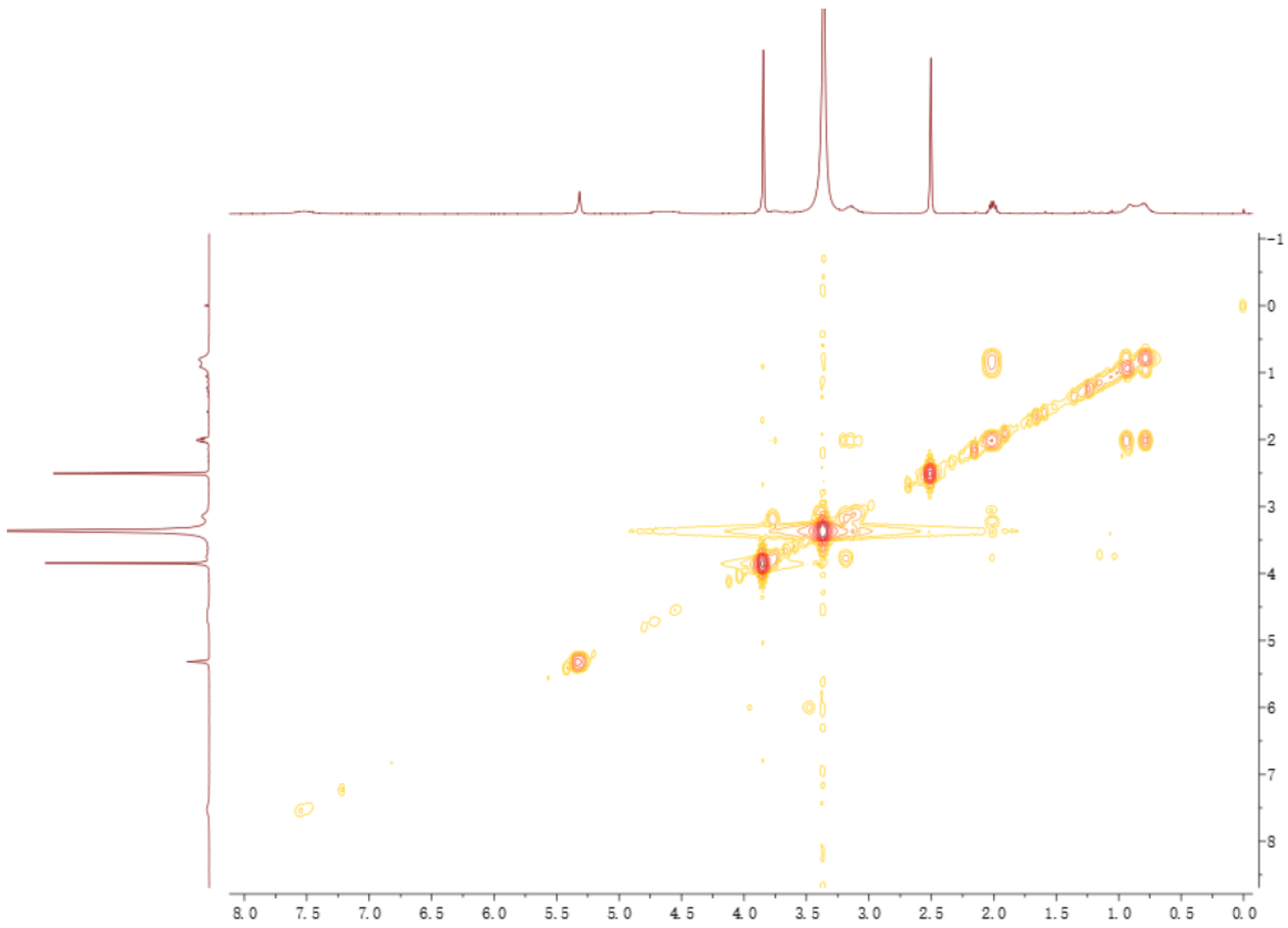

Figure S13. ^{1}H-^{1}H COSY spectrum of **2** in DMSO-d_6

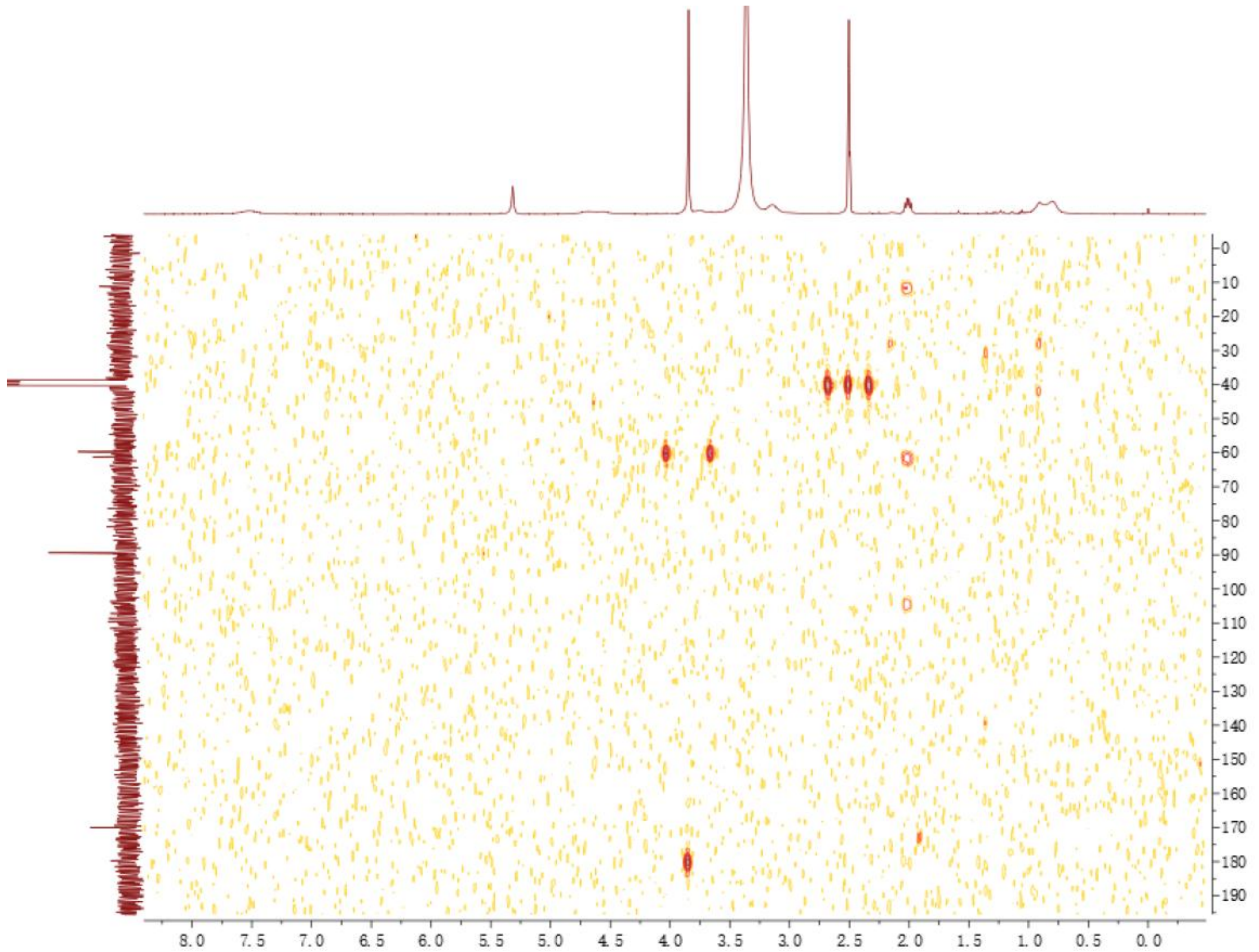

Figure S14. HMBC spectrum of **2** in DMSO-d_6

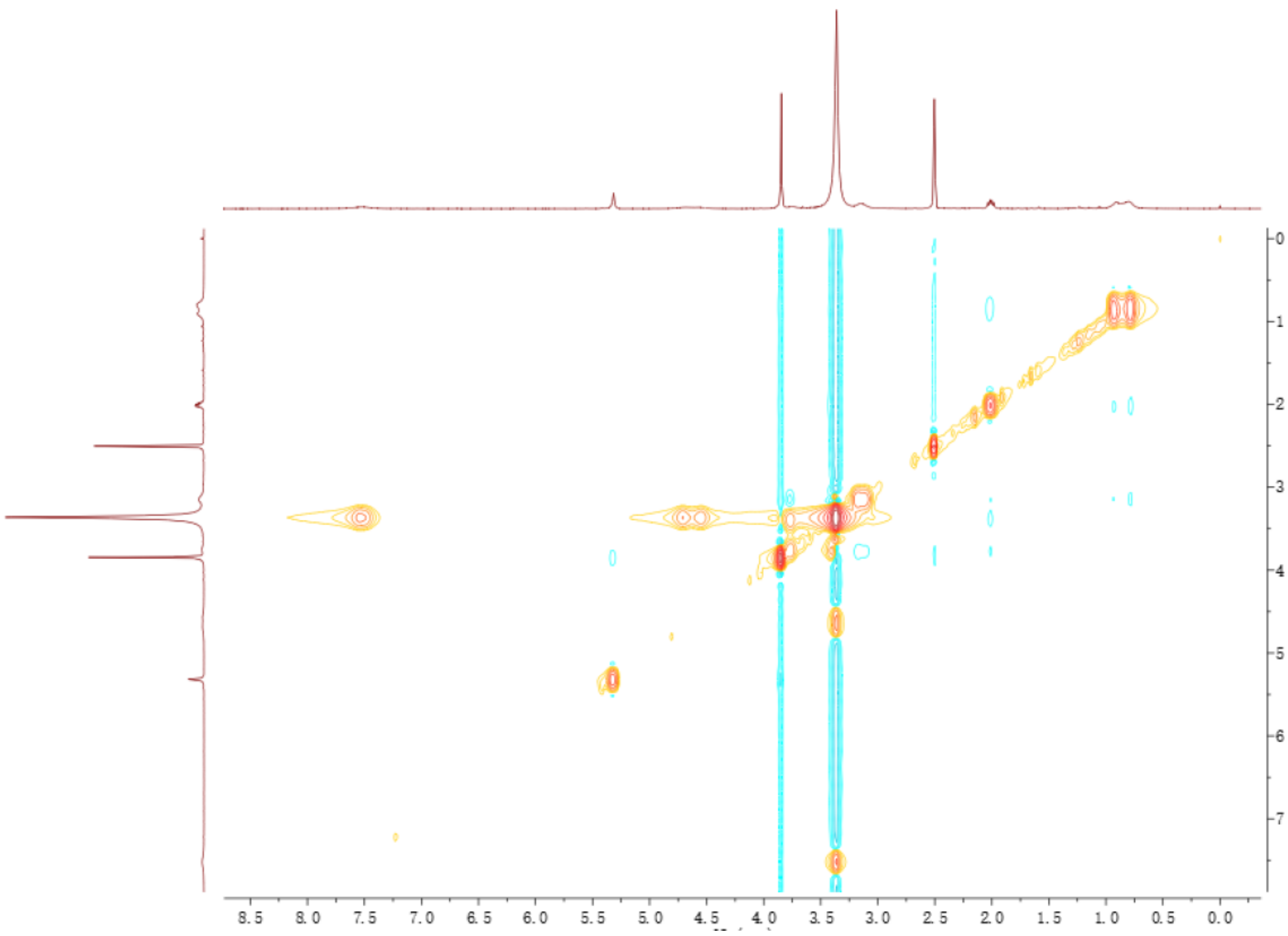

Figure S15. NOESY spectrum of **2** in DMSO-d_6

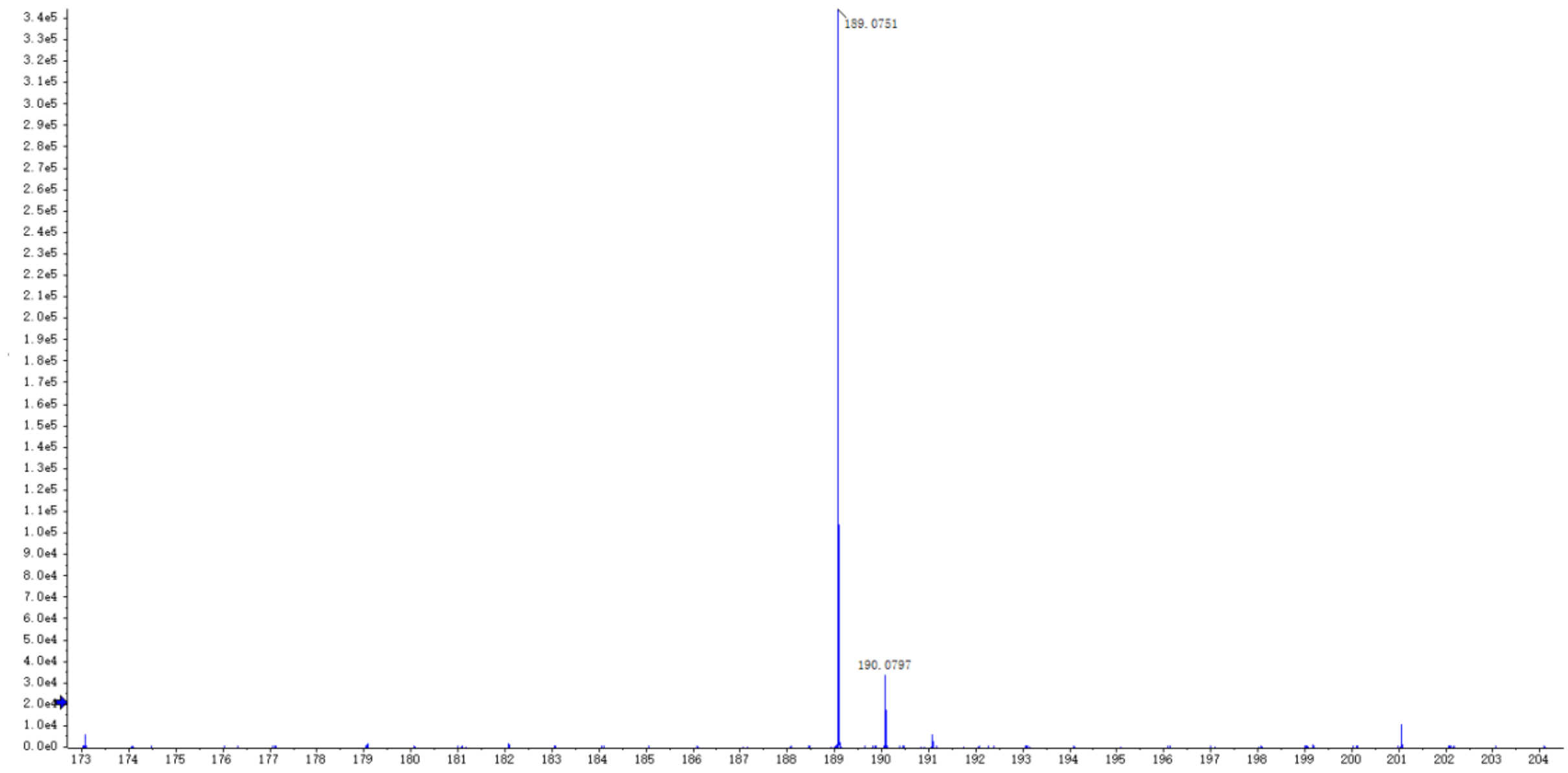

Figure S16. HR-ESI-MS spectrum of **2**

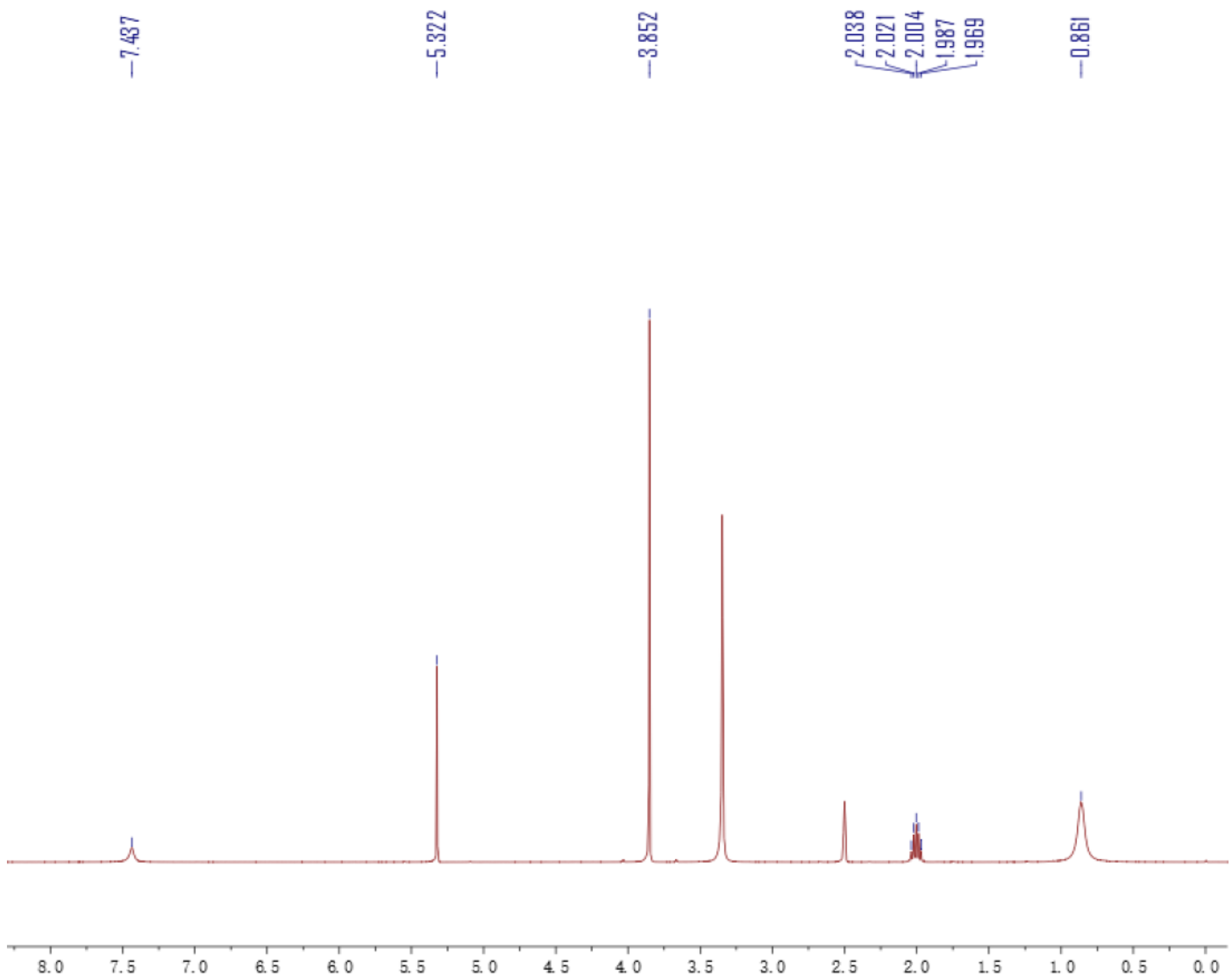

Figure S17. ^{1}H NMR (400 MHz) spectrum of **3** in DMSO-d_6

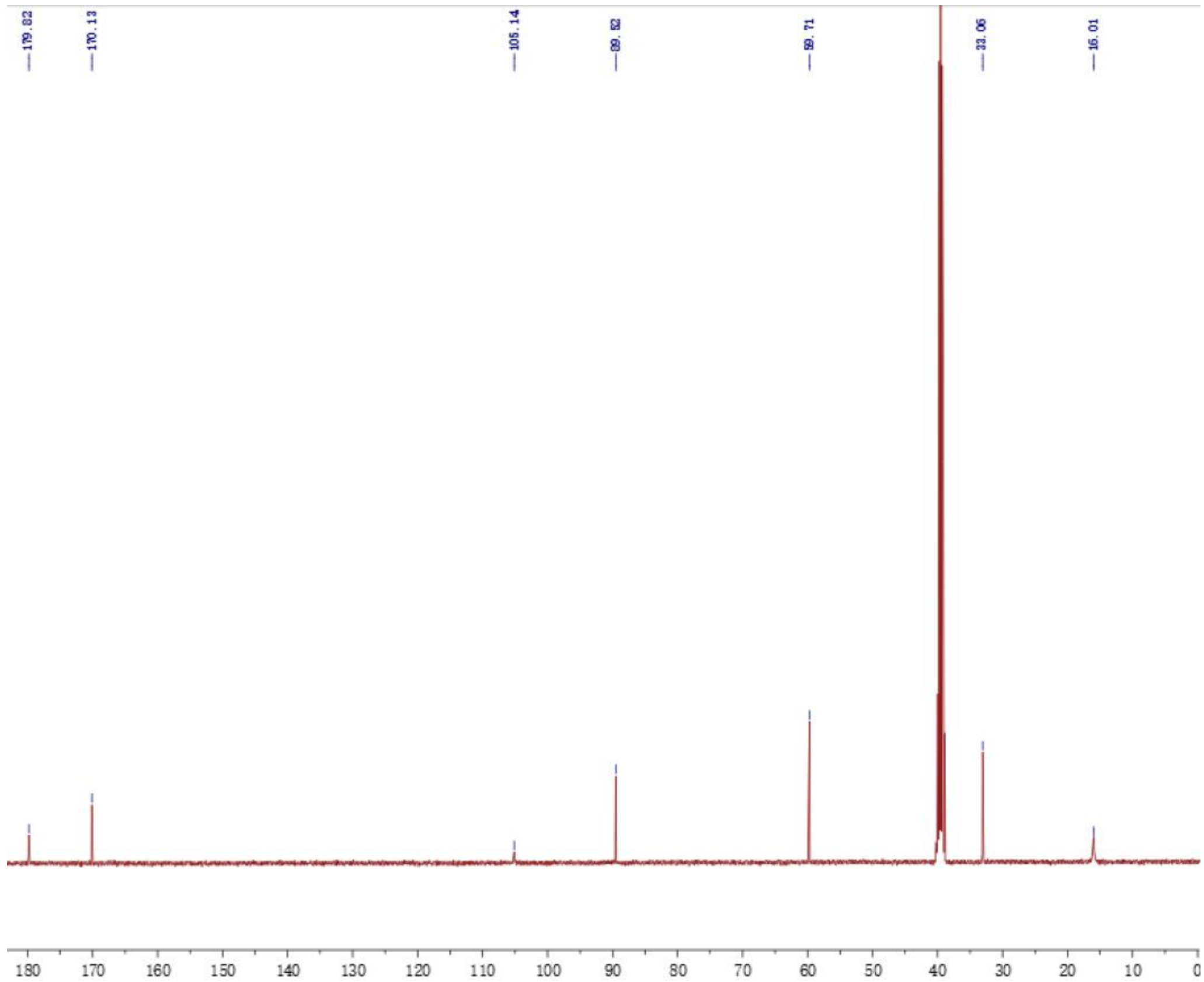

Figure S18. ^{13}C NMR (100 MHz) spectrum of **3** in DMSO-d_6

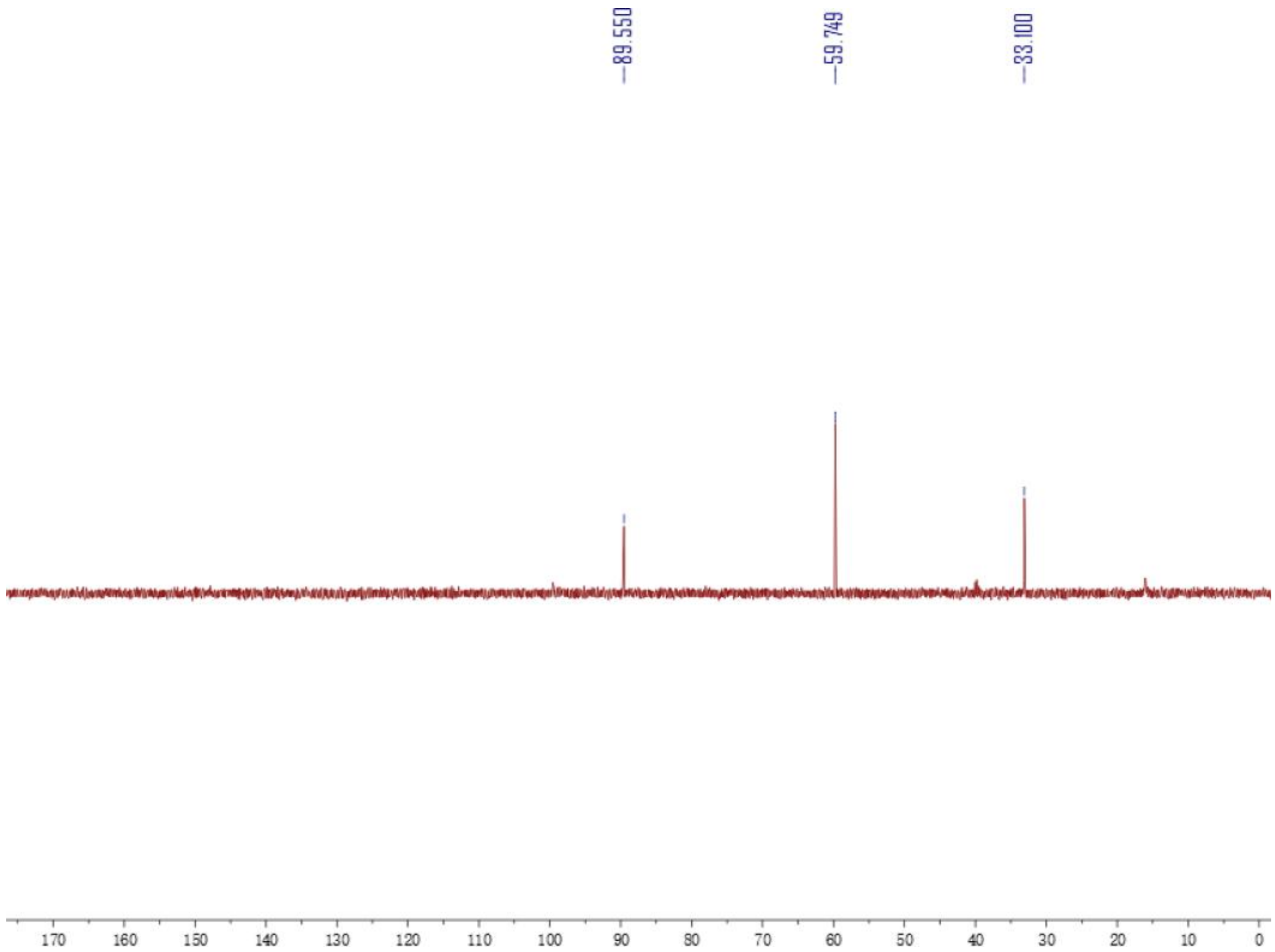

Figure S19. 135°-DEPT spectrum of **3** in DMSO-d_6

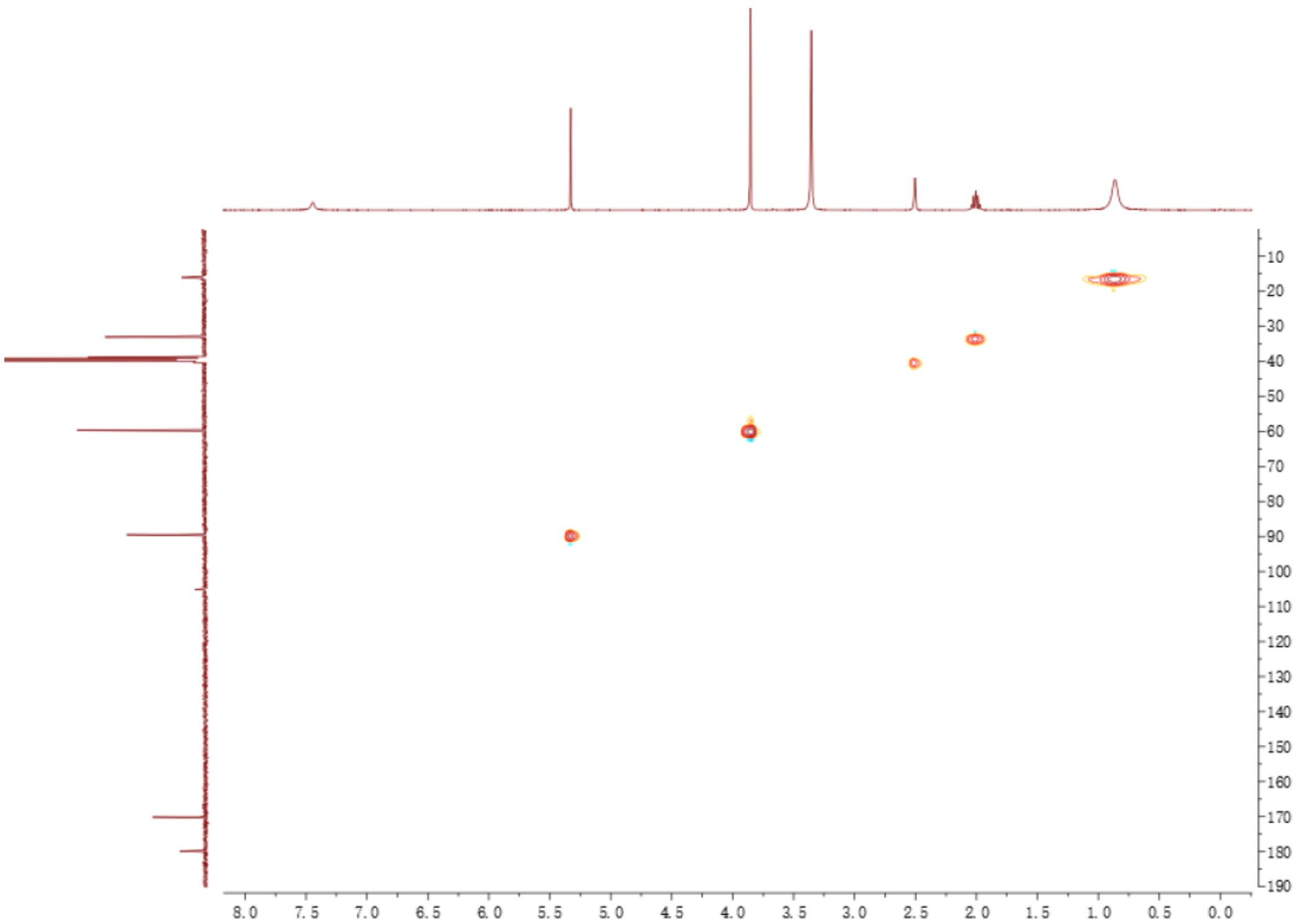

Figure S20. HSQC spectrum of **3** in DMSO-d_6

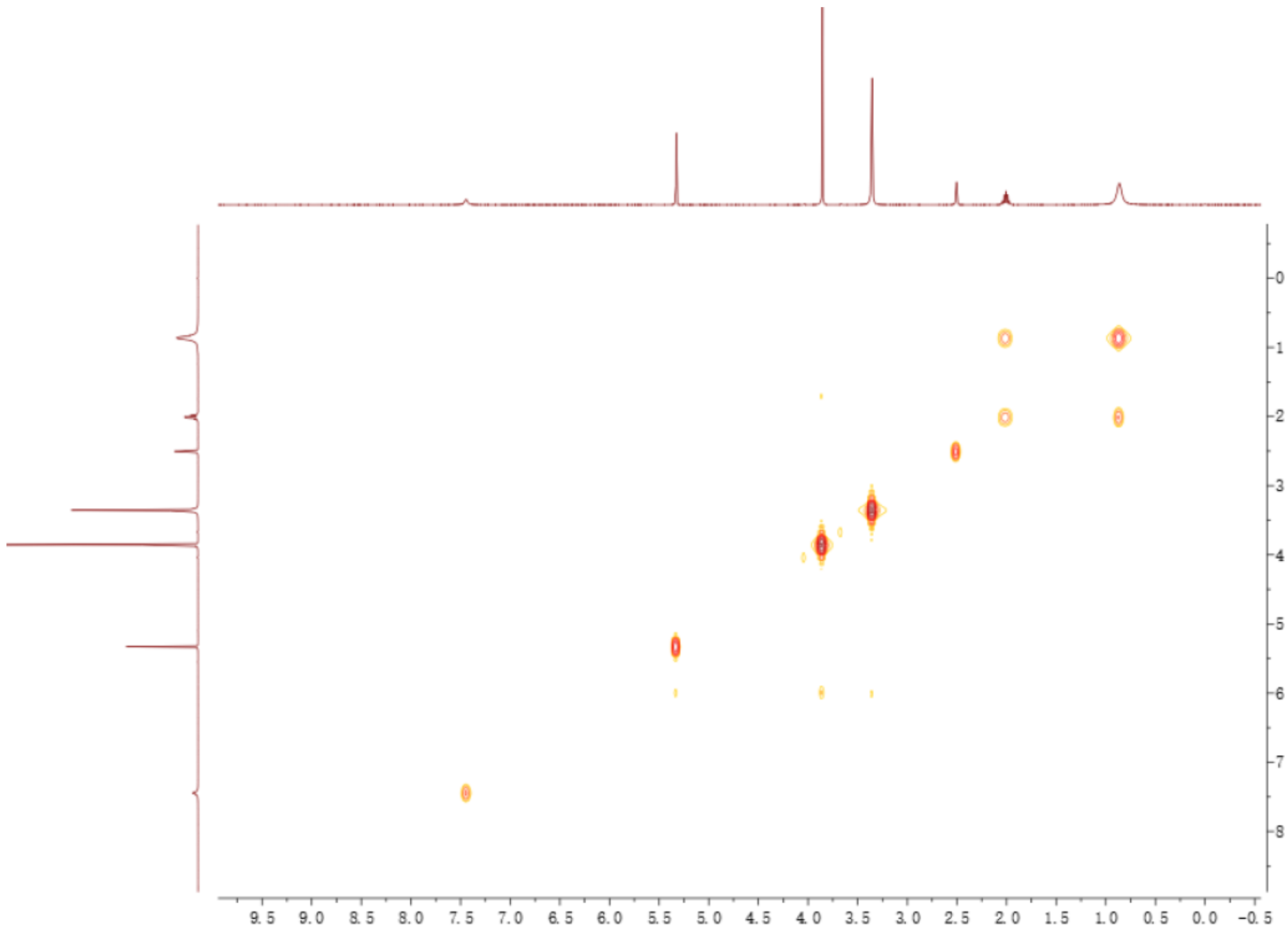

Figure S21. ^{1}H-^{1}H COSY spectrum of **3** in DMSO-d_6

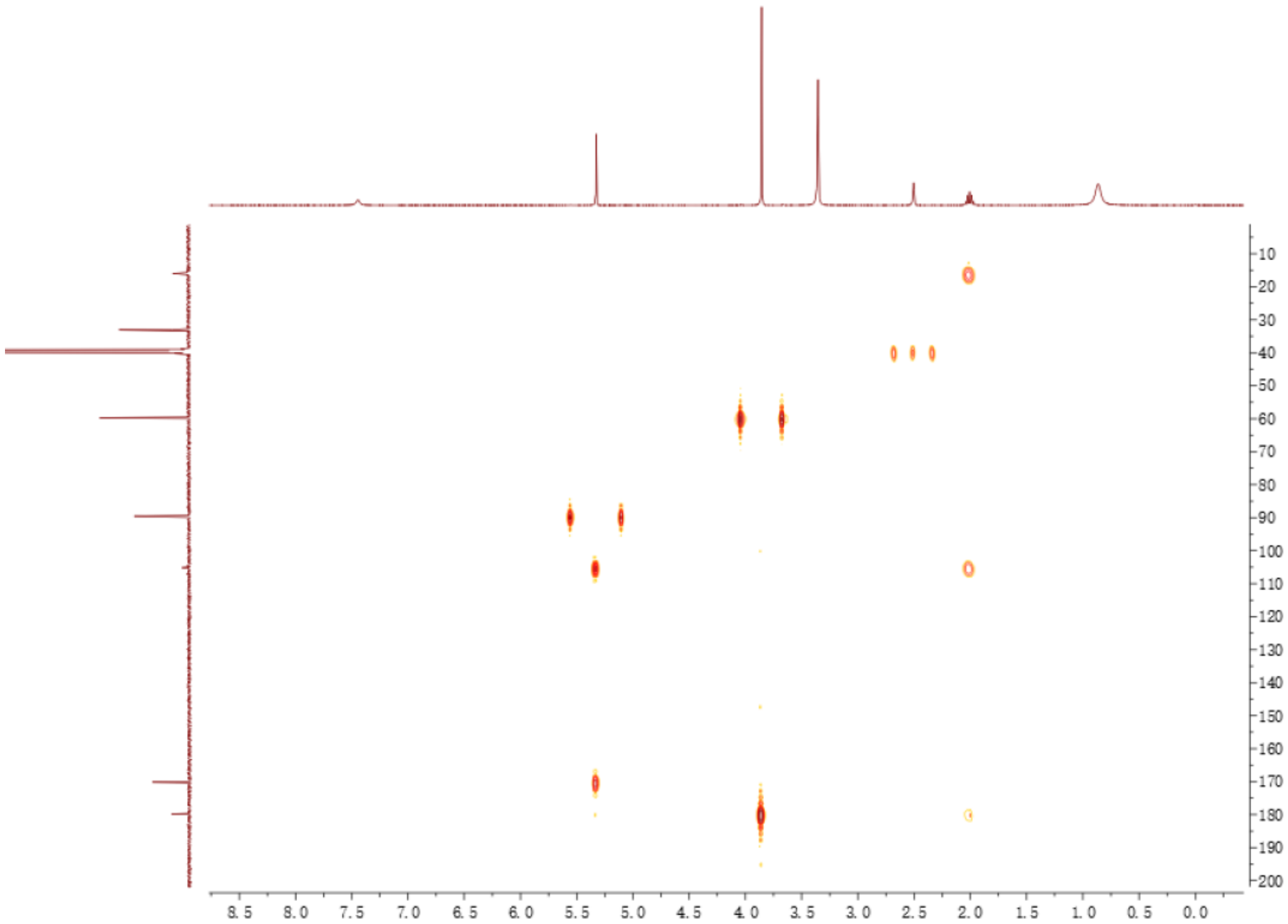

Figure S22. HMBC spectrum of **3** in DMSO-d_6

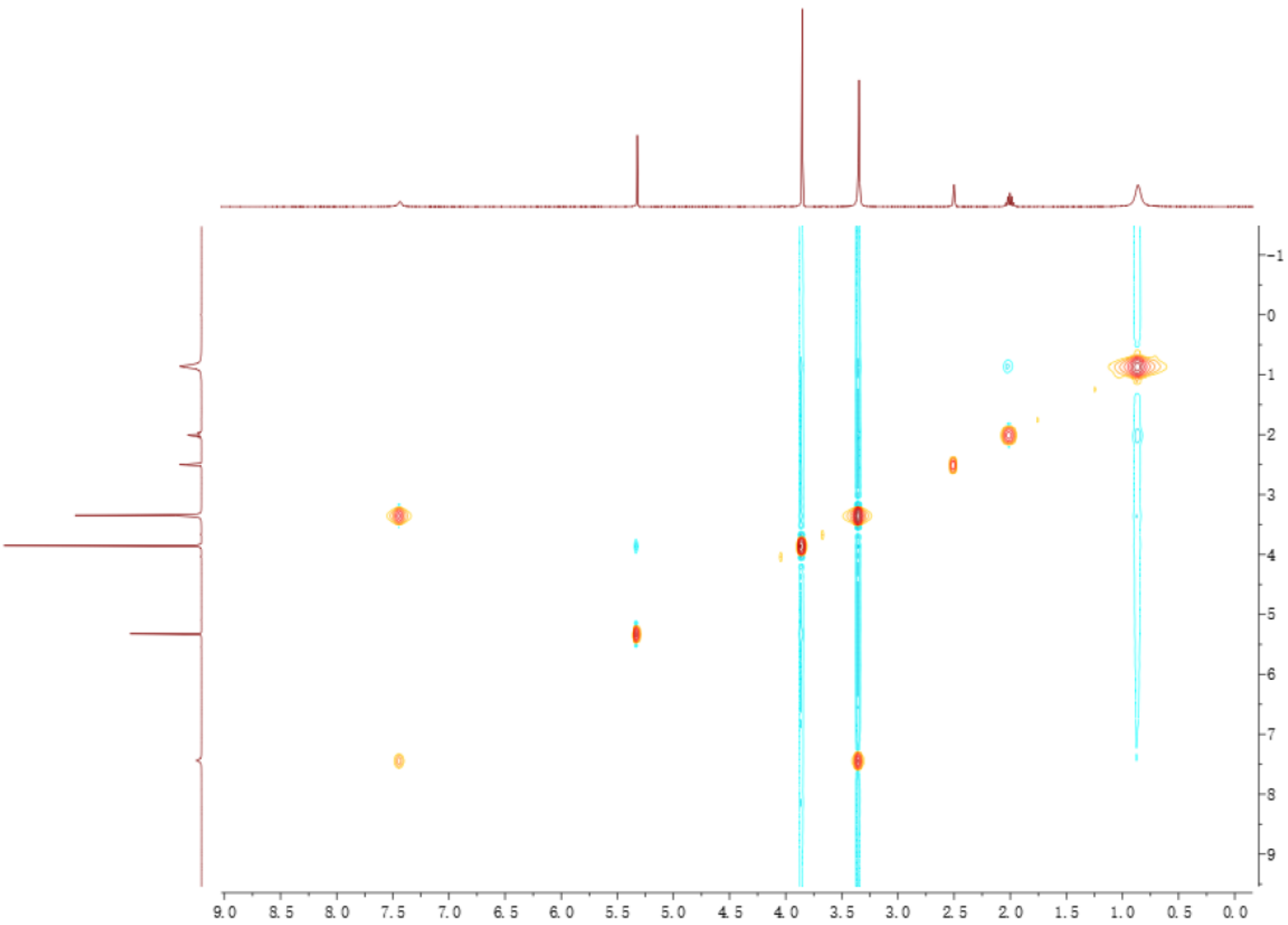

Figure S23. NOESY spectrum of **3** in DMSO-d_6

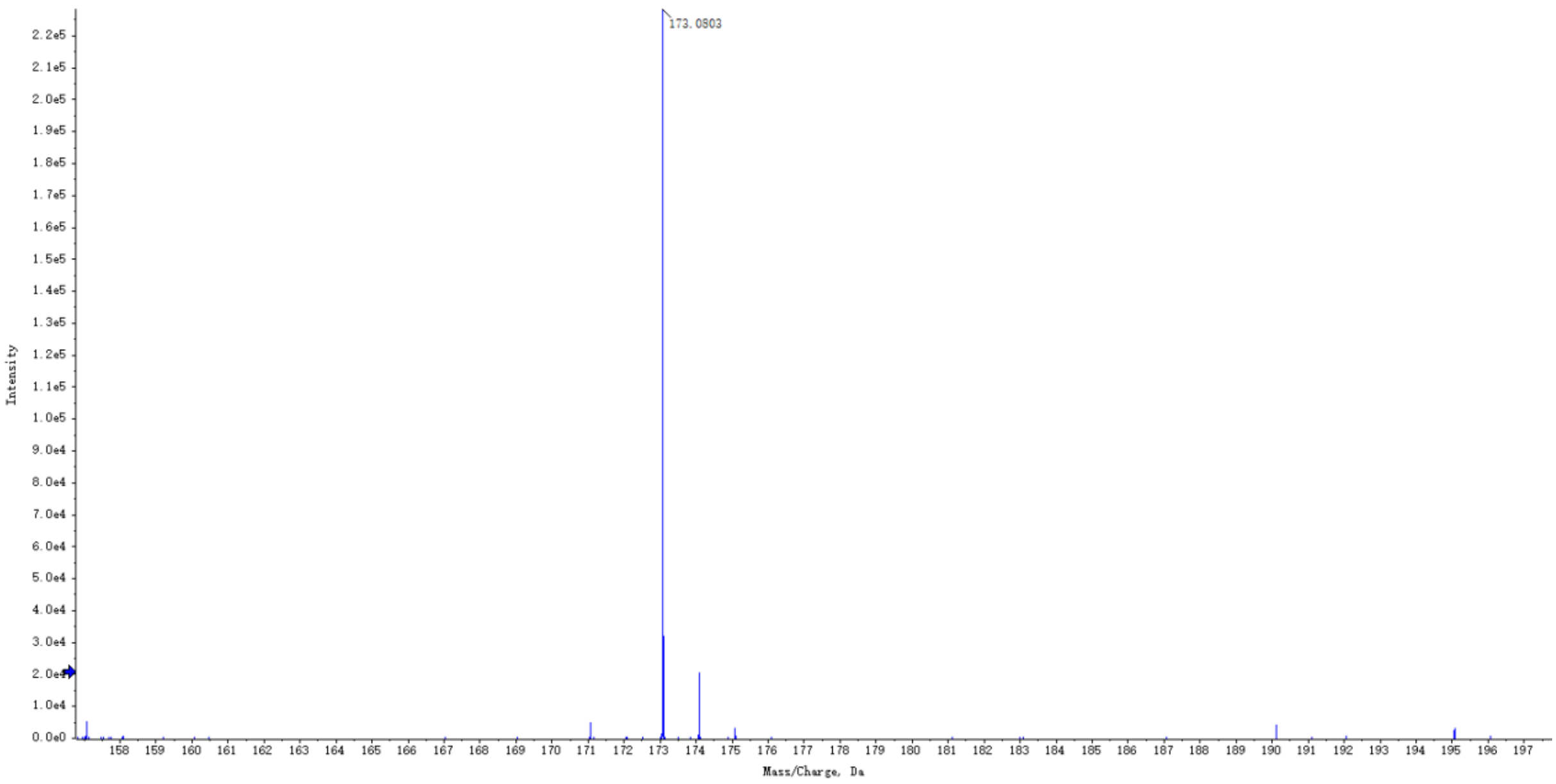

Figure S24. HR-ESI-MS spectrum of **3**

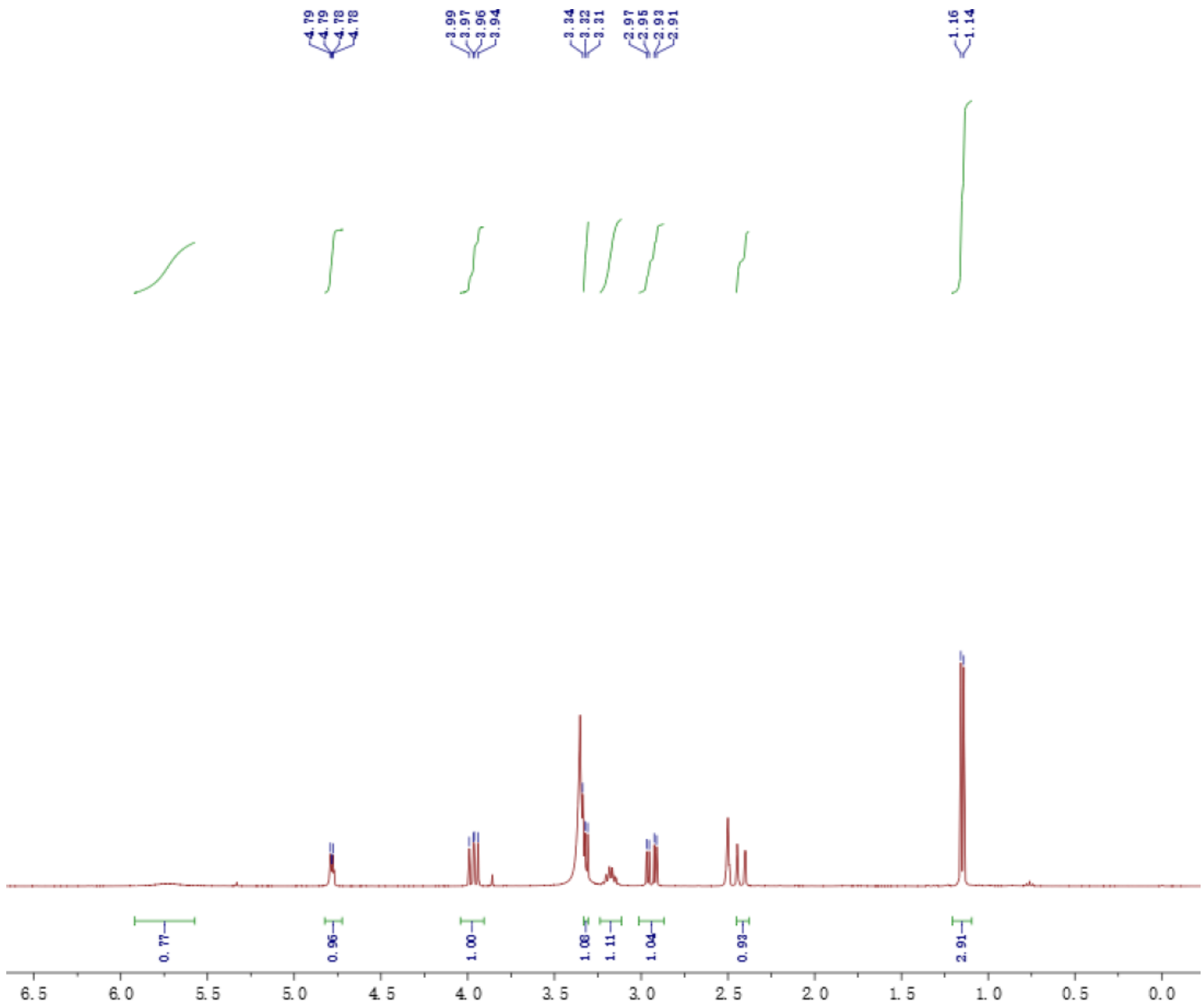

Figure S25. ^{1}H NMR (400 MHz) spectrum of **4** in DMSO-d_6

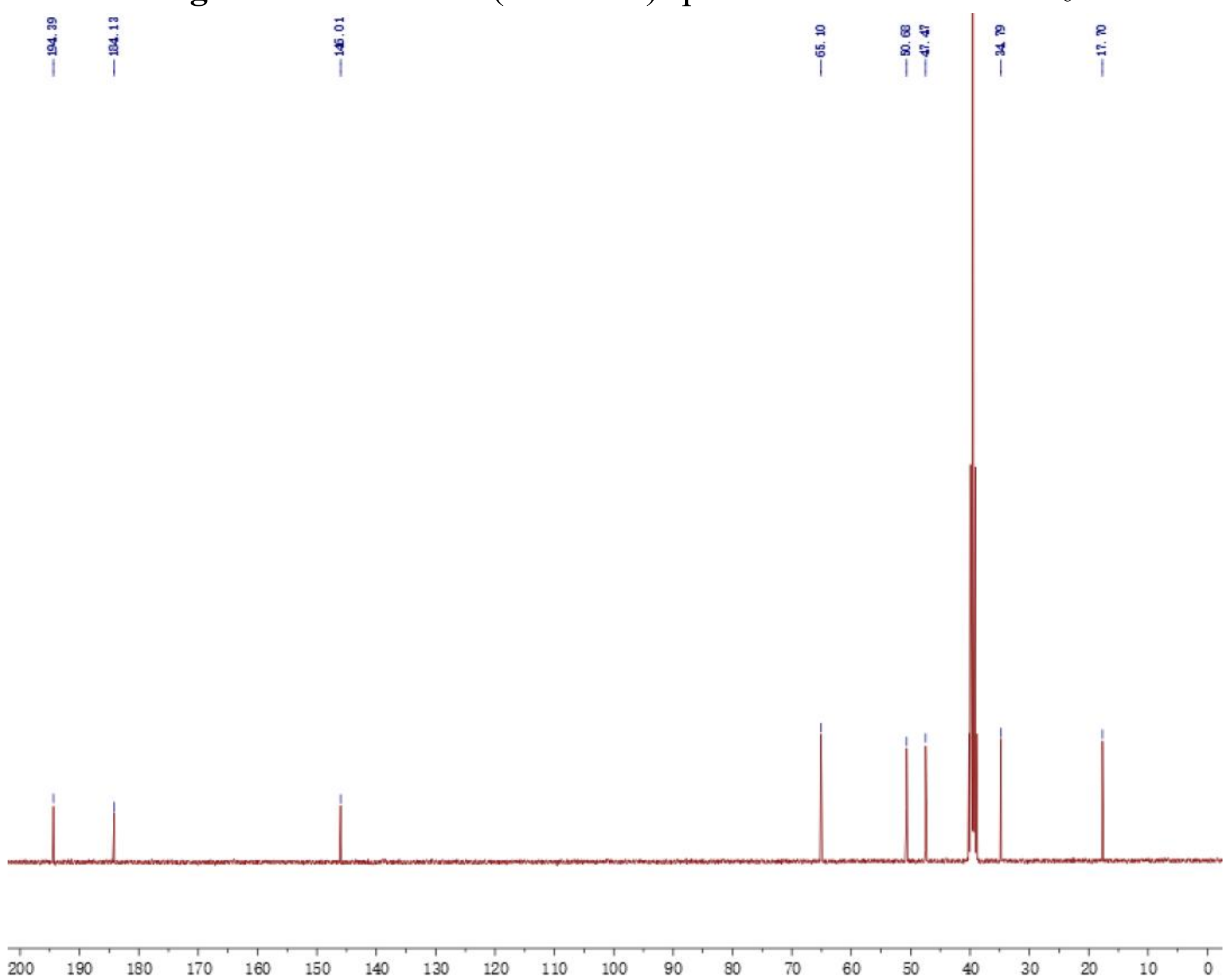

Figure S26. ^{13}C NMR (100 MHz) spectrum of **4** in DMSO-d_6

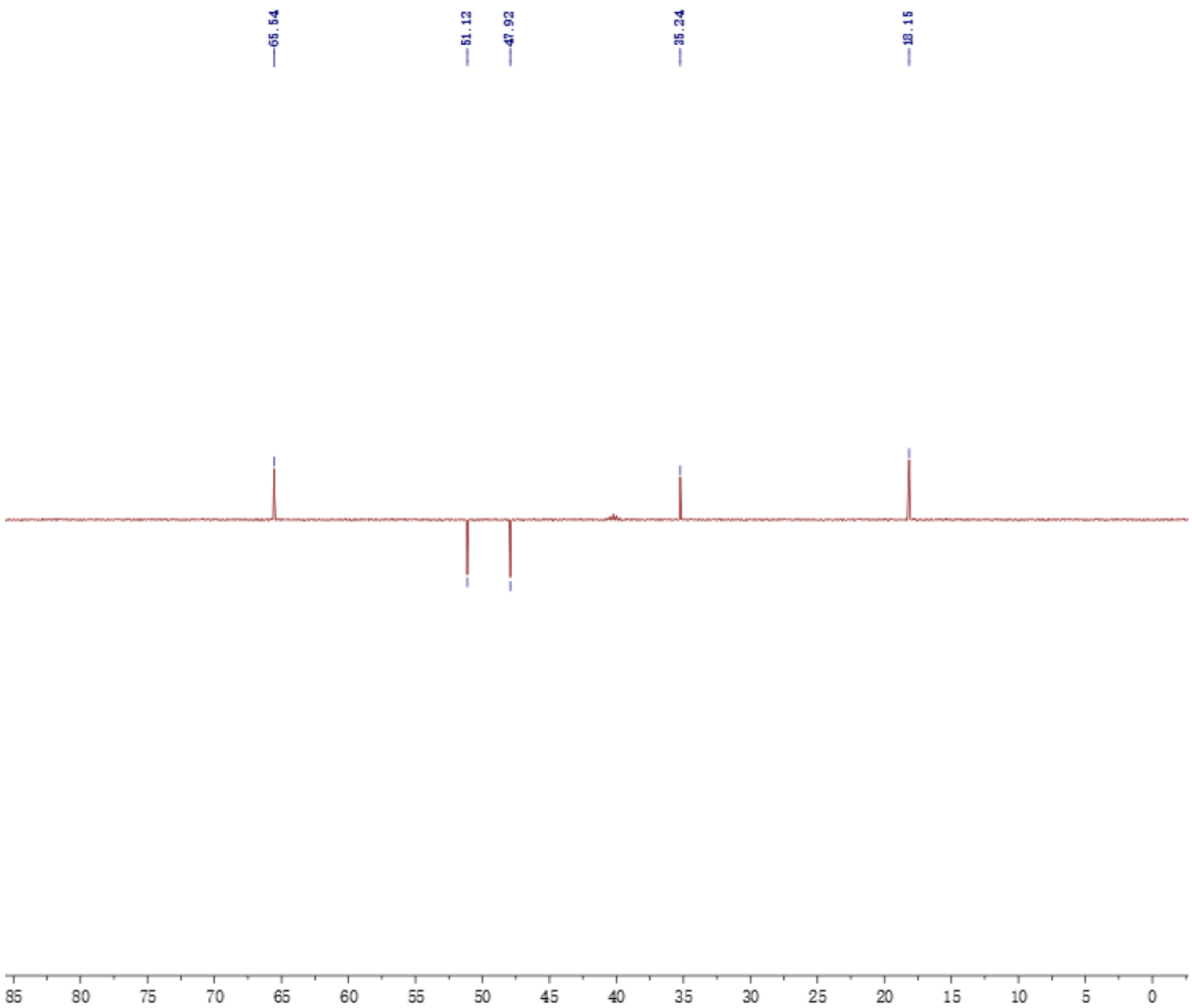

Figure S27. 135°-DEPT spectrum of **4** in DMSO-d_6

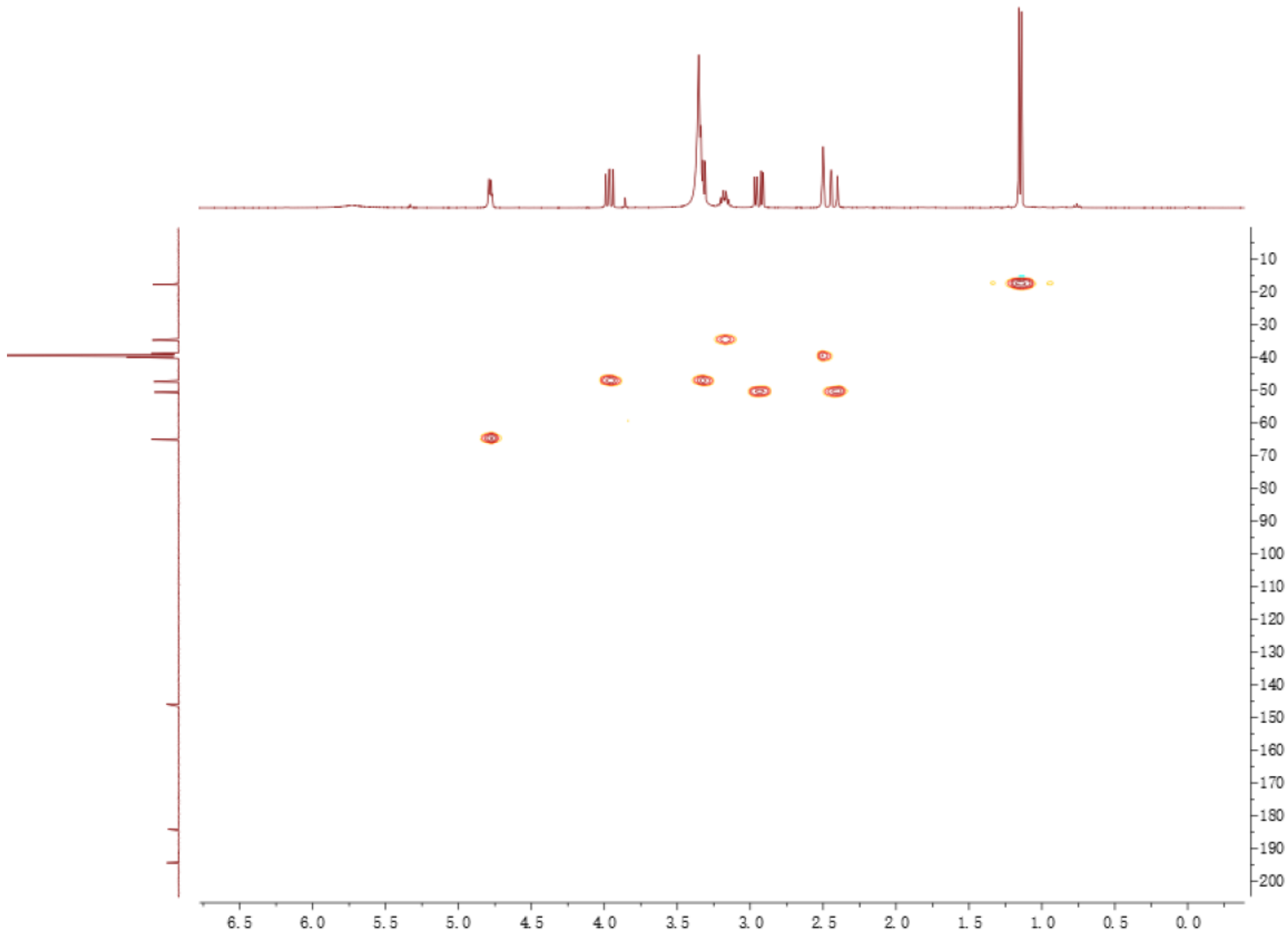

Figure S28. HSQC spectrum of **4** in DMSO-d_6

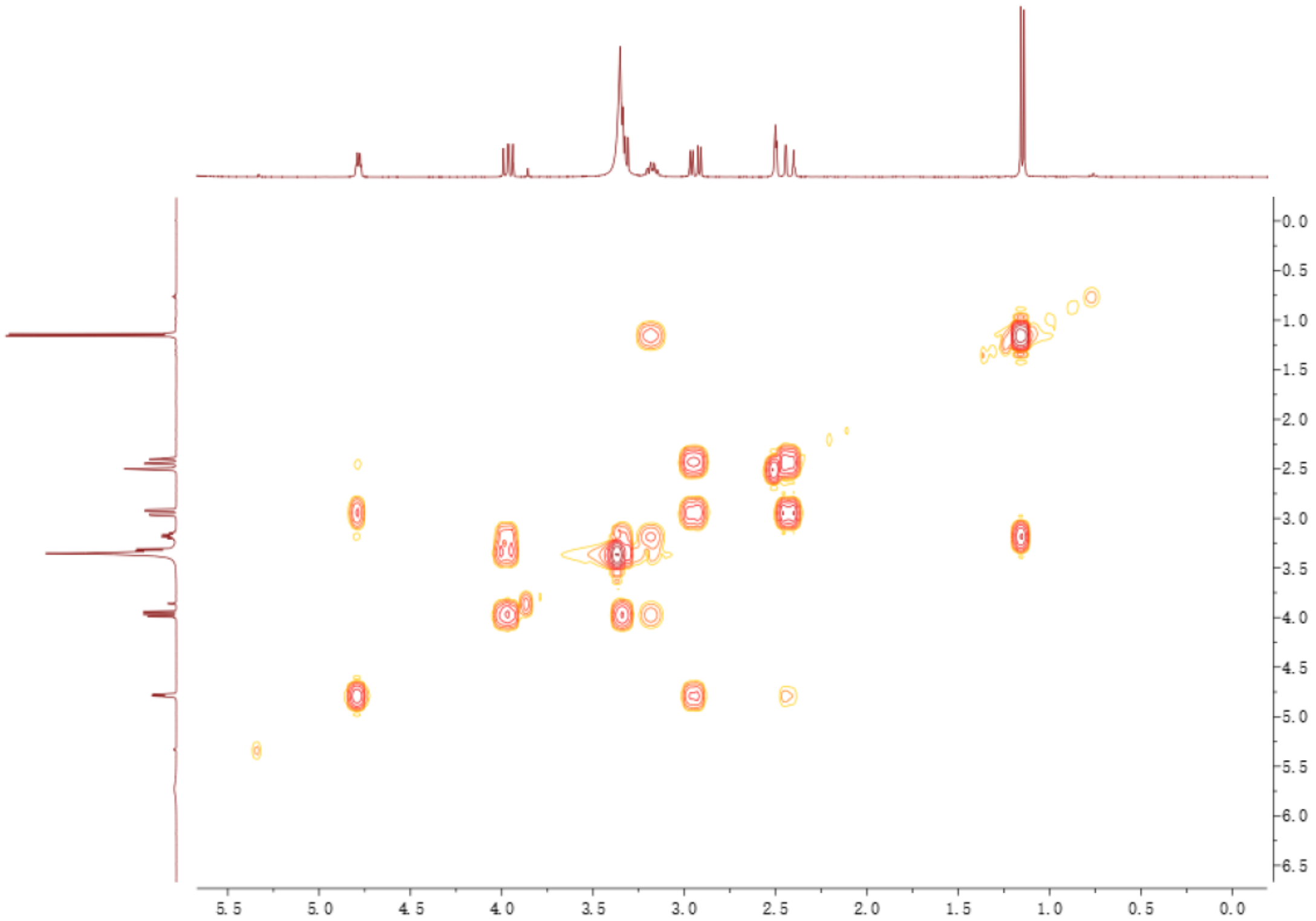

Figure S29. ^{1}H-^{1}H COSY spectrum of **4** in DMSO-d_6

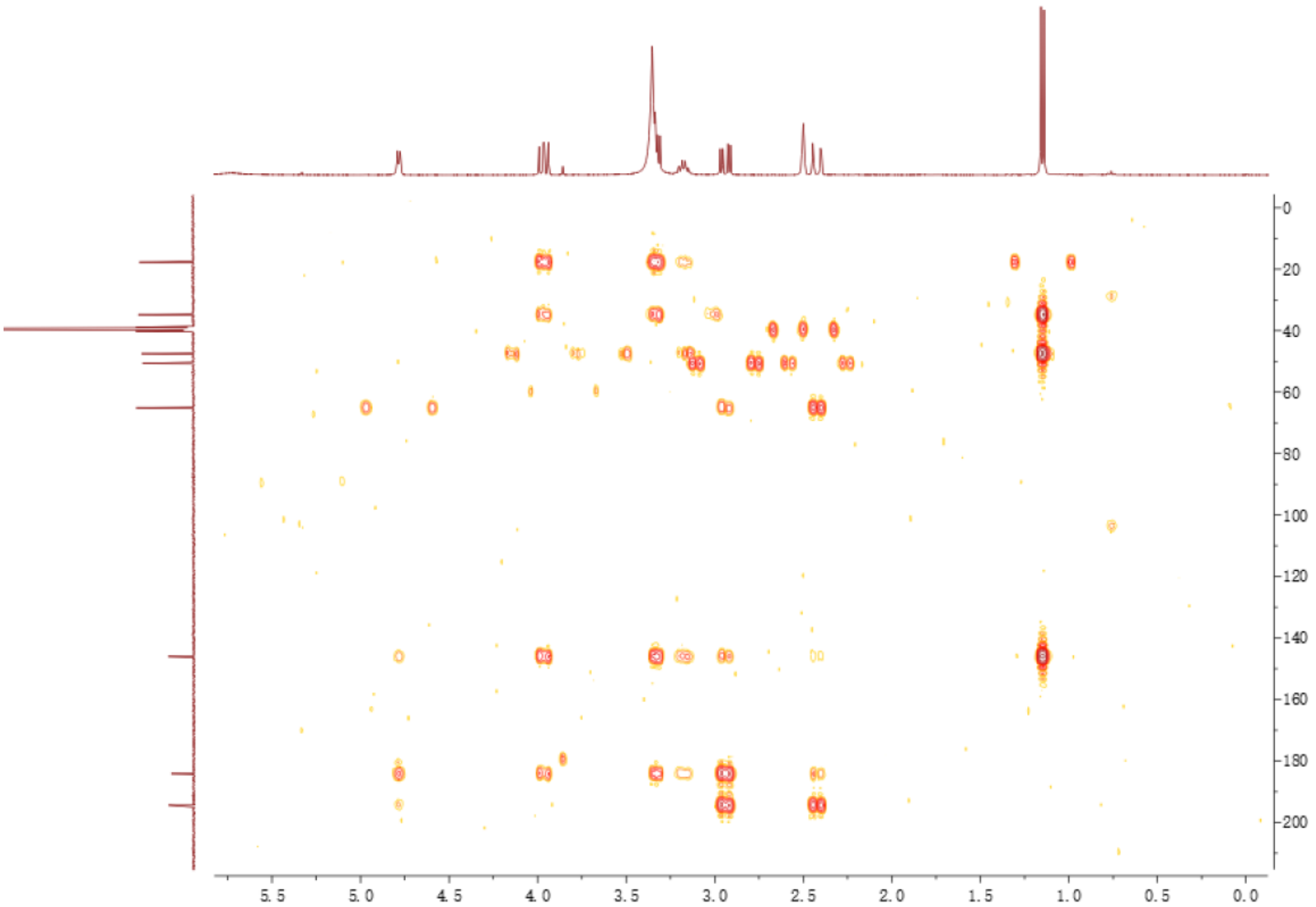

Figure S30. HMBC spectrum of **4** in DMSO-d_6

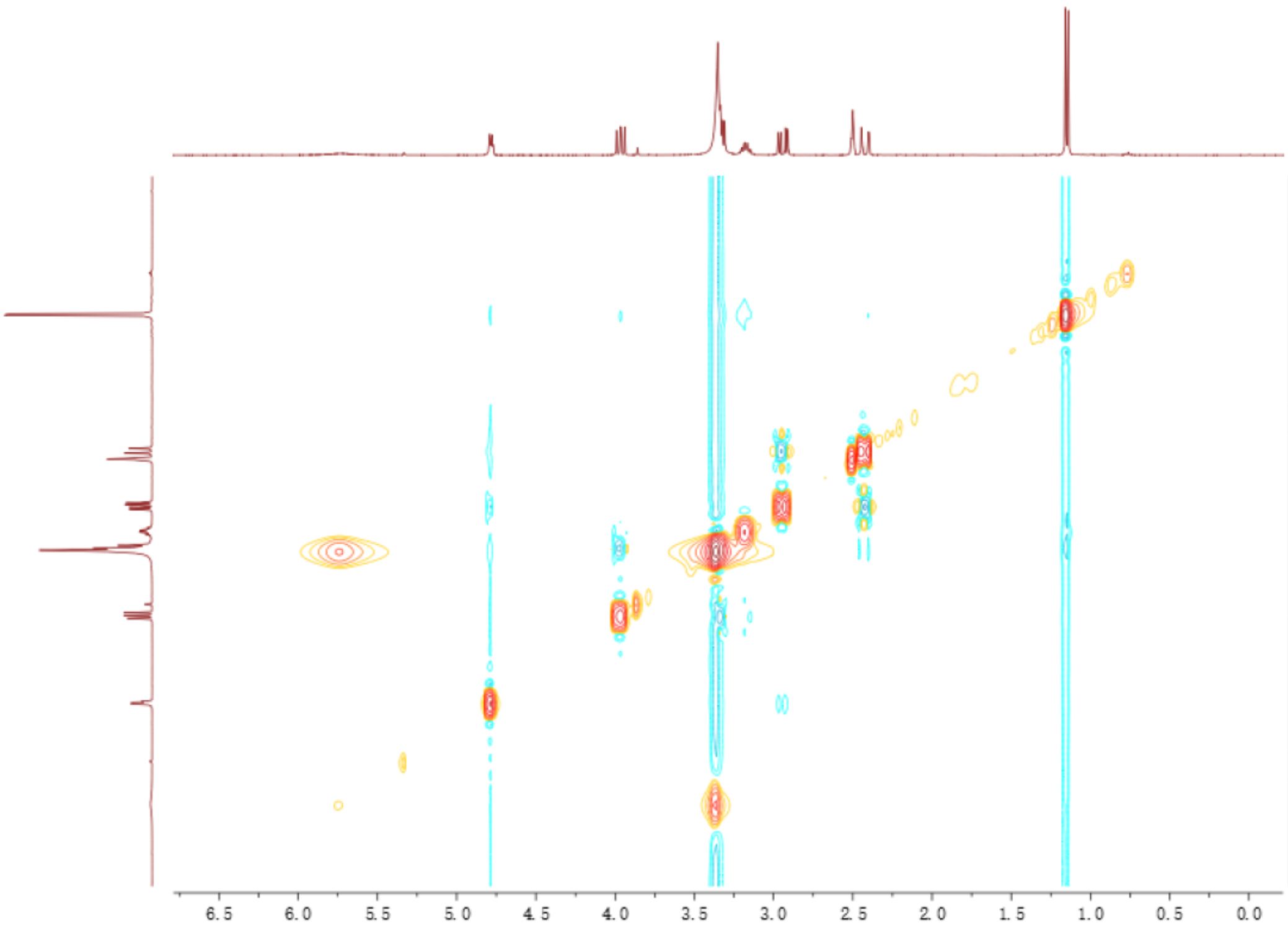

Figure S31. NOESY spectrum of **4** in DMSO-d_6

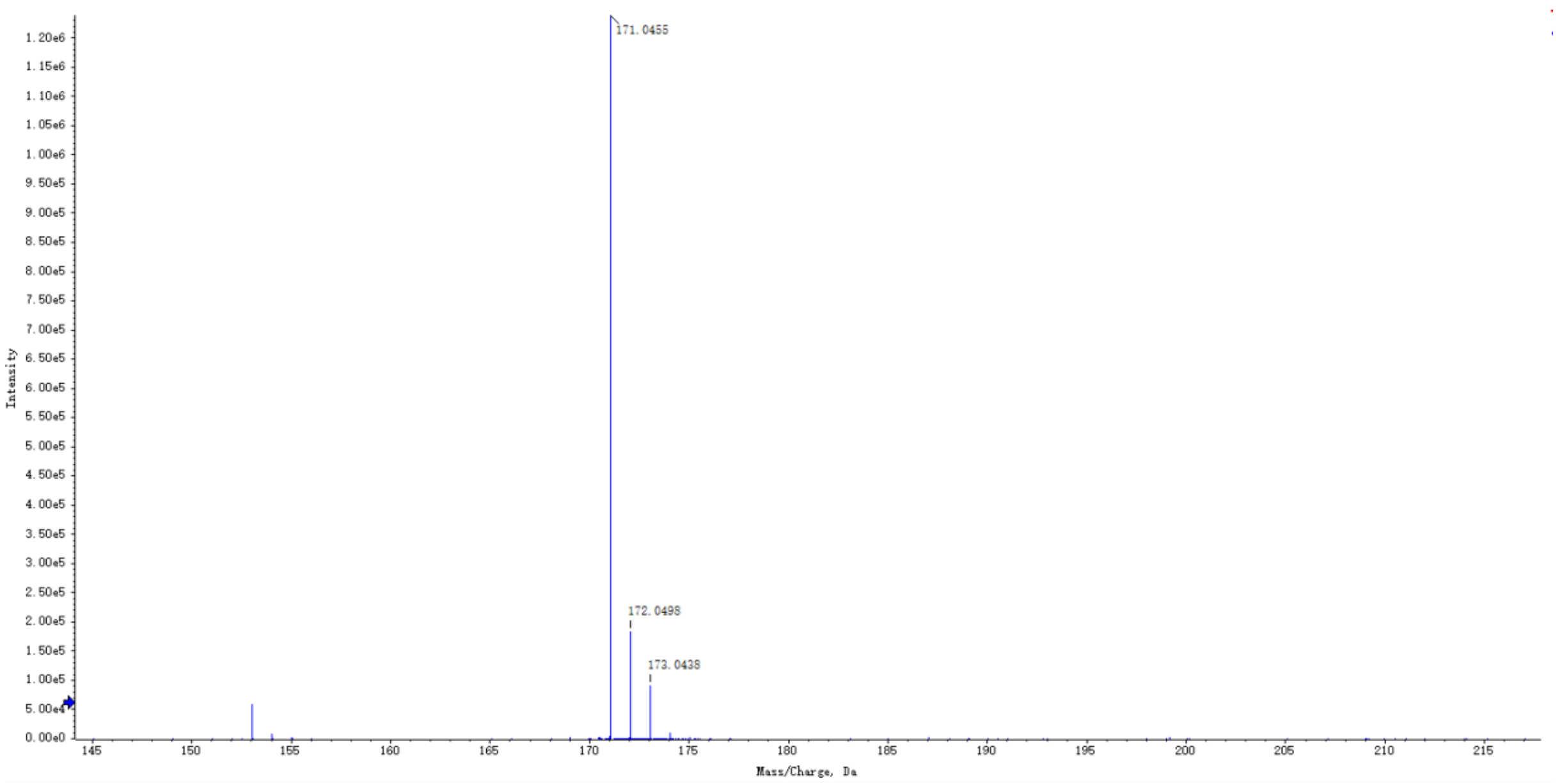

Figure S32. HR-ESI-MS spectrum of **4**

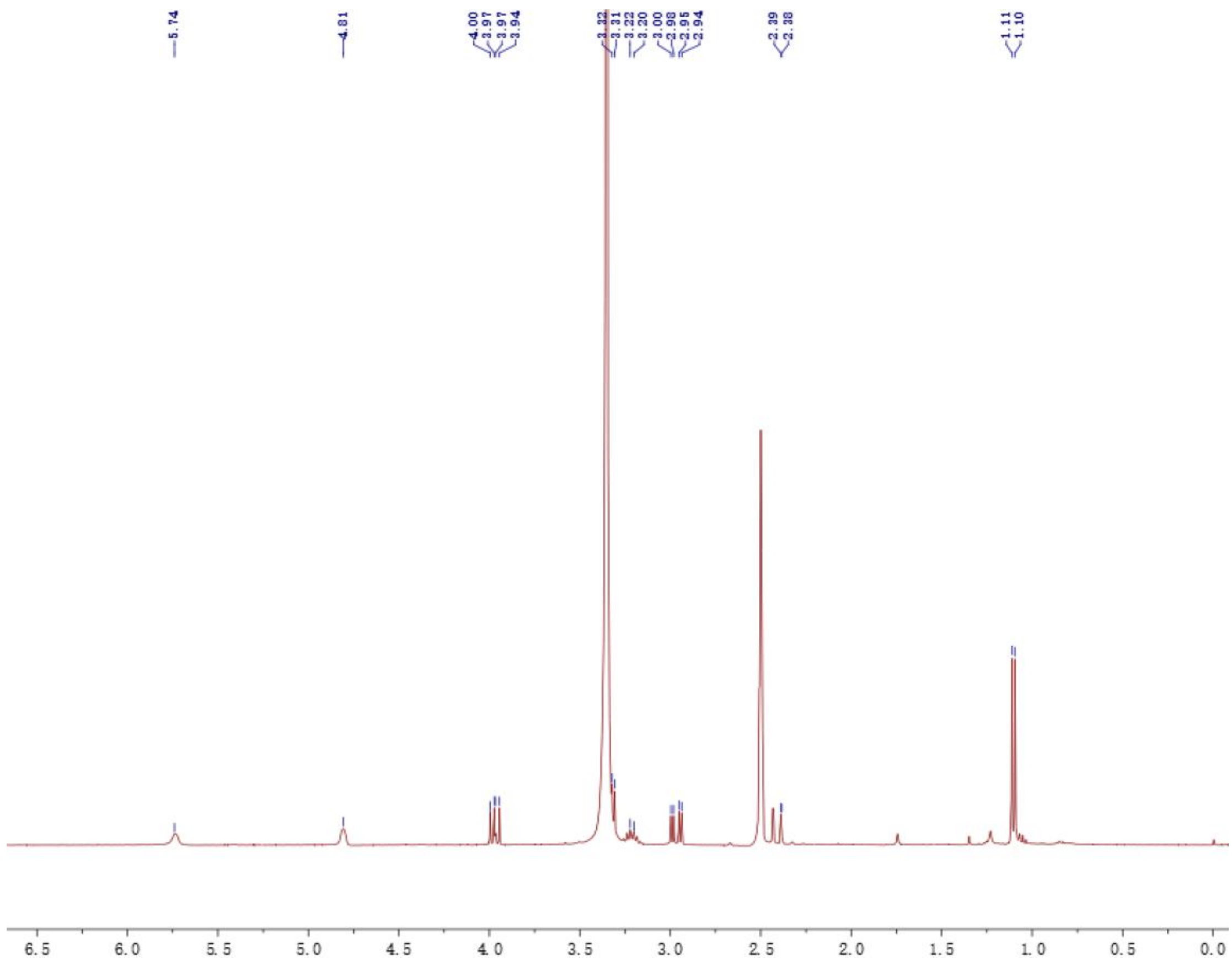

Figure S33. ^{1}H NMR (400 MHz) spectrum of **5** in DMSO-d_6

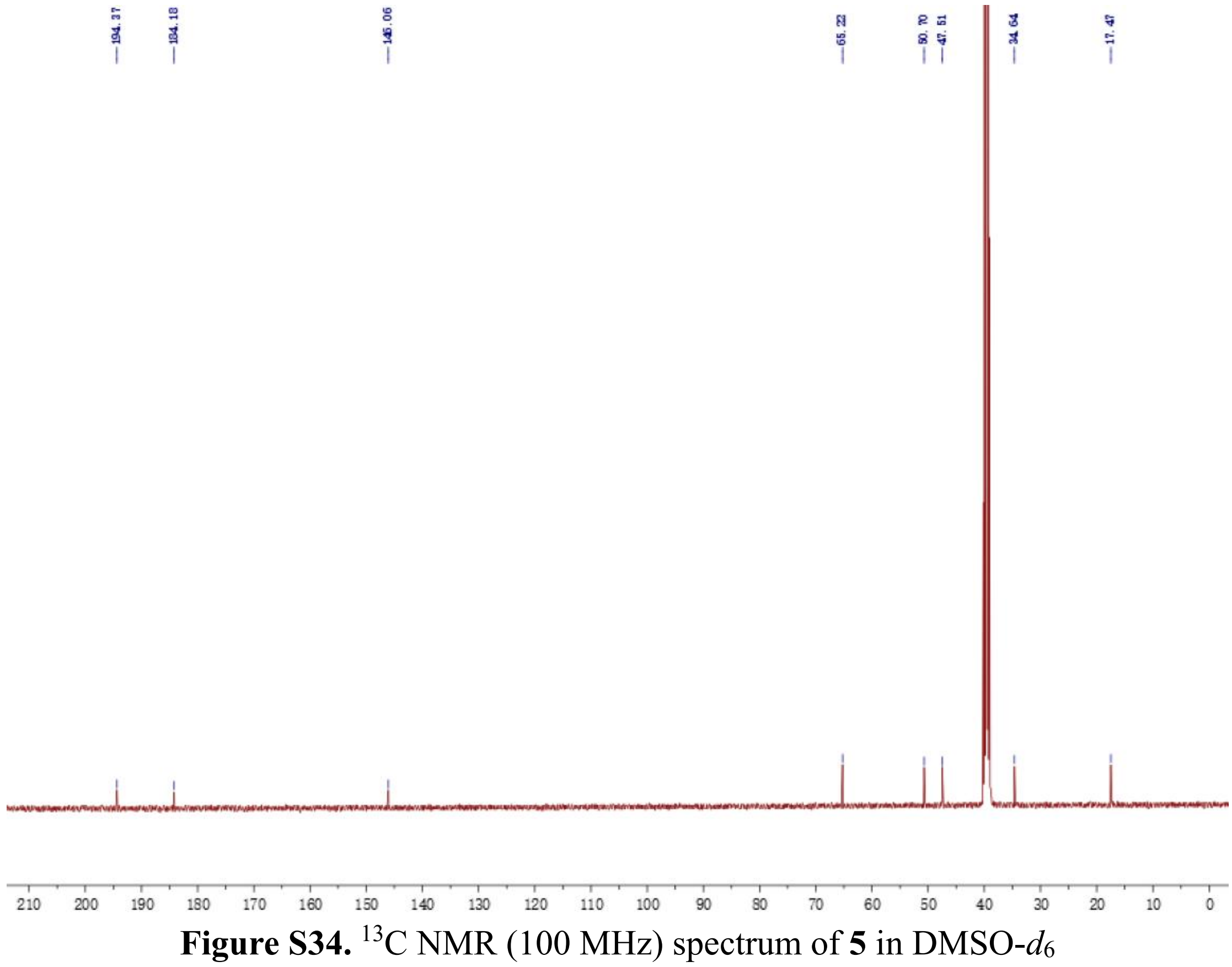

Figure S34. ^{13}C NMR (100 MHz) spectrum of **5** in DMSO-d_6

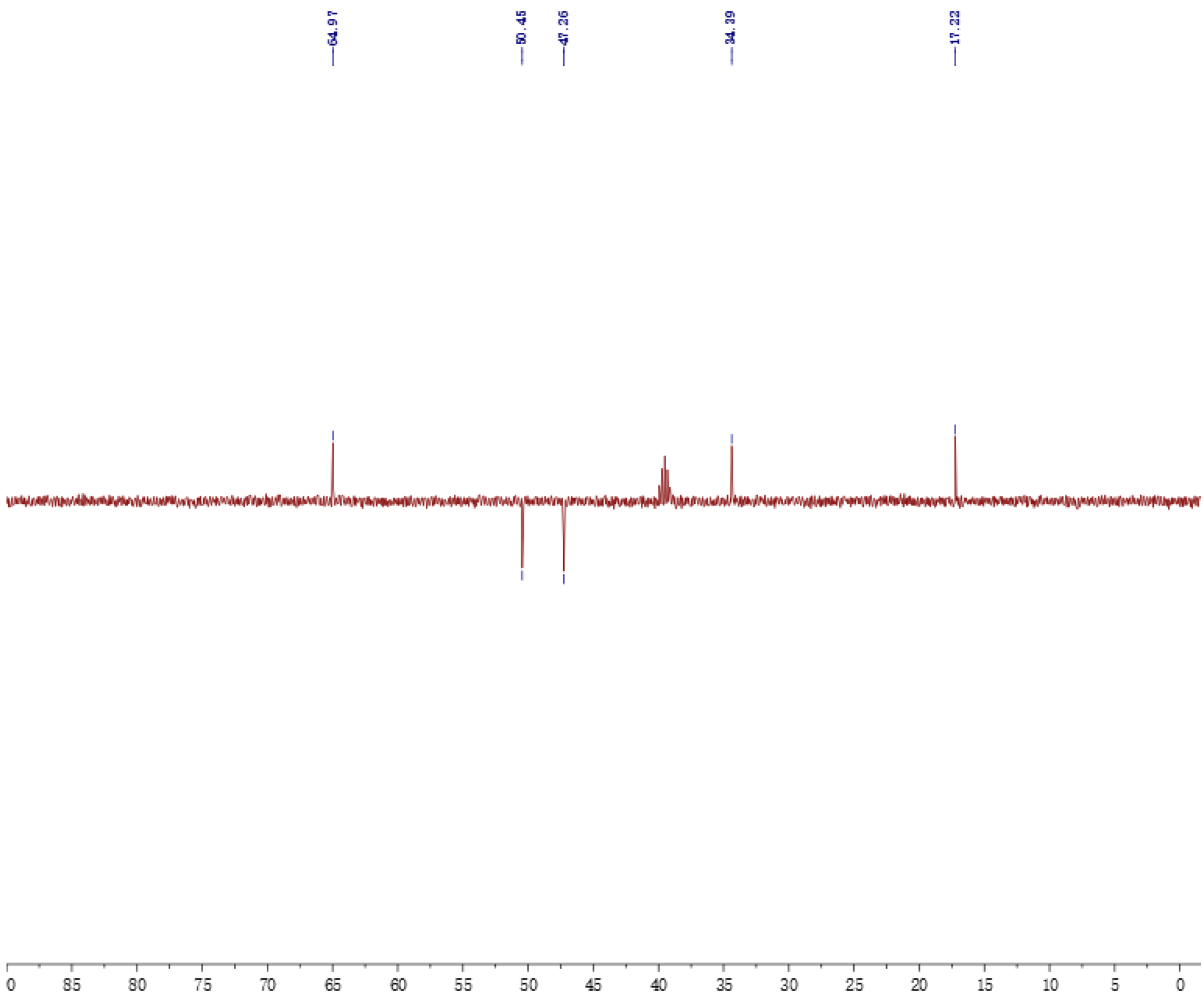

Figure S35. 135°-DEPT spectrum of **5** in DMSO-d_6

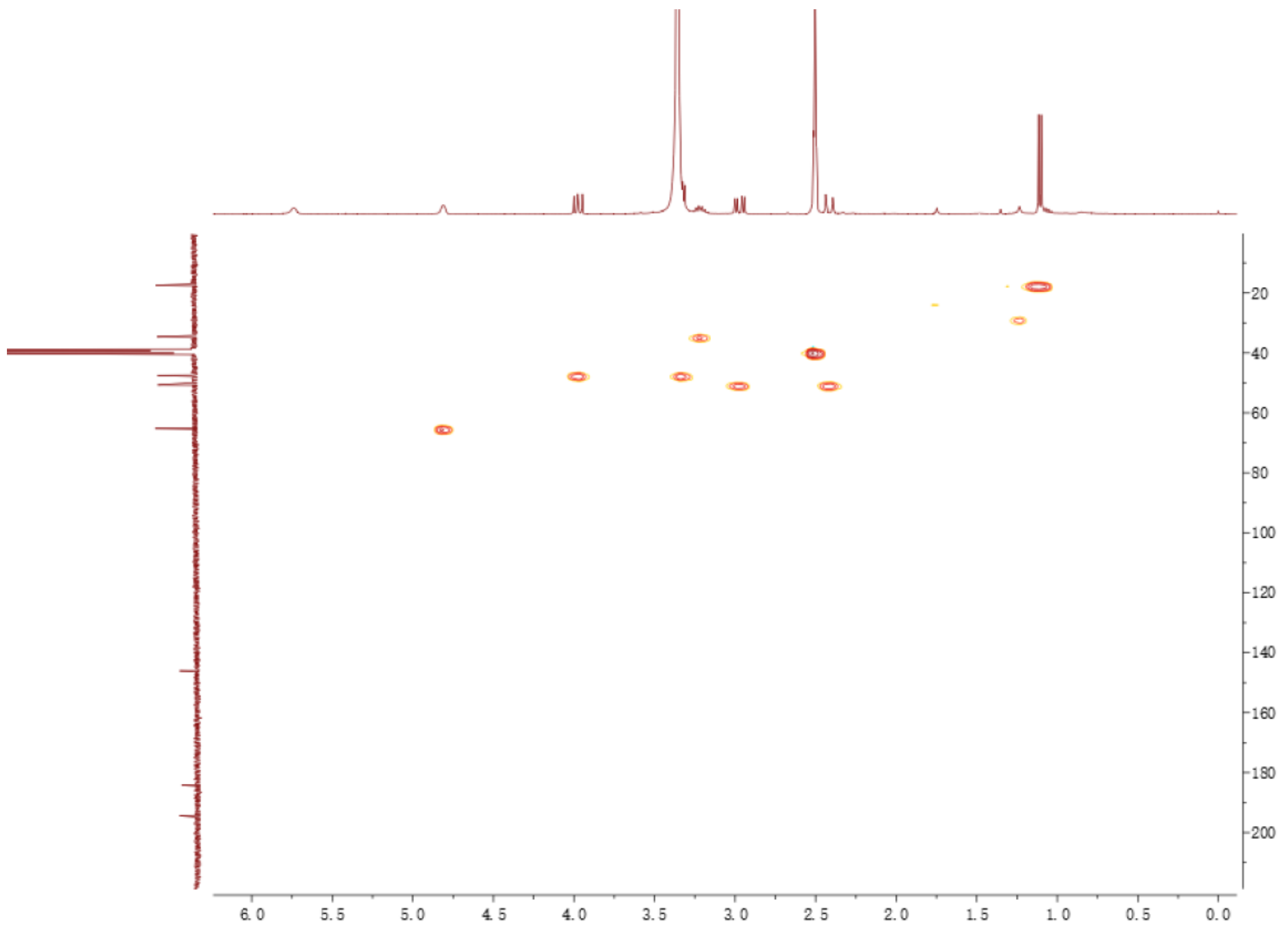

Figure S36. HSQC spectrum of **5** in DMSO-d_6

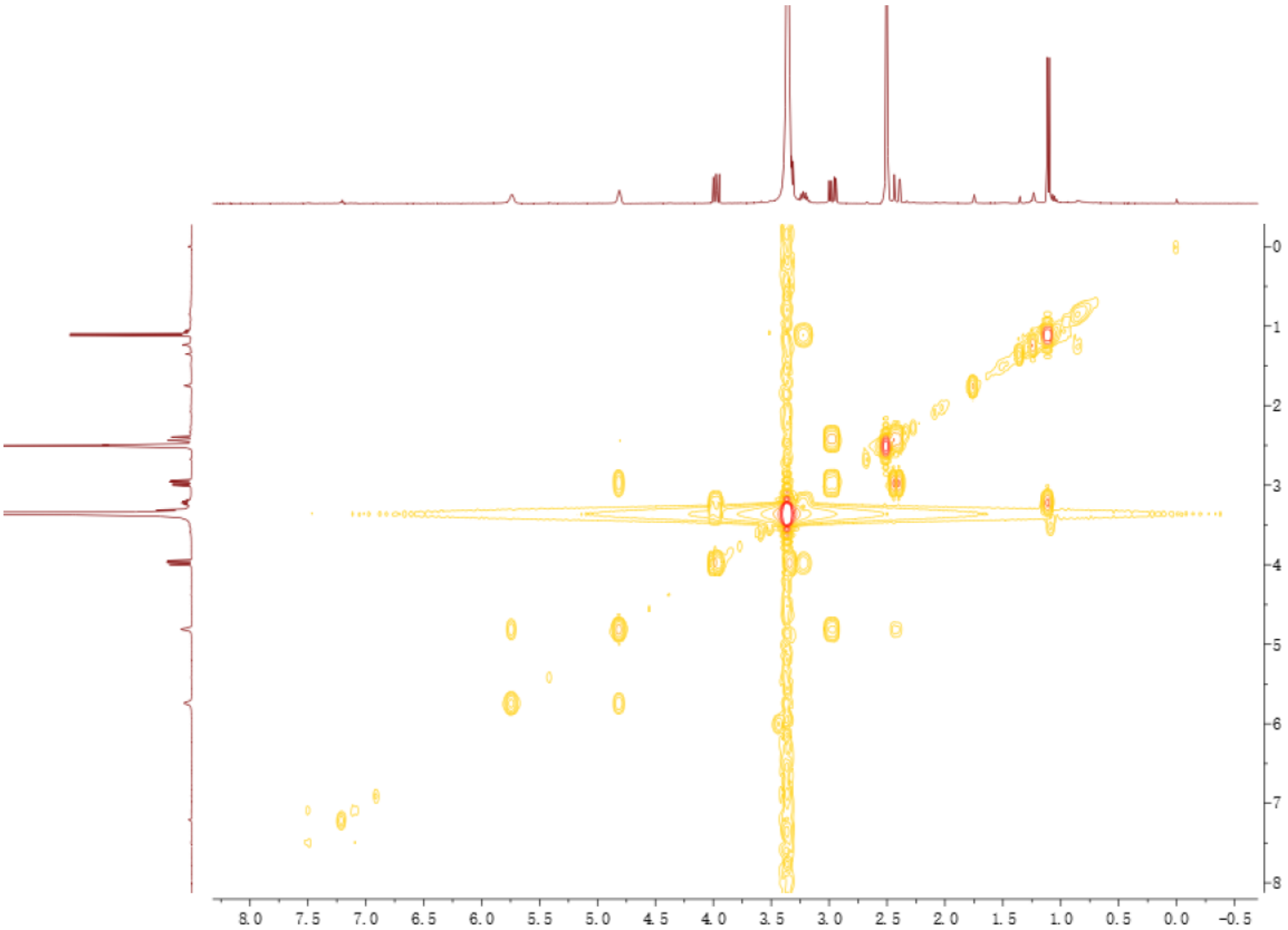

Figure S37. ^{1}H-^{1}H COSY spectrum of **5** in DMSO-d_6

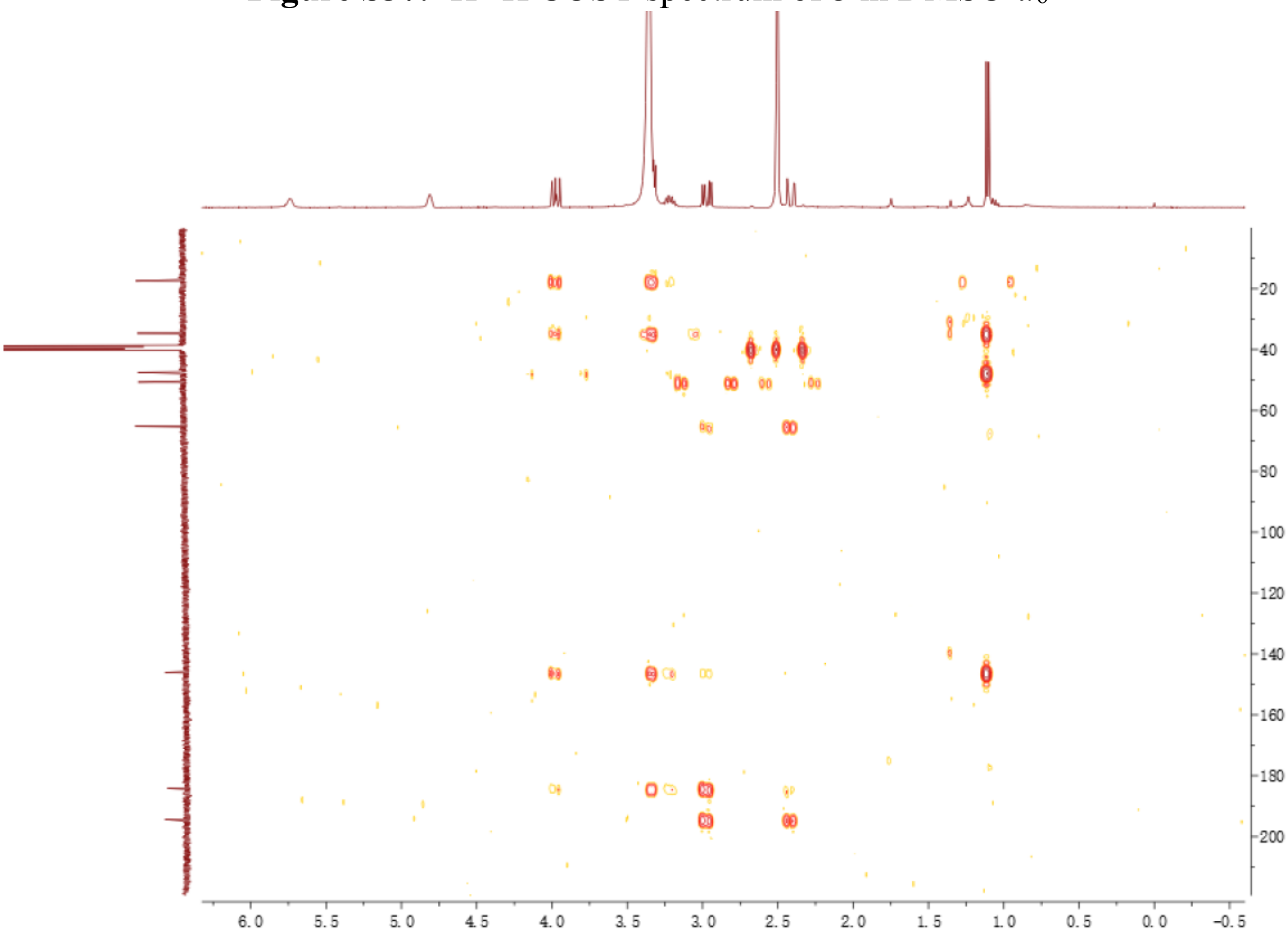

Figure S38. HMBC spectrum of **5** in DMSO-d_6

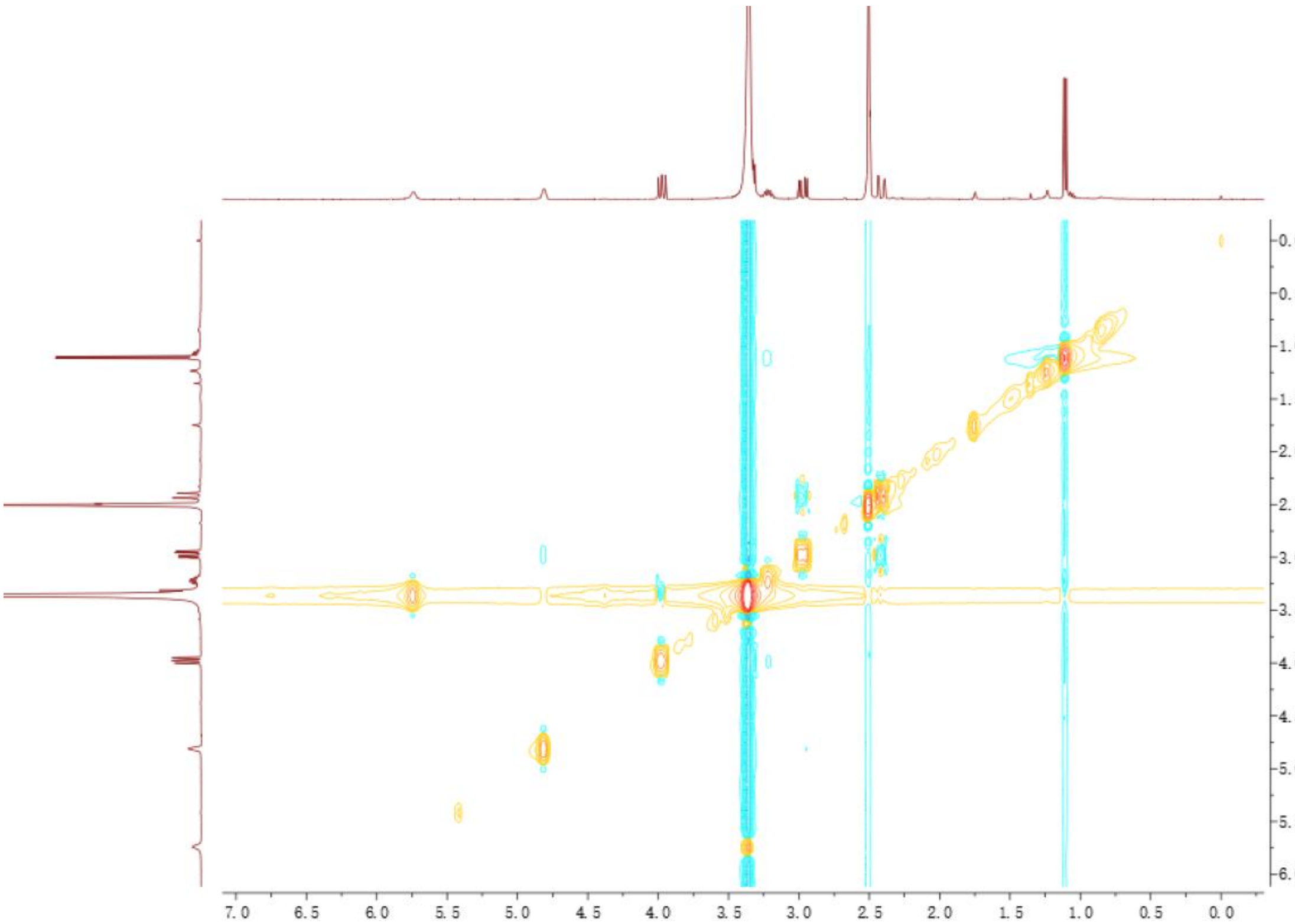

Figure S39. NOESY spectrum of **5** in DMSO-d_6

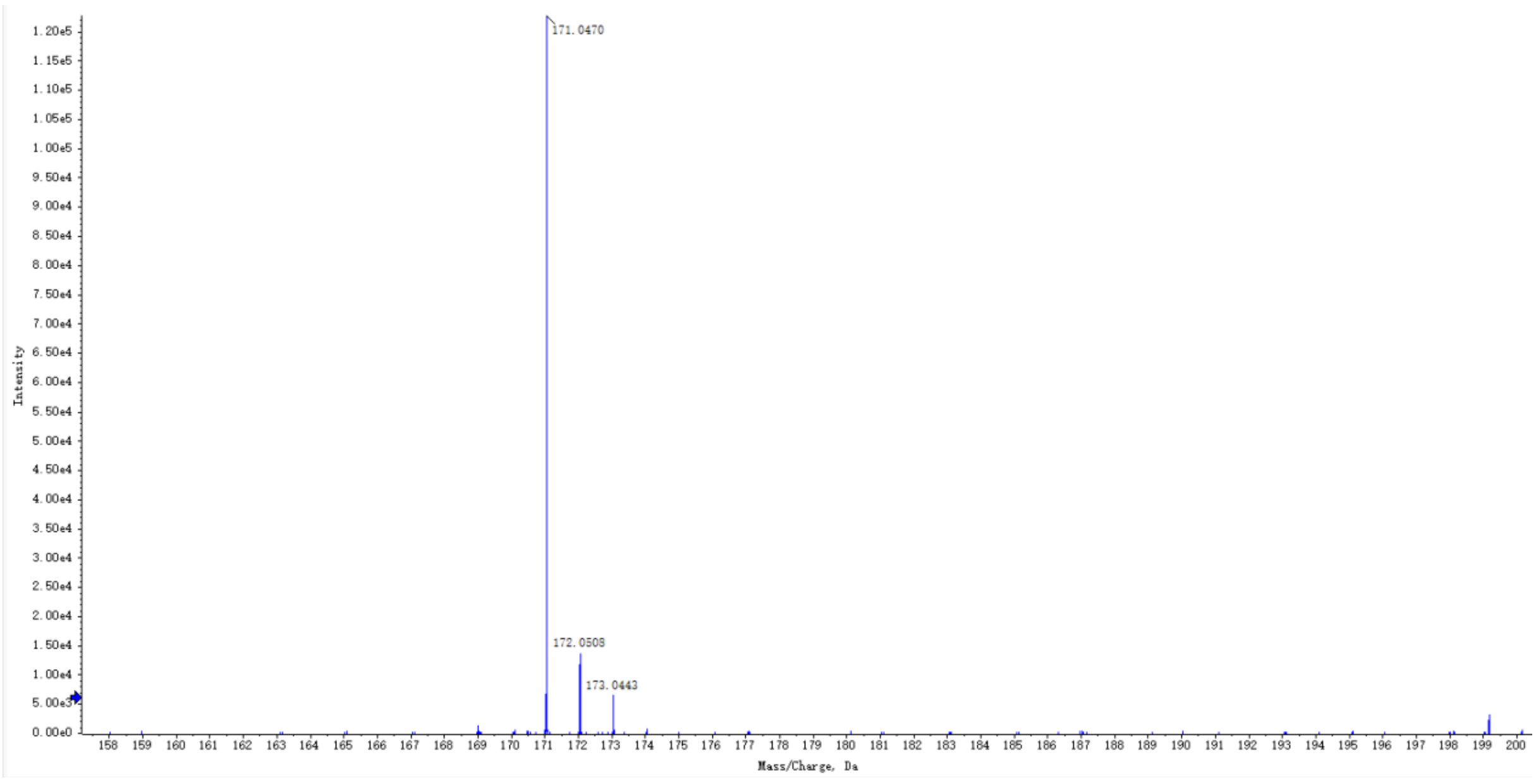

Figure S40. HR-ESI-MS spectrum of **5**

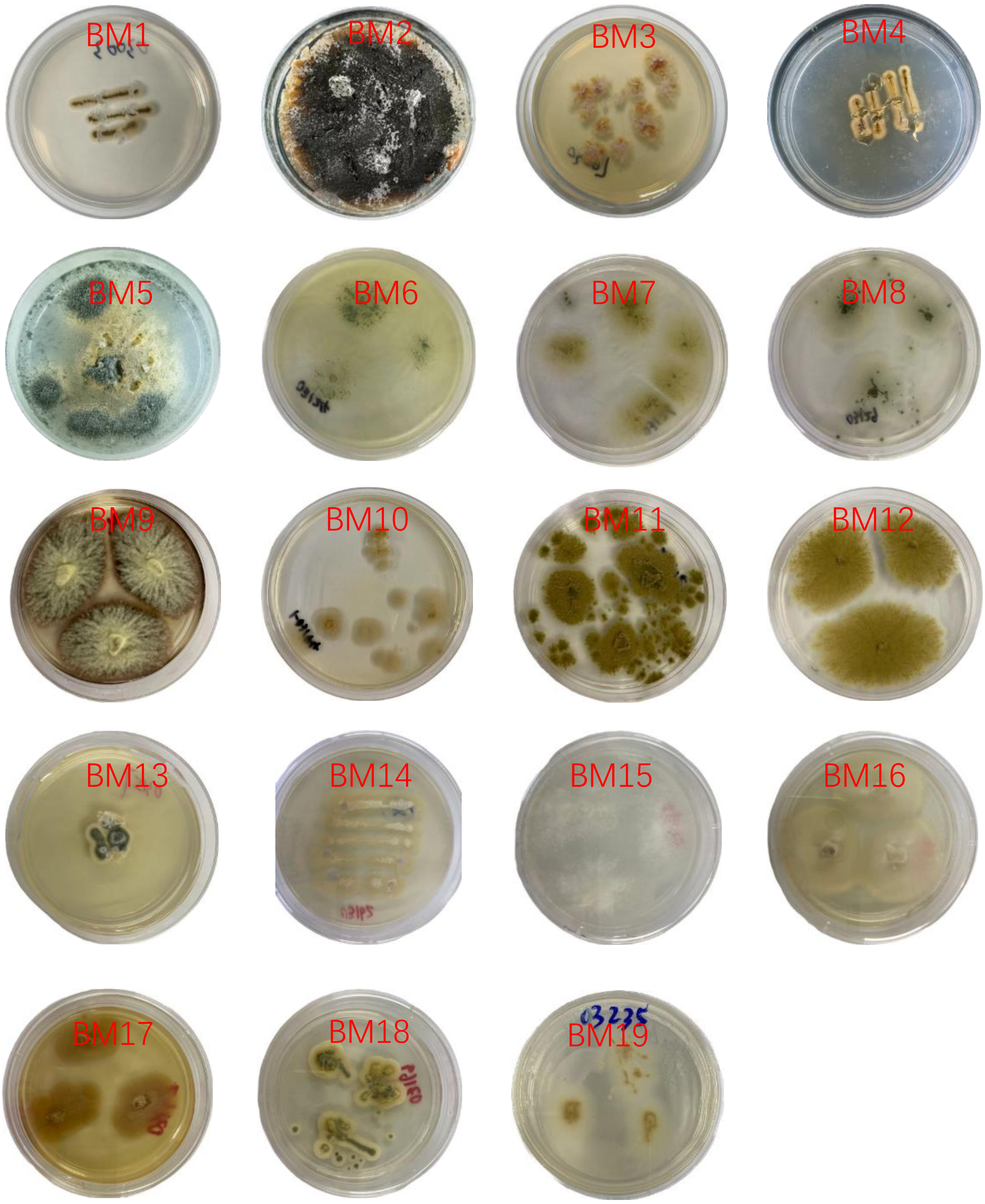

Figure S41 Colony morphologies of fungal strains BM1 to BM19 cultured on PDA medium

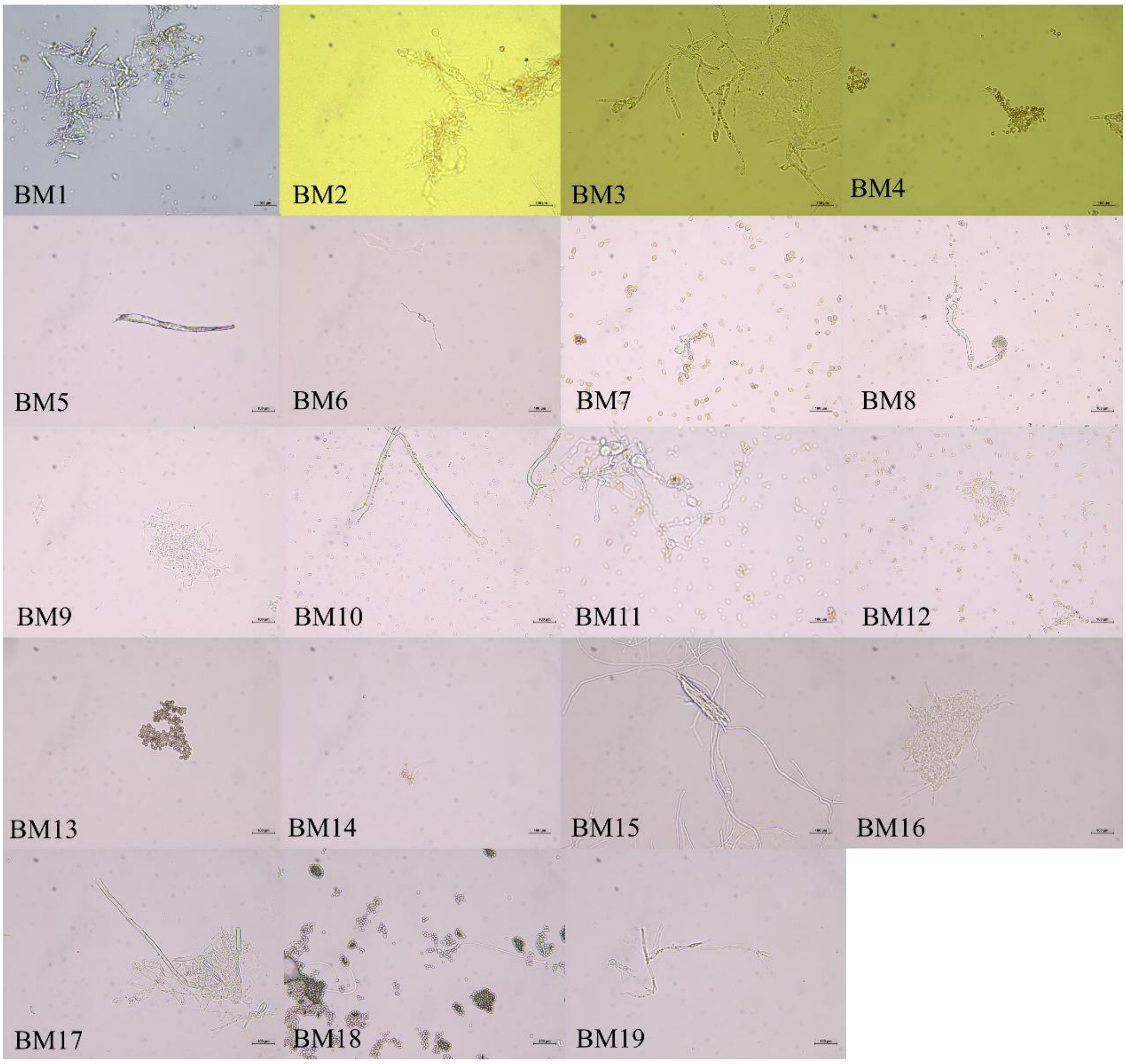

Figure S42 Microscopic micrographs of fungal strains BM1–BM19

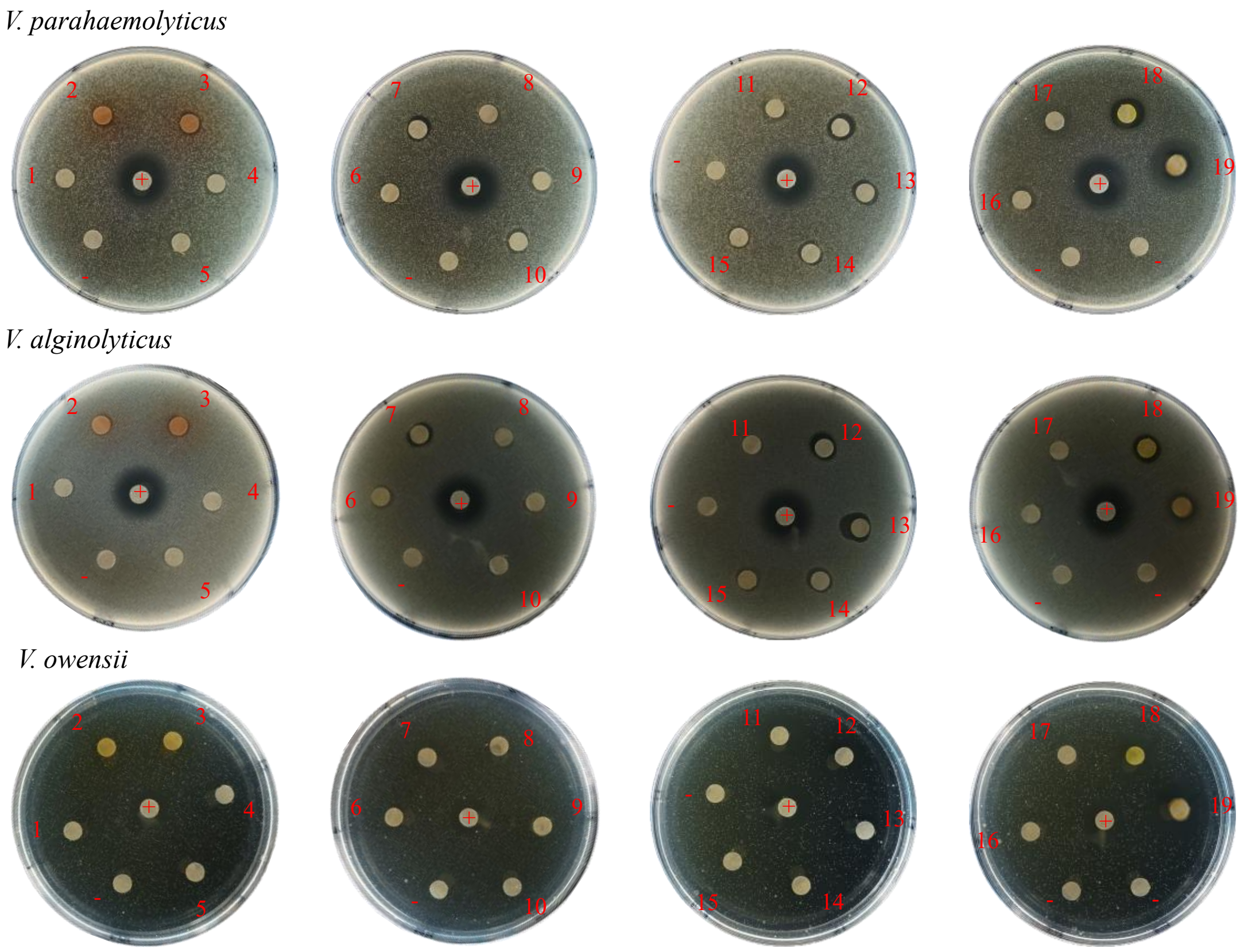

Figure S43 Inhibitory activities of crude extracts of BM1–BM9 against *Vibrio*. Each plate contains paper disks, with “+” indicating the positive control and “–” indicating the negative control.

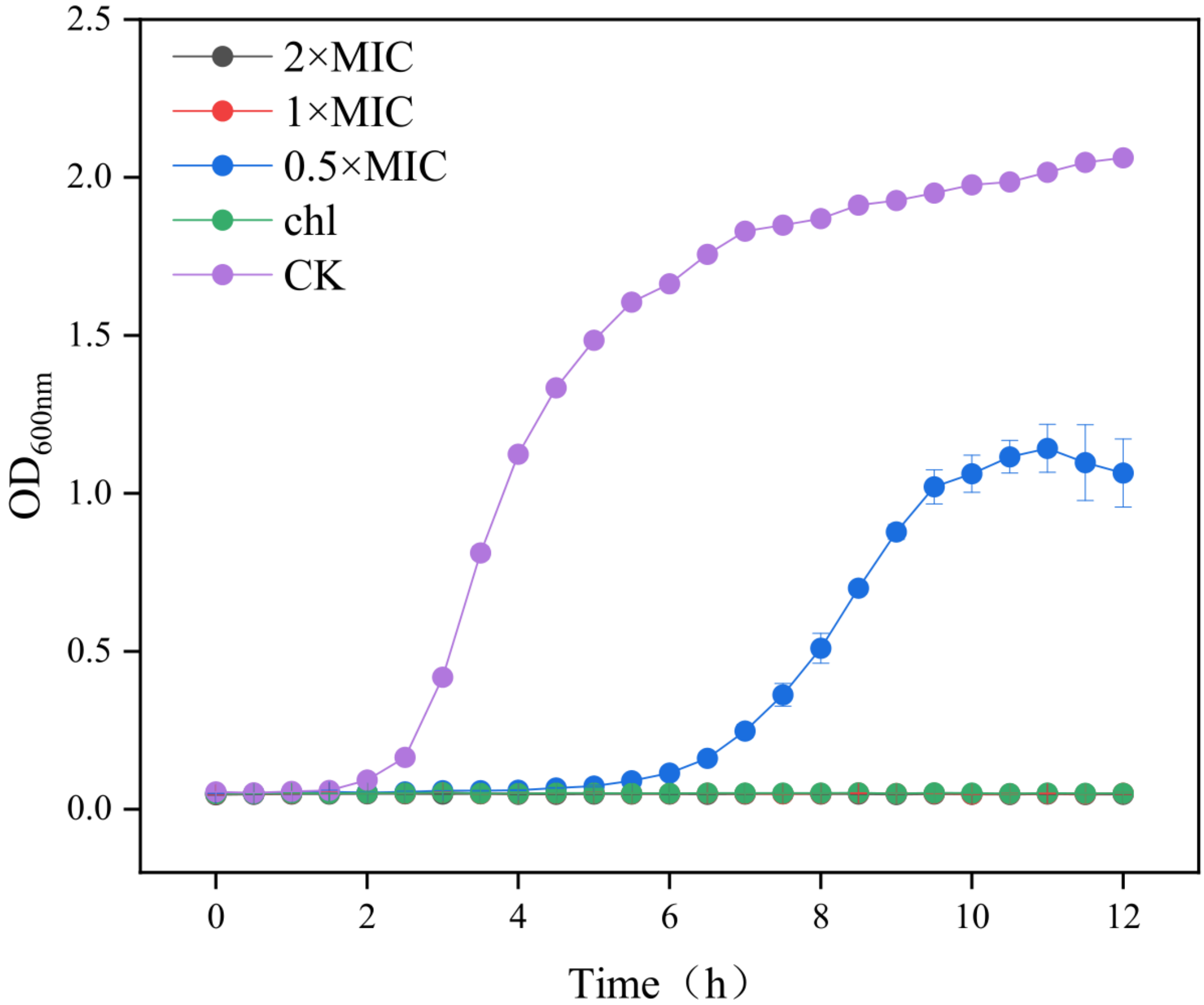

Figure S44 Growth curves of *V. parahaemolyticus* treated by compound **1**.

ITS sequences of strains BM1-19

Mycothermus thermophilus BM1 (GenBank accession no. PZ409761):
CCTTCTACTGATCGAGGTCACCTAGTGCTAAAGACGCAAAAGGTCGCTTT
ACGGCCAGCACCCGCAGCACTCCCAAAGCGAGGTTGTAAGCTACTACGCT
CGGTGTGTCTTGCGAGCCCGCCACTGCATTTCAGGGCCTGCGGCAGCCGC
AGGTCCCCAACACAAGCCCAGGGCTTGATGGTTGAAATGACGCTCGAACA
GGCATGCCCGCCGGAATACCGGCGGGCGCAATGTGCGTTCAAAGATTCGA
TGATTCACTGAATTCTGCAATTCACATTACTTATCGCATTTCGCTGCGTTCT
TCATCGATGCCAGAACCAAGAGATCCGTTGTTGAAAGTTTTGACTTATTCA
GTACATAAACTCAGAGAGGCCATGTGCTGCAAAGGGTTTGGGGTATTCCT
CCGGCGGGAGCCTCCCCCCACGCCGCGGGGGCCGGGGGGATCGGCCGAG
GGGCGCCGAGGCGCCTCCCGCCGAAGCAACAGAAGGGTCGGTTCACAAT
GGTTTGGTGAGTTGTGTAACTCTGTAATGATCCCTCCGCAGGTTCACCCCT
ACGGAA

Corynascus verrucosus BM2 (GenBank accession no. PZ409762):
GGGGGACTCTACTGATCCGAGGTCAACCTTGGGTTATGAAGGTCGTTTAA
CGGCCGGAACCCGCAGCACGCCCTGAGCGAGATGTATGCTACTACGCTCG
GTGTGACTAGCGAGCCCGCCACTGGTTTTCAGGGCCTGCGGACAGCCGCA
GGTCCCCAACACAAGCCGGGGGCTTGATGGTTGAAATGACGCTCGAACAG

GCATGCCCGCCAGGATACTGGCGGGCGCAATGTGCGTTCAAAGATTCGAT
GATTCACTGAATTCTGCAATTCACATTACTTATCGCATTTCGCTGCGTTCTT
CATCGATGCCAGAACCAAGAGATCCGTTGTTGAAAGTTTTGACTTATTCA
GTACAGAAGACTCAGAGTGGCCATAATGTATCAAGAGTTTGGGTACCTCC
GGCGGGCGCCCGCGGTGGGGCCCAGGGGCGCGCGAGGGCGCCGCCCGCC
GAAGCAACGGTTTGGGTAACGTTCACGATGGTTTAGGGAGTTTTGCAACT
CTGTAATGATCCCTCCGCAGGTTCACCCCTACGGA

Aspergillus terreus BM3 (GenBank accession no. PZ409765):
GGTGGGATCCTACTGATCCGAGGTCACCTGGAAAAAACAAGTTGCAAATA
AATGCGTCGGCGGGCGCCGGCCGGGCCTACGGAGCGGAAGACGAAGCCC
CATACGCTCGAGGACCGGACGCGGTGCCGCCGCTGCCTTTCGGGCCCGTC
CCCCGGGAGCCGGGGGACGAGGGCCCAACACACAAGCCGGGCTTGAGGG
CAGCAATGACGCTCGGACAGGCATGCCCCCCGGAATACCAGGGGGCGCA
ATGTGCGTTCAAAGACTCGATGATTCACTGAATTCTGCAATTCACATTAGT
TATCGCATTTCGCTGCGTTCTTCATCGATGCCGGAACCAAGAGATCCATTG
TTGAAAGTTTTAACTGATTGCAAAGAATCACACTCAGACTGCAAGCTTTC
AGAACAGGGTTCATGTTGGGGTCTCCGGCGGGCACGGGCCCGGGGGCGA
GTCGCCCCCCGGCGGCCAGCAACGCTGGCGGGCCCGCCGAAGCAACAAG
GTACAATAGTCACGGGTGGGAGGTTGGGCCATAAAGACCCGCACTCGGTA
ATGATCCTTCCGCAGGTTCACCCTACGGAAG

Thermothelomyces fergusii BM4 (GenBank accession no. PZ409766):
AGGATCTCTACCTGATCCGAGGTCAACCTTGGGGTGTGTTGGCGTTTTACG
GCCGGAACCCGCAGCACGCCCTGAGCGAGGTTTCGCGTTGCTACTACGCT
CGGTGTGACTAGCGAGCCCGCCACTGGTTTTCGGGGCCTGCGGGCGGCCG
CAGGTCCCCAACACAAGCCGTGGGCTTGATGGGCGAAATGACGCTCGAAC
AGGCATGCCCGCCAGGGAGAATCTGGCGGGCGCAATGTGCGTTCAAAGAT
TCGATGATTCACGGAATTCTGCAATTCACATTACTTATCGCATTTCGCTGC
GTTCTTCATCGATGCCAGAACCAAGAGATCCGTTGTTGAAAGTTTTGACTT
ATTCGGTACACAAAAGACTCAGAGCGGCCAACGGGTGTCAGAGAGTTTCT
GTGCAACCTCCGGCGGGCGCCCGCGGGTGTGCGGGCCCAGGGGCGCGCG
GGGGCGCCGCCCGCCGAAGCAACGGTTTAGGGGTAGCGTTCGCGATGGTT
TGGGGGAGTCTTGCAACTCTGTAATGATCCCTCCGCAGGTTACCCCTACGG
AA

Madurella fahalii BM5 (GenBank accession no. PZ409774):
TGGGATTCCTACCTGATCCGAGGTCAACCTTGGGGTAATGGGTGGTTTAA
CGGCCGGAACCCGCAGCACGCCCTGAGCGAGGTGATACTACTACGCTCGG
AGTGACAGCGAGCCCGCCACTGGTTTTCAGGGCCTGCGGGCAGCCGCAGG
TCCCCAACACAAGCCCGGGGGCTTGATGGTTGAAATGACGCTCGAACAGG
CATGCCCGCCAGAATACTGGCGGGCGCAATGTGCGTTCAAAGATTCGATG
ATTCACTGAATTCTGCAATTCACATTACTTATCGCATTTCGCTGCGTTCTTC
ATCGATGCCAGAACCAAGAGATCCGTTGTTGAAAGTTTTGACTTATTAAGT
ACAGAGACTCAGAGAGGCCATACAAAGTACAAGGGTTGTATACCTCCGGC
GGGCGCCCGCGGGGGGCCGAGAGGCGCCGGGGAGGCCCGGGCGCCGCCC

GCCGAAGCAACGGTTTTGGGTATGTTCACAATGGTTTGGGAGTTTTGCAAC
TCTGTAATGATCCCTCCGCAGGTTCACCCTACGGA

Trichoderma longibrachiatum BM6 (GenBank accession no. PZ409775):
TCCTACTGATCCGAGGTCACATTTCAGAAGTTTGGGGTGTTTTACGGCTGT
GGCCGCGCCGCGCTCCCGGTGCGAGTGTGCAAACTACTGCGCAGGAGAGG
CTGCGGCGAGACCGCCACTGTATTTCGGGGGCGGCCCGGTGAGGGGCCGA
TCCCCAACGCCGACCCCCCGGAGGGGTTCGAGGGTTGAAATGACGCTCGG
ACAGGCATGCCCGCCAGAATACTGGCGGGCGCAATGTGCGTTCAAAGATT
CGATGATTCACTGAATTCTGCAATTCACATTACTTATCGCATTTCGCTGCG
TTCTTCATCGATGCCAGAACCAAGAGATCCGTTGTTGAAAGTTTTGATTCA
TTTTCGAGACGCCCGCTAGGGTCGCCGAGAAAGGCTCAGAGCAAAAATAA
AACAGAGCCGCGACGGAGCCGCGACGGAGAGAAAAAAAGAGTTTGGAG
TTGGTCCTCCGGCGGGCGCCATGGGATCCGGGGCTGCGACGCGCCCGGGG
CAAGAGAATCCCGCCGAGGCAACAGATTGGTAACGTTCACATTGGGGTTT
GGGAGTTGTAAACTCGGTAATGATCCCTCCGCAGGTTCACCCCTACGGA

Paecilomyces variotii BM7 (GenBank accession no. PZ409776):
GGGGGATCCTACCTGATCCGAGGTCACCTAAGAGAAATATAGGTGACCGT
GAAGGTCGTGTGGCCAGCAAGCCGGCCGGTTCTACTGAAGCGTGTGACAA
AGCCCCATACGCTCGAGGACCGGACGCGACGCCGCCGCTGCCTTTCGGGC
CCGTCCCCCGGGGGGGGGACGGCGGCCCAACACACCAGCCGGGCTGGAG
GGTTAGCAATGACGCTCGGACAGGCATGCCCCCCGGAATGCCAGGGGGC
GCAATGTGCGTTCAAAGATTCGATGATTCACGGAATTCTGCAATTCACATT
ACTTATCGCATTTCGCTGCGTTCTTCATCGATGCCGGAACCAAGAGATCCG
TTGTTGAAAGTTTTAATTGATTGATTGTATACTCAGACGGCAACCTTCCAG
GCAGCGTTCCAGGGGTCTTCGGCGGGCGCGGGCCCGGGGGCGTGAACCCC
CCGGCGGCCGGGGCGTGAACCACGGCGGGCCCGCCGAAGCAACAGGTGT
CAGGACAACACGGATGGGAGGTTGGGCCCCGAGGGACCCTCACTCGGTA
ATGATCCTTCCGCAGGTTCACCCTACGGAAG

Aspergillus fumigatus BM8 (GenBank accession no. PZ409777):
TCCTTACCTTGATCCGAGGTCACCTTAGAAAAATAAAGTTGGGTGTCGGCT
GGCGCCGGCCGGGCCTACAGAGCAGGTGACAAAGCCCCATACGCTCGAG
GACCGGACGCGGTGCCGCCGCTGCCTTTCGGGCCCGTCCCCCGGGAGAGG
GGGACGGGGGCCCAACACACAAGCCGTGCTTGAGGGCAGCAATGACGCT
CGGACAGGCATGCCCCCCGGAATACCAGGGGGCGCAATGTGCGTTCAAA
GACTCGATGATTCACTGAATTCTGCAATTCACATTACTTATCGCATTTCGC
TGCGTTCTTCATCGATGCCGGAACCAAGAGATCCGTTGTTGAAAGTTTTAA
CTGATTACGATAATCAACTCAGACTGCATACTTTCAGAACAGCGTTCATGT
TGGGGTCTTCGGCGGGCGCGGGCCCGGGGGCGCAAGGCCTCCCCGGCGGC
CGTCGAAACGGCGGGCCCGCCGAAGCAACAAGGTACGATAGACACGGGT
GGGAGGTTGGACCCAGAGGGCCCTCACTCGGTAATGATCCTTCCGCAGGT
TCACCCTACGGAAG

Chaetomium globosum BM9 (GenBank accession no. PZ409778):
CTTACCTGATCGAGGTCACCTTGGGTTAAAAGGTGGTTTAACGGCCGGAA
CCCGCGGCGCGACCAGAGCGAGATGTATGCTACTACGCTCGGTGCGACAG

CGAGCCCGCCACTGCTTTTCAGGGCCTGCGGCAGCCGCAGGTCCCCAACA
CAAGCCCGGGGGCTTGATGGTTGAAATGACGCTCGAACAGGCATGCCCGC
CAGAATGCTGGCGGGCGCAATGTGCGTTCAAAGATTCGATGATTCACTGA
ATTCTGCAATTCACATTACTTATCGCATTTCGCTGCGTTCTTCATCGATGCC
AGAACCAAGAGATCCGTTGTTGAAAGTTTTGACTTATTCAGTACAGAAGA
CTCAGAGAGGCCATAAATTATCAAGAGTTTGGTGACCTCCGGCGGGCGCC
CGCGGTGGGGCCCAGGGGCGCCCGGGGGGTAAACCCCGGGGCCGCCCGC
CGAAGCAACGGTATAGGTAACGTTCACAATGGTTTAGGGAGTTTTGCAAC
TCTGTAATGATCCCTCCGCAGGTTCACCCTACGGA

Aspergillus terreus BM10 (GenBank accession no. PZ409971):

GGGGGATTCTACTGATCGAGGTCACCTGGAAAAAACAAGTTGCAAATAAA
TGCGTCGGCGGGCGCCGGCCGGGCCTACGGAGCGGAAGACGAAGCCCCA
TACGCTCGAGGACCGGACGCGGTGCCGCCGCTGCCTTTCGGGCCCGTCCC
CCGGGAGCCGGGGGACGAGGGCCCAACACACAAGCCGGGCTTGAGGGCA
GCAATGACGCTCGGACAGGCATGCCCCCCGGAATACCAGGGGGCGCAAT
GTGCGTTCAAAGACTCGATGATTCACTGAATTCTGCAATTCACATTAGTTA
TCGCATTTCGCTGCGTTCTTCATCGATGCCGGAACCAAGAGATCCATTGTT
GAAAGTTTTAACTGATTGCAAAGAATCACACTCAGACTGCAAGCTTTCAG
AACAGGGTTCATGTTGGGGTCTCCGGCGGGCACGGGCCCGGGGGCGAGTC
GCCCCCCGGCGGCCAGCAACGCTGGCGGGCCCGCCGAAGCAACAAGGTA
CAATAGTCACGGGTGGGAGGTTGGGCCATAAAGACCCGCACTCGGTAATG
ATCCTTCCGCAGGTTCACCCCTACGGAAG

Paecilomyces variotii BM11 (GenBank accession no. PZ409972):

CAACCGGGGGTTTCCCTACCTGATCCCGAGGTTCAACCTAAGAGAAAAAA
TAGGTGACCGTGAGGTCGCTGGCTGGCCAGCCGGCCGACCCTACCAGAGC
GCGTGACAAAGCCCCATACGCTCGAGGATCGGACGCGGCGCCGCCGCTGC
CTTTCGGGCCCGTCCCCCGGGGGGGACGTCGACCCAACACACCAGCCGGG
CTGGAGGGTTAGCAATGACGCTCGGACAGGCATGCCCCCCGGAATGCCAG
GGGGCGCAATGTGCGTTCAAAGATTCGATGATTCACGGAATTCTGCAATT
CACATTACTTATCGCATTTCGCTGCGTTCTTCATCGATGCCGGAACCAAGA
GATCCGTTGTTGAAAGTTTTAATGATTGATTTTATACTCAGACGGCAACCT
TCAGGGCAGCGTTCGAGGGGTCTTCGGCGGGCGCGGGCCCGGGAGCACA
AGGCCCCCCGGCGGCCGGGCGTGAACCACGGCGGGCCCGCCGAAGCAAC
AGGTGTAGTTCAACACGGATGGGAGGTTGGGCCTCGTGGGACCCTCACTC
GGTAATGATCCTTCCGCAGGTTCACCTACGGAAG

Paecilomyces variotii BM12 (GenBank accession no. PZ410341):

TGGGGGTTCCCTACCTGATCCGAGGTCACCTAAGAGAAAAAATAGGTGAC
CGTGAGGTCGCTGGCTGGCCAGCCGGCCGACCCTACCAGAGCGCGTGACA
AAGCCCCATACGCTCGAGGATCGGACGCGGCGCCGCCGCTGCCTTTCGGG
CCCGTCCCCCGGGGGGGACGTCGACCCAACACACCAGCCGGGCTGGAGG
GTTAGCAATGACGCTCGGACAGGCATGCCCCCCGGAATGCCAGGGGGCGC
AATGTGCGTTCAAAGATTCGATGATTCACGGAATTCTGCAATTCACATTAC
TTATCGCATTTCGCTGCGTTCTTCATCGATGCCGGAACCAAGAGATCCGTT
GTTGAAAGTTTTAATGATTGATTTTATACTCAGACGGCAACCTTCAGGGCA

GCGTTCGAGGGGTCTTCGGCGGGCGCGGGCCCGGGAGCACAAGGCCCCCC
GGCGGCCGGGCGTGAACCACGGCGGGCCCGCCGAAGCAACAGGTGTAGT
TCAACACGGATGGGAGGTTGGGCCTCGTGGGACCCTCACTCGGTAATGAT
CCTTCCGCAGGTTCACCTACGGAA

Aspergillus versicolor BM13 (GenBank accession no. PZ410342):
TGGGGTTCCCCTACCTGATCCGAGGTCACCTGAAGAAAATGGTTGGAGAC
GTCGGCTGGCGCCCGGCCGGCCCTAGTCGAGCGGGTGACAAAGCCCCATA
CGCTCGAGGACCGGACACGGTGCCGCCGCTGCCTTTCGGGCCCGTCCCCC
GGGGGGGGACGACGACCCAACACACAAGCCGGGCTTGATGGGCAGCAAT
GACGCTCGGACAGGCATGCCCCCCGGAATGCCAGGGGGCGCAATGTGCGT
TCAAAGACTCGATGATTCACTGAATTCTGCAATTCACATTACTTATCGCAG
TTCGCTGCGTTCTTCATCGATGCCGGAACCAAGAGATCCATTGTTGAAAGT
TTTGACTGATTTTATATTCAGACTCAGACTGCATCACTCTCAGGCATGAAG
TTCAGTAGTCCCCGGCGGCTCGCCCCCGAGGGGGTTCCCCGCCGAAGCAA
CAGTGTTAGGTATTCACGGGTGGGAGGTTGGGCGCCCGGAGGCAGCCCGC
ACTCAGTAATGATCCTTCCGCAGGTTCACCTACGGAAG

Penicillium polonicum BM14 (GenBank accession no. PZ410346):
CGCGGGGTTCCCTACCTGATCCGAGGTCAACCTGGATAAAAATTTGGGTT
GATCGGCAAGCGCCGGCCGGGCCTACAGAGCGGGTGACAAAGCCCCATA
CGCTCGAGGACCGGACGCGGTGCCGCCGCTGCCTTTCGGGCCCGTCCCCC
GGAATCGGAGGACGGGGCCCAACACACAAGCCGGGCTTGAGGGCAGCAA
TGACGCTCGGACAGGCATGCCCCCCGGAATACCAGGGGGCGCAATGTGCG
TTCAAAGACTCGATGATTCACTGAATTTGCAATTCACATTACGTATCGCAT
TTCGCTGCGTTCTTCATCGATGCCGGAACCAAGAGATCCGTTGTTGAAAGT
TTTAAATAATTTATATTTTCACTCAGACTTCAATCTTCAGACAGAGTTCGG
GGGTGTCTTCGGCGGGCGCGGGCCCGGGGGCGTGAGCCCCCCGGCGGCCA
GTAAAGGCGGGCCCGCCGAAGCAACAAGGTAAAATAAACACGGGTGGGA
GGTTGGACCCAAAGGGCCCTCACTCGGTAATGATCCTTCCGCAGGTTCAC
CTACGGAAG

Trametes hirsuta BM15 (GenBank accession no. PZ410345):
CCTACCTGGATTTGAGGTCAGATGTTGAAAGATTGTCCTAATGGACGGTTA
GAAGCTCGCCAAAACGCTTCACGGTCACAGCGTAGACAATTATCACACTG
AGAGCCGATCCGCACGGAATCAAGCTAATGCATTCAAGAGGAGCCGACC
GCTAGGGCCAGCAAGCCTCCAAATCCAAGCCCATAGATCACAAGGACTTA
TGGGTTGAGAATTTCATGACACTCAAACAGGCATGCTCCTCGGAATACCA
AGGAGCGCAAGGTGCGTTCAAAGATTCGATGATTCACTGAATTCTGCAAT
TCACATTACTTATCGCATTTCGCTGCGTTCTTCATCGATGCGAGAGCCAAG
AGATCCGTTGCTGAAAGTTGTATTAAGATGCGTTAGACGCGTTTACATTCT
GATACTTTAGAGTTTGTAGTGTACATAGGCCGGCAGAATGCTCCCAGCCC
GGAGGCTAGAAACCCACGCCAACCTACAGTAAGTGCACAGGTGTAGAGT
GGATGAGCAGGGCGTGCACATGCCTCGGAAGGCCAGCAACAACCCATTTC
AAAACTCGTTAATGATCCTTCCGCAGGTTCACCCCTACGGAAG

Aspergillus hiratsukae BM16 (GenBank accession no. PZ410350):

CGGGGTTCCTACCTGATCGAGGTCACCTTAGAAAAATAAAGTTGGGGTGT
CGGCTGGCGCCGGCCGGGCCTACAGAGCGGGTGACAAAGCCCCATACGCT
CGAGGATCGGACGCGGTGCCGCCGCTGCCTTTCGGGCCCGTCCCCGGGGA
GAACCGGGGACGGGGGCCCAACACACAAGCCGTGCTTGAGGGCAGCAAT
GACGCTCGGACAGGCATGCCCCCCGGAATACCAGGGGGCGCAATGTGCGT
TCAAAGACTCGATGATTCACTGAATTCTGCAATTCACATTACTTATCGCAT
TTCGCTGCGTTCTTCATCGATGCCGGAACCAAGAGATCCGTTGTTGAAAGT
TTTAACTGATTATGATAATCAAACTCAGACTGCATACTTTCAGAACAGCGT
TCATGTTGGGGTCTTCGGCGGGCGCGGGCCCGGGGGCGCGAGGCCTCCCC
GGCGGCCGTTCGAAAACGGCGGGCCCGCCGAAGCAACAAGGTACAATAG
ACACGGGTGGGAGGTTGGACCCAGAGGGCCCAGCACTCGGTAATGATCCT
TCCGCAGGTTCACCCTACGGAAG

Penicillium malachiteum BM17 (GenBank accession no. PZ410349):
CTCCCCTACCTTGATCCGAGGTCACCTGGAAAAAAACAAGTTGCAAATAA
ATGCGTCGGCGGGCGCCGGCCGGGCCTACGGAGCGGAAGACGAAGCCCC
ATACGCTCGAGGACCGGACGCGGTGCCGCCGCTGCCTTTCGGGCCCGTCC
CCCGGGAGCCGGGGGACGAGGGCCCAACACACAAGCCGGGCTTGAGGGC
AGCAATGACGCTCGGACAGGCATGCCCCCCGGAATACCAGGGGGCGCAA
TGTGCGTTCAAAGACTCGATGATTCACTGAATTCTGCAATTCACATTAGTT
ATCGCATTTCGCTGCGTTCTTCATCGATGCCGGAACCAAGAGATCCATTGT
TGAAAGTTTTAACTGATTGCAAAGAATCACACTCAGACTGCAAGCTTTCA
GAACAGGGTTCATGTTGGGGTCTCCGGCGGGCACGGGCCCGGGGGCGAGT
CGCCCCCCGGCGGCCAGCAACGCTGGCGGGCCCGCCGAAGCAACAAGGT
ACAATAGTCACGGGTGGGAGGTTGGGCCATAAAGACCCGCACTCGGTAAT
GATCCTTCCGCAGGTTCACCTACGGAAG

Talaromyces radicus BM18 (GenBank accession no. PZ410422):
CCTTACCTGATCCGAGGTCAACCGGAAGGAGACGAGCCCGAAAGGGCCG
CCTGGGGGAAGACCAGCGCCGACCGAGTCCCTCCCGAGCGGGTGACAGA
GCCCCATACGCTCGAGGACCCGACGCGGCGCCGCCACTGCCTTTGGGGCG
TGTCCCCGGGGGGACAGCGCCCAACACCCAGCCGTGCTGGAGGGCAGAA
ATGACGCTCGGACAGGCATGCCCCCCGGAATGCCAGGGGGCGCAATGTGC
GTTCAAAGATTCGATGATTCACGGAATTCTGCAATTCACATTACTTATCGC
ATTTCGCTGCGTTCTTCATCGATGCCGGAACCAAGAGATCCGTTGTTGAAA
GTTTTAATGATTCAATATCACACTCAGACTCACTGTTCAGACAGGGTTCCA
GGGCGCTTCGGCGGGCGCGGGCCCGGGGGCAGAAGCCCCCCGGCGACCG
GGGCGAGGCCCCAGTGGGCCCGCCGAAGCAACGCGGTAACGGTAGACAC
GGGTGGGAGGTTGGGCTCATTCGAACCCGCACTCGGTAATGATCCTTCCG
CAGGTTCACCTACGGAAG

Aspergillus sclerotiorum BM19 (GenBank accession no. KC461519):
CCGAGGTCAACCTGGAGAAATAGTTGGTTGCTTTTCAGCGTCGGCCAGCG
CCGGCCGGGCCTACAAGAGCGGGTGACAAAGCCCCATACGCTCGAGGAC
CGGACGCGGTGCCGCCGCTGCCTTTCGGGCCCGTCCCCGGGGGGACGAC
GACCCAACACACAAGCCGTGCTTGAGGGCAGCAATGACGCTCGGACAGG
CATACCCCCCGGAATACCAGGGGGTGCAATGTGCGTTCAAAGACTCGATG

ATTCACTGAATTCTGCAATTCACATTAATTATCGCATTTCGCTGCGTTCTTC
ATCGATGCCGGAACCAAGAGATCCATTGTTGAAAGTTTTAACTGATTGCG
ATACAATCGAACTCAGACAACAAAACTTCAGACAGTGTTCACGTTGGTGT
CTCCGGCGGGCGCTCGCCTAGGGAGGGGGGTTTGACGCCCCCCCGGCGGC
CGCTTGCGCGGCGGGCCCGCCGAAGCAACAAGGTACGGTATACACGGGT
GGGAGGTTGGGCCCCGAGGGACCCTCACTCAGTAATGATCCTTCCGCAGG
TTCACCCTACGGAAG
